# Classification of time-reversal-invariant crystals with gauge structures

**DOI:** 10.1038/s41467-023-36447-7

**Published:** 2023-02-10

**Authors:** Z. Y. Chen, Zheng Zhang, Shengyuan A. Yang, Y. X. Zhao

**Affiliations:** 1 National Laboratory of Solid State Microstructures and Department of Physics, Nanjing University, 210093 Nanjing, China; 2 Research Laboratory for Quantum Materials, Singapore University of Technology and Design, Singapore, 487372 Singapore; 3 Collaborative Innovation Center of Advanced Microstructures, Nanjing University, 210093 Nanjing, China

**Keywords:** Topological matter, Acoustics

## Abstract

A peculiar feature of quantum states is that they may embody so-called projective representations of symmetries rather than ordinary representations. Projective representations of space groups-the defining symmetry of crystals-remain largely unexplored. Despite recent advances in artificial crystals, whose intrinsic gauge structures necessarily require a projective description, a unified theory is yet to be established. Here, we establish such a unified theory by exhaustively classifying and representing all 458 projective symmetry algebras of time-reversal-invariant crystals from 17 wallpaper groups in two dimensions-189 of which are algebraically non-equivalent. We discover three physical signatures resulting from projective symmetry algebras, including the shift of high-symmetry momenta, an enforced nontrivial Zak phase, and a spinless eight-fold nodal point. Our work offers a theoretical foundation for the field of artificial crystals and opens the door to a wealth of topological states and phenomena beyond the existing paradigms.

## Abstract (web summary)

Projective representations of crystal symmetries are indispensable for understanding artificial crystals. Here, authors establish a unified theory of projective crystal symmetries with time-reversal invariance, and construct models for all 458 projective symmetry algebras for the 17 two-dimensional wallpaper groups.

## Introduction

Symmetry groups and their representations are at the heart of physics. When going from classical to quantum physics, a classical symmetry group *G* becomes represented in the Hilbert space, where it makes no physical difference if all states are multiplied by a global phase. It follows that the representation allows an extra phase factor, i.e., for *g*_1_, *g*_2_ ∈ *G*, their representations *ρ*(*g*_1_) and *ρ*(*g*_2_) may satisfy *ρ*(*g*_1_)*ρ*(*g*_2_) = *ν*(*g*_1_, *g*_2_)*ρ*(*g*_1_, *g*_2_) with *ν*(*g*_1_, *g*_2_) ∈ *U*(1). This is known as the projective representation of *G*, and the phase factors *ν* called the factor system for this representation. As a well-known example, classifying the projective representations of Poincaré group for elementary particles leads to the two types of particles, bosons and fermions, corresponding to two distinct factor systems^1^.

**Fig. 1:**
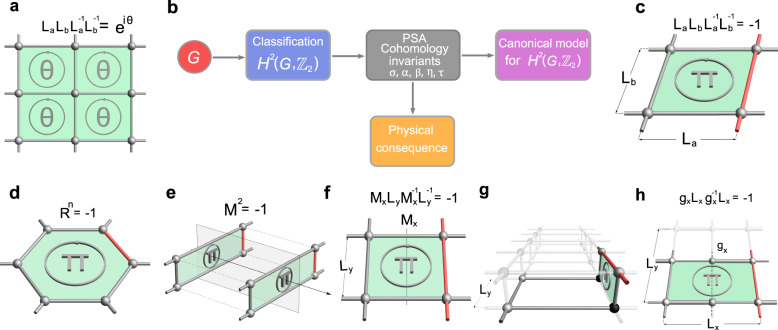
Logic chart and flux realizations of projective algebraic relations. **a** Gauge flux in a lattice requires the space group symmetries to be projectively represented. **b** Work flow diagram of this work. Given a space group *G*, the classification of projective representations are given by ${H}^{2}(G,{{\mathbb{Z}}}_{2})$. These representations are captured by projective symmetry algebras (PSAs) with a complete set of cohomology invariants, from which we construct a canonical model that can realize all possible PSAs, and derive nontrivial physical consequences. **c**–**h** Illustrate the construction method for realizing the five basic classes of PSAs (cohomology invariants). Specifically, (**c**, **d**, **e**, **h**) are for nontrivial *σ*, *α*, *β*, *τ*, respectively, and (**f** and **g**) are for nontrivial *η*. In (**g**), the bond connecting the two black sites can be either present or absent, and the total flux through the vertical and horizontal rectangular plaquettes is required to be *π*. Here, the *π* flux in a plaquette is realized by a negative hopping amplitude (marked by red color) on an edge of that plaquette.

The defining symmetries for crystals are the space groups. What is the physical meaning of a projective representation in this context? Consider a spinless quantum particle on a two-dimensional (2D) lattice as shown in Fig. 1a. A projective representation for the lattice translations allows (hereafter, we use bold letters to denote the represented symmetry operators) ${{\mathsf{L}}}_{a}{{\mathsf{L}}}_{b}{{\mathsf{L}}}_{a}^{-1}{{\mathsf{L}}}_{b}^{-1}={e}^{i\theta }$, from which one observes that the extra phase factor corresponds to a gauge flux through the lattice. This shows that projective representations of space groups are associated with gauge fluxes, and somewhat explains the previous ignorance of them in textbooks on solid-state physics^2^. Because most electronic crystals are free of gauge flux, one can show that their descriptions are restricted to ordinary representations. Nevertheless, it was recognized that rich gauge-flux configurations can emerge in certain strongly correlated spin systems, where projective representations of space groups are needed for their description^3–8^.

The rise of artificial crystals in recent years completely changes the situation. Artificial crystals cover a wide range of systems, such as acoustic, photonic, mechanical, circuit, and cold-atom systems^9–19^. Most artificial crystals intrinsically preserve time-reversal (*T*) symmetry, which allows fluxes 0 and *π* over the lattices. A salient feature is that these lattice gauge fluxes can be readily engineered. Recent works showed that these fluxes modify the physics in a fundamental way and projective representations are indispensable for understanding artificial crystals^20–25^. This urgently calls for a unified theory of projective representations of symmetries for *T*-invariant crystals, which constitutes the foundation of the whole field.

In this work, we develop such a theory and predict its distinguishing consequences. First, we characterize all possible projective symmetry algebras (PSAs) with time-reversal symmetry for any space group. This is demonstrated by 458 PSAs—189 of which are algebraically independent—for all 17 wallpaper groups in two dimensions. Then, we show all the 2D PSAs can be systematically realized by lattice models with appropriate gauge fluxes. Finally, we present three signature results of PSAs, including the shift of high-symmetry momenta, an enforced nontrivial Zak phase, and a spinless eightfold nodal point.

## Results

### Projective symmetry algebras with time-reversal invariance

We start by presenting a general result that reduces the problem for systems with time reversal symmetry *T*. Let *G* be the space group, then the total symmetry group is $G\times {{{{{{{{\mathcal{Z}}}}}}}}}_{2}^{T}$, where ${{{{{{{{\mathcal{Z}}}}}}}}}_{2}^{T}=\{E,T\}$ is the two-element group generated by *T*. Mathematically, the classification of all possible factor systems for this group corresponds to the second group cohomology ${H}^{2,c}(G\times {{{{{{{{\mathcal{Z}}}}}}}}}_{2}^{T},U(1))$^26^. We have proven that due to the anti-unitary character of *T*, ${H}^{2,c}(G\times {{{{{{{{\mathcal{Z}}}}}}}}}_{2}^{T},U(1))\cong {H}^{2}(G,\, {{\mathbb{Z}}}_{2})\times {H}^{2}({{{{{{{{\mathcal{Z}}}}}}}}}_{2}^{T},{{\mathbb{Z}}}_{2})$ with ${{\mathbb{Z}}}_{2}=\{\pm 1\}$ (see Methods). Hence, the computation is simplified to deriving ${H}^{2}(G,\, {{\mathbb{Z}}}_{2})$, since it is known that ${H}^{2}({{{{{{{{\mathcal{Z}}}}}}}}}_{2}^{T},\, {{\mathbb{Z}}}_{2})\cong {{\mathbb{Z}}}_{2}=\{\pm 1\}$ distinguishes integer and half integer spins, respectively. This means we only need to consider *G* with factors restricted to ${{\mathbb{Z}}}_{2}=\{\pm 1\}$. Our discussion below focuses on spinless systems, which are pertinent to most artificial crystals. It can be directly extended to spinful systems, as we shall comment at the end.

The second group cohomology ${H}^{2}(G,\, {{\mathbb{Z}}}_{2})$ can be derived from the abstract group cohomology theory, e.g., from the twisted tensor product of the cochain complex of the translation subgroup and that of the point group^27^. Considering the 17 wallpaper groups for 2D, the results are listed in the second column of Table 1. Besides the classification, we also need to know the content of each ${H}^{2}(G,\, {{\mathbb{Z}}}_{2})$, namely, the concrete algebraic relations satisfied by the symmetry operators, which are called the PSAs, because they are directly related to the physics of a system. We have worked out all PSAs in terms of generators of each group, as listed in the fourth column of Table 1. The technical details are given in Supplementary Note 2. The classification is complete, meaning that any *T*-invariant crystal system in two dimensions must belong to one of the PSAs listed here.

**Table 1:** Projective symmetry algebras of 17 wallpaper groups

*G*	${H}^{2}(G,{{\mathbb{Z}}}_{2})$	Generators	Cohomology invariants	*N*_*G*_
*P*1	$\,{{\mathbb{Z}}}_{2}$	L_*a*_, L_*b*_	$$\begin{array}{c}\sigma={{\mathsf{L}}}_{a}{{\mathsf{L}}}_{b}{{\mathsf{L}}}_{a}^{-1}{{\mathsf{L}}}_{b}^{-1}.\end{array}$$	2
*P*2	${{\mathbb{Z}}}_{2}^{4}$	L_*a*_, L_*b*_, R	$$\begin{array}{c}{\alpha }_{1}={{\mathsf{R}}}^{2},{\alpha }_{2}={({{\mathsf{L}}}_{a}{\mathsf{R}})}^{2},{\alpha }_{3}={({{\mathsf{L}}}_{b}{\mathsf{R}})}^{2},{\alpha }_{4}={({{\mathsf{L}}}_{a}{{\mathsf{L}}}_{b}{\mathsf{R}})}^{2}.\end{array}$$	5
*Pm*	${{\mathbb{Z}}}_{2}^{4}$	L_*x*_, L_*y*_, M_*x*_	$$\begin{array}{c}{\beta }_{1}={{\mathsf{M}}}_{x}^{2},{\beta }_{2}={({{\mathsf{L}}}_{x}{{\mathsf{M}}}_{x})}^{2},{\eta }_{1}={{\mathsf{M}}}_{x}{{\mathsf{L}}}_{y}{{\mathsf{M}}}_{x}^{-1}{{\mathsf{L}}}_{y}^{-1},{\eta }_{2}=({{\mathsf{L}}}_{x}{{\mathsf{M}}}_{x}){{\mathsf{L}}}_{y}{({{\mathsf{L}}}_{x}{{\mathsf{M}}}_{x})}^{-1}{{\mathsf{L}}}_{y}^{-1}.\\ \end{array}$$	10
*Pg*	$\,{{\mathbb{Z}}}_{2}$	L_*x*_, g_*x*_	$$\begin{array}{c}\tau={{\mathsf{g}}}_{x}{{\mathsf{L}}}_{x}{{\mathsf{g}}}_{x}^{-1}{{\mathsf{L}}}_{x}.\end{array}$$	2
*Cm*	${{\mathbb{Z}}}_{2}^{2}$	L_*a*_, L_*b*_, M	$$\begin{array}{c}\sigma={{\mathsf{L}}}_{a}{{\mathsf{L}}}_{b}{{\mathsf{L}}}_{a}^{-1}{{\mathsf{L}}}_{b}^{-1},\beta={{\mathsf{M}}}^{2},1={\mathsf{M}}{{\mathsf{L}}}_{a}{{\mathsf{M}}}^{-1}{{\mathsf{L}}}_{b}^{-1}.\end{array}$$	4
*Pmm*	${{\mathbb{Z}}}_{2}^{8}$	L_*x*_, L_*y*_, M_*x*_, M_*y*_	$$\begin{array}{l}{\alpha }_{1}={({{\mathsf{M}}}_{x}{{\mathsf{M}}}_{y})}^{2},{\alpha }_{2}={({{\mathsf{L}}}_{x}{{\mathsf{M}}}_{x}{{\mathsf{M}}}_{y})}^{2},{\alpha }_{3}={({{\mathsf{L}}}_{y}{{\mathsf{M}}}_{x}{{\mathsf{M}}}_{y})}^{2},{\alpha }_{4}={({{\mathsf{L}}}_{x}{{\mathsf{L}}}_{y}{{\mathsf{M}}}_{x}{{\mathsf{M}}}_{y})}^{2},\\ {\beta }_{1}={{\mathsf{M}}}_{x}^{2},{\beta }_{2}={({{\mathsf{L}}}_{x}{{\mathsf{M}}}_{x})}^{2},{\beta }_{3}={{\mathsf{M}}}_{y}^{2},{\beta }_{4}={({{\mathsf{L}}}_{y}{{\mathsf{M}}}_{y})}^{2}.\end{array}$$	51
*Pmg*	${{\mathbb{Z}}}_{2}^{4}$	g_*y*_, L_*y*_, M_*x*_	$$\begin{array}{c}{\alpha }_{1}={({{\mathsf{M}}}_{x}{{\mathsf{g}}}_{y})}^{2},{\alpha }_{2}={({{\mathsf{L}}}_{y}{{\mathsf{M}}}_{x}{{\mathsf{g}}}_{y})}^{2},\beta={{\mathsf{M}}}_{x}^{2},\eta={{\mathsf{M}}}_{x}{{\mathsf{L}}}_{y}{{\mathsf{M}}}_{x}^{-1}{{\mathsf{L}}}_{y}^{-1}.\end{array}$$	12
*Pgg*	${{\mathbb{Z}}}_{2}^{2}$	g_*x*_, g_*y*_	$$\begin{array}{c}{\alpha }_{1}={({{\mathsf{g}}}_{x}{{\mathsf{g}}}_{y})}^{2},{\alpha }_{2}={({{\mathsf{g}}}_{x}{{\mathsf{g}}}_{y}^{-1})}^{2}.\end{array}$$	3
*Cmm*	${{\mathbb{Z}}}_{2}^{5}$	L_*a*_, M_*x*_, M_*y*_	$$\begin{array}{l}{\alpha }_{1}={({{\mathsf{M}}}_{x}{{\mathsf{M}}}_{y})}^{2},{\alpha }_{2}={({{\mathsf{L}}}_{a}{{\mathsf{M}}}_{x}{{\mathsf{M}}}_{y})}^{2},{\alpha }_{3}={({{\mathsf{L}}}_{a}{{\mathsf{M}}}_{x}{{\mathsf{L}}}_{a}^{-1}{{\mathsf{M}}}_{y})}^{2},{\beta }_{1}={{\mathsf{M}}}_{x}^{2},{\beta }_{2}={{\mathsf{M}}}_{y}^{2}.\end{array}$$	18
*P*4	${{\mathbb{Z}}}_{2}^{3}$	L_*x*_, R	$$\begin{array}{c}{\alpha }_{1}={{\mathsf{R}}}^{4},{\alpha }_{2}={({{\mathsf{L}}}_{x}{{\mathsf{R}}}^{2})}^{2},{\alpha }_{3}={({{\mathsf{L}}}_{x}{\mathsf{R}})}^{4}.\end{array}$$	6
*P*4*m*	${{\mathbb{Z}}}_{2}^{6}$	L_*x*_, R, M	$$\begin{array}{l}{\alpha }_{1}={{\mathsf{R}}}^{4},{\alpha }_{2}={({{\mathsf{L}}}_{x}{{\mathsf{R}}}^{2})}^{2},{\alpha }_{3}={({{\mathsf{L}}}_{x}{\mathsf{R}})}^{4},{\beta }_{1}={{\mathsf{M}}}^{2},{\beta }_{2}={({\mathsf{R}}{\mathsf{M}})}^{2},{\beta }_{3}={({{\mathsf{L}}}_{x}{\mathsf{M}})}^{2}.\end{array}$$	40
*P*4*g*	${{\mathbb{Z}}}_{2}^{3}$	g_*y*_, R	$$\begin{array}{l}{\alpha }_{1}={{\mathsf{R}}}^{4},{\alpha }_{2}={({{\mathsf{g}}}_{y}^{2}{{\mathsf{R}}}^{2})}^{2},{\beta }_{1}={({{\mathsf{g}}}_{y}{\mathsf{R}})}^{2}.\end{array}$$	6
*P*3	${{\mathbb{Z}}}_{2}$	L_*a*_, L_*b*_, R	$$\begin{array}{l}\sigma={{\mathsf{L}}}_{a}{{\mathsf{L}}}_{b}{{\mathsf{L}}}_{a}^{-1}{{\mathsf{L}}}_{b}^{-1},1={\mathsf{R}}{{\mathsf{L}}}_{a}{{\mathsf{R}}}^{-1}{{\mathsf{L}}}_{b}^{-1}{{\mathsf{L}}}_{a},1={\mathsf{R}}{{\mathsf{L}}}_{b}{{\mathsf{R}}}^{-1}{{\mathsf{L}}}_{a},1={{\mathsf{R}}}^{3}.\end{array}$$	2
*P*3*m*1	${{\mathbb{Z}}}_{2}^{2}$	L_*a*_, L_*b*_, R, M	$$\begin{array}{l}\sigma={{\mathsf{L}}}_{a}{{\mathsf{L}}}_{b}{{\mathsf{L}}}_{a}^{-1}{{\mathsf{L}}}_{b}^{-1},\beta={{\mathsf{M}}}^{2}={({\mathsf{R}}{\mathsf{M}})}^{2}={({{\mathsf{L}}}_{a}{\mathsf{M}})}^{2},1={\mathsf{R}}{{\mathsf{L}}}_{a}{{\mathsf{R}}}^{-1}{{\mathsf{L}}}_{b}^{-1}{{\mathsf{L}}}_{a},\\ 1={\mathsf{R}}{{\mathsf{L}}}_{b}{{\mathsf{R}}}^{-1}{{\mathsf{L}}}_{a},1={{\mathsf{R}}}^{3}.\end{array}$$	4
*P*31*m*	${{\mathbb{Z}}}_{2}^{2}$	L_*a*_, L_*b*_, R, M	$$\begin{array}{l}\sigma={{\mathsf{L}}}_{a}{{\mathsf{L}}}_{b}{{\mathsf{L}}}_{a}^{-1}{{\mathsf{L}}}_{b}^{-1}={{\mathsf{L}}}_{a}{\mathsf{M}}{{\mathsf{L}}}_{a}^{-1}{{\mathsf{M}}}^{-1},\beta={{\mathsf{M}}}^{2}={({\mathsf{R}}{\mathsf{M}})}^{2},1={\mathsf{R}}{{\mathsf{L}}}_{a}{{\mathsf{R}}}^{-1}{{\mathsf{L}}}_{b}^{-1}{{\mathsf{L}}}_{a},\\ 1={\mathsf{R}}{{\mathsf{L}}}_{b}{{\mathsf{R}}}^{-1}{{\mathsf{L}}}_{a},1={{\mathsf{R}}}^{3}.\end{array}$$	4
*P*6	${{\mathbb{Z}}}_{2}^{2}$	L_*a*_, R	$$\begin{array}{c}{\alpha }_{1}={{\mathsf{R}}}^{6},{\alpha }_{2}={({{\mathsf{L}}}_{a}{{\mathsf{R}}}^{3})}^{2},1={({{\mathsf{L}}}_{a}{{\mathsf{R}}}^{2})}^{3}.\end{array}$$	4
*P*6*m*	${{\mathbb{Z}}}_{2}^{4}$	L_*a*_, R, M	$$\begin{array}{c}{\alpha }_{1}={{\mathsf{R}}}^{6},{\alpha }_{2}={({{\mathsf{L}}}_{a}{{\mathsf{R}}}^{3})}^{2},{\beta }_{1}={{\mathsf{M}}}^{2}={({{\mathsf{L}}}_{a}{\mathsf{M}})}^{2},{\beta }_{2}={({\mathsf{R}}{\mathsf{M}})}^{2},1={({{\mathsf{L}}}_{a}{{\mathsf{R}}}^{2})}^{3}.\end{array}$$	16

The first column ‘*G*’ lists the names of 17 wallpaper groups, and the second column ‘${H}^{2}(G, \, {{\mathbb{Z}}}_{2})$’ presents the corresponding second cohomology groups. The chosen generators of each wallpaper group are given in the third column. The PSAs and the cohomology invariants are presented in the fourth column. L, R, M and g denote translation, rotation, mirror and glide reflection, respectively. *σ*, *α*, *β*, *η* and *τ* are the five classes of cohomology invariants valued in ${{\mathbb{Z}}}_{2}=\{\pm 1\}$. The last column ‘*N*_*G*_’ lists the number of equivalence classes of PSAs for each wallpaper group.

Interestingly, each ${H}^{2}(G,{{\mathbb{Z}}}_{2})$ is a product of ${{\mathbb{Z}}}_{2}$, i.e., ${H}^{2}(G,{{\mathbb{Z}}}_{2})\cong {{\mathbb{Z}}}_{2}^{n}$. Meanwhile, we find that the corresponding PSA can be captured by a complete set of *n*${{\mathbb{Z}}}_{2}$-valued cohomology invariants, which are denoted by *σ*, *α*, *β*, *η*, and *τ* in Table 1. The specific meaning of these symbols will be explained in a while. Here, one can easily check that they are indeed cohomology invariants, by noting that they are unchanged when multiplying symmetry operators by arbitrary ${{\mathbb{Z}}}_{2}$ phases ± 1. The ordinary representation just corresponds to the case with all invariants being + 1.

It should be noted that for each *G* the PSAs classified by ${H}^{2}(G,{{\mathbb{Z}}}_{2})$ have redundancies for their abstract algebraic structures, because often *G* has nontrivial automorphisms such that two nonequivalent factor systems lead to equivalent algebraic structures. We screen out all nonequivalent algebras, of which the numbers (*N*_*G*_) are listed in the last column of Table 1. We find that there are 189 non-equivalent algebraic structures out of the 458 PSAs.

To illustrate our theory, we take group *P*2 as an example. The set of generators of *P*2 consists of two unit translations *L*_*a*_, *L*_*b*_ and the rotation *R* by *π* (along an out-of-plane axis). Its group algebras are expressed in terms of the four combinations, *R*, *L*_*a*_*R*, *L*_*b*_*R* and *L*_*a*_*L*_*b*_*R*, each of which is squared to 1. According to Table 1, ${H}^{2}(P2,\, {{\mathbb{Z}}}_{2})\cong {{\mathbb{Z}}}_{2}^{4}$, so there are four cohomology invariants *α*_*i*_ (*i* = 1, 2, 3, 4), corresponding to the four PSA relations:

1 $${{\mathsf{R}}}^{2}={\alpha }_{1},\,{({{\mathsf{L}}}_{a}{\mathsf{R}})}^{2}={\alpha }_{2},\,{({{\mathsf{L}}}_{b}{\mathsf{R}})}^{2}={\alpha }_{3},\,{({{\mathsf{L}}}_{a}{{\mathsf{L}}}_{b}{\mathsf{R}})}^{2}={\alpha }_{4}.$$

Since any permutation of the four twofold rotations above gives an isomorphic PSA, there are only five equivalence classes of PSAs, and each class is specified by how many *α*^’s equal − 1.

Our result shows that the 17 wallpaper groups together with *T* symmetry can generate 458 × 2 PSAs (the additional factor 2 is from spin), which is much richer than the case of Poincaré group with only twofold classification. Physically, this is due to the reduced symmetry which allows more gauge flux configurations.

### Flux realizations of projective symmetry algebras

After completing the classification, our next task is to develop a construction method to realize each of the PSAs. This is important for two purposes. First, it serves as a validity check for our results in Table 1 and demonstrates that each PSA can indeed be realized in a physical system. Second, it provides guidance for the experimental realization of nontrivial PSAs in artificial crystals.

Our construction is via mapping each PSA in Table 1 to a specific gauge flux pattern. In this process, we distinguish five classes of cohomology invariants in PSAs, corresponding to the five symbols *σ*, *α*, *β*, *η*, and *τ* in Table 1. The flux lattices for them are illustrated in Fig. 1c–h, and are elucidated below. The technical details for how these flux lattices represent PSAs can be found in Methods.

(*i*) The first class refers to $\sigma={{\mathsf{L}}}_{a}{{\mathsf{L}}}_{b}{{\mathsf{L}}}_{a}^{-1}{{\mathsf{L}}}_{b}^{-1}$ for the translation subgroup. *σ* = ± 1 corresponds respectively to flux 0 and *π* through the plaquette spanned by L_*a*_ and L_*b*_, as illustrated in Fig. 1c.

(*ii*) The second class concerns cohomology invariants *α* of symmorphic rotational symmetries. That is, *α* = R^*n*^ for an *n*-fold rotation *R*, where *α* = ± 1 corresponds to flux 0 or *π* through the plaquette invariant under the rotation, as in Fig. 1d.

(*iii*) The third class corresponds to the square of a mirror reflection *M*, i.e., *β* = M^2^. It turns out that M^2^ = − 1 cannot be realized on lattices with only nearest neighbor hoppings within one layer. We propose to realize it either by second neighbor hopping as in Ref. ^28^ or on a bilayer lattice with *π* flux through the interlayer plaquettes, as shown in Fig. 1e.

(*iv*) The fourth class (*η* invariants) is on relations between translations and reflections. For example, $\eta={{\mathsf{M}}}_{x}{{\mathsf{L}}}_{y}{{\mathsf{M}}}_{x}^{-1}{{\mathsf{L}}}_{y}^{-1}$, and *η* = ± 1 corresponds to flux 0 or *π* through the plaquette in Fig. 1f that preserves L_*y*_ and M_*x*_. Moreover, if ${{\mathsf{M}}}^{2}={{\mathsf{M}}}_{x}{{\mathsf{L}}}_{y}{{\mathsf{M}}}_{x}^{-1}{{\mathsf{L}}}_{y}^{-1}=-1$, we may design the flux pattern as in Fig. 1g.

(*v*) The fifth class consists of invariants *τ* that extend the algebraic relations between translations and glide reflections, e.g., $\tau={{\mathsf{g}}}_{x}{{\mathsf{L}}}_{x}{{\mathsf{g}}}_{x}^{-1}{{\mathsf{L}}}_{x}$. As illustrated in Fig. 1h, *τ* = ± 1 respectively corresponds to flux 0 or *π* through the area spanned by the translation and glide reflection, which is half of the plaquette spanned by unit translations. It appears only for *Pg* group in Table 1.

With the above building blocks, we can systematically translate the cohomology invariants into fluxed lattices and obtain models realizing each of PSAs in Table 1. In this way, we have constructed a “canonical” lattice model for each wallpaper group *G*, in the sense that all PSAs for *G* can be realized in this single model, by simply varying the 0/*π* flux distribution in the lattice. In Methods, we categorize the 17 wallpaper groups into five classes to briefly introduce how the canonical lattice models are constructed.

**Fig. 2:**
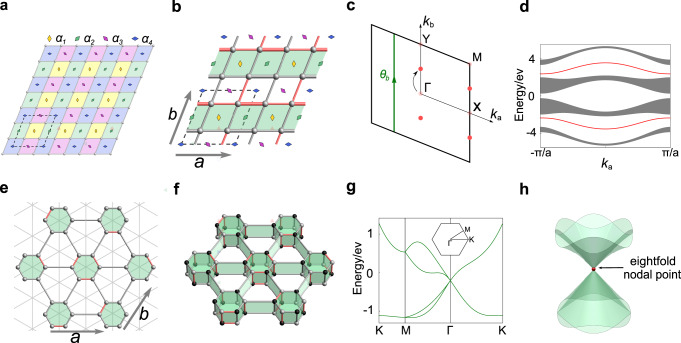
Models and physical consequences of projective symmetry algebras. **a** The canonical model for *P*2. The dashed line marks the unit cell. The four classes of translation-related rotation centers are colored in red, green, purple and blue, respectively. Each rotation center is the center of a plaquette shadowed with the same color of the rotation center. The four colors correspond to the four *α*-invariants in Table 1. **b** The model of *P*2 that realizes the projective symmetry algebra (PSA) with *α*_1_ = *α*_2_ = 1 and *α*_3_ = *α*_4_ = − 1. The dashed line marks the unit cell, which contains four sites. The bonds with red color have a negative hopping amplitude, which makes the shaded plaquettes having a *π* flux. **a** and **b** Are lattice vectors. Here, we added a dimerization pattern in hopping to open spectral gaps, as in (**d**). **c** Due to the PSA in (**b**), high-symmetry momenta are shifted and the Zak phase *θ*_*b*_ over a ***G***_*b*_-periodic path must be nontrivial. Here, *k*_*a*,*b*_ are the wave-vector components for the lattice vectors (**a** and **b**) in (**b**). **d** Spectrum of the model in (**b**) on the slab geometry with the *b* dimension confined. The spectrum is parametrized by *k*_*a*_. The in-gap edge states are colored in red, which arise from the nontrivial Zak phase. To construct a canonical model for *P*3*m*1, we first build a one-layer lattice model in (**e**) to accommodate the *σ* invariant. Then, we double it into a bilayer model in (**f**) to further accommodate the *β* invariant. In (**f**), black and white colors mean the two sites are inequivalent, e.g., they may have different on-site energies. (**g**) Band structure of a *P*3*m*1 model in (**f**), which exhibits an eightfold nodal point at Γ. Note that along Γ-*M* (Γ-*K*), each band is twofold (fourfold) degenerate. **h** Dispersion in the vicinity of the eightfold degenerate nodal point.

As an example, consider *P*2 group with four *α* invariants. The algebraic relation for each *α*_*i*_ in Table 1 corresponds to a twofold rotation center in the unit cell, as illustrated in Fig. 2a. Under lattice translation, each *α*_*i*_ is associated with a class of translation-related rotation centers, which are distinguished by four colors in Fig. 2a. Then, the canonical model can be constructed with each plaquette hosting a unique rotation center (see Fig. 2a), corresponding to the dual lattice of the lattice of rotation centers. Each *α*_*i*_ = ± 1 can then be realized by inserting flux 0 or *π* into the corresponding class of plaquettes.

**Fig. 3:**
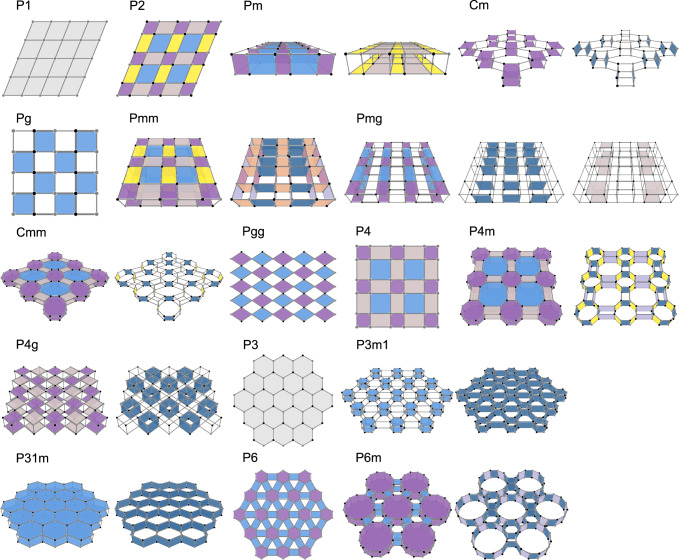
Illustration for the canonical models of 17 wallpaper groups. For each wallpaper group, the cohomology invariants are realized by independent fluxes Φ_*a*_ ∈ {0, *π*} on the lattice, which are distinguished by different colors.

The canonical models for all 17 wallpaper groups are illustrated in Fig. 3, and are explicitly constructed in Supplementary Note 3. For each wallpaper group in Fig. 3, the cohomology invariants correspond to independent fluxes in the lattice model, and we distinguish the fluxes by different colors. This is consistent with the number 2^*n*^ of PSAs, with *n* the number of colors in each lattice model.

### Physical consequences of projective symmetry algebras

Our revealed PSAs can lead to a wealth of new physics, beyond conventional systems based on ordinary representations. Below, we present three remarkable consequences for demonstration.

(*1*) *Shift of high-symmetry points*. In ordinary band structures, high-symmetry points are located either at the center (Γ point) or on the boundary of Brillouin zone (BZ)^2^. In contrast, with PSAs, the high-symmetry points are redistributed, and they can be at non-central points in the interior of BZ.

For instance, continue with the example of *P*2 group. Let’s consider the PSA with *α*_1_ = *α*_2_ = 1 and *α*_3_ = *α*_4_ = − 1 (see the canonical model realization in Fig. 2b). Clearly, in this case the two translations L_*a*_ and L_*b*_ commute as usual, and therefore the BZ is unchanged. However, since $R{L}_{b}{R}^{-1}={L}_{b}^{-1}$ is modified to ${\mathsf{R}}{{\mathsf{L}}}_{b}{{\mathsf{R}}}^{-1}=-{{\mathsf{L}}}_{b}^{-1}$ by the fluxes, the *R*-invariant momenta are transformed from (0, 0), (0, *π*), (*π*, 0) and (*π*, *π*) to (0, ± *π*/2) and (*π*, ± *π*/2), as illustrated in Fig. 2c (see discussion in Methods).

(*2*) *Enforced nontrivial Zak phase*. While ordinary crystal symmetries may protect topological structures of energy bands, we discover that some PSAs can even *enforce* nontrivial topological structures. That is, once the PSA is realized, certain topological invariant is *guaranteed* to be nontrivial.

Here, we give one example of this fascinating phenomena, again using the *P*2 group. Let us consider the PSAs with ${{\mathsf{R}}}^{2}={({{\mathsf{L}}}_{a}{\mathsf{R}})}^{2}=\alpha$ and ${({{\mathsf{L}}}_{b}{\mathsf{R}})}^{2}={({{\mathsf{L}}}_{a}{{\mathsf{L}}}_{b}{\mathsf{R}})}^{2}=-\alpha$, which can be realized by the canonical model with the flux configuration in Fig. 2b. The PSAs lead to ${\mathsf{R}}{{\mathsf{L}}}_{b}{{\mathsf{R}}}^{-1}=-{{\mathsf{L}}}_{b}^{-1}$ for both *α* = ± 1. From this relation, one can show that the anti-unitary operator R*T* will shift momentum ***k*** to ***k*** + ***G***_*b*_/2 with ***G***_*b*_ the reciprocal translation vector corresponding to L_*b*_ (see Methods).

Now, consider the effect of R*T* on a single energy band with eigenstates $\left | {\psi }_{{{{{{{{\boldsymbol{k}}}}}}}}}\right\rangle$. Recall that spacetime inversion symmetry can quantize the Berry phase, also known as the Zak phase, along any periodic path in the BZ to be either 0 or *π*^29^. In contrast, here, R*T* with (R*T*)^2^ = *α* exerts a stronger constraint on the Zak phase, i.e., it completely determines the Zak phase *θ*_*b*_ along any ***G***_*b*_-periodic path as

2 $${\theta }_{b}=i\ln \alpha \,{{{{{{\mathrm{mod}}}}}}}\,\,2\pi,$$

due to the nontrivial action of R*T* discussed above (see Methods). This result means: if *α* = − 1, the Zak phase is enforced to be nontrivial. This is confirmed by the concrete model in Fig. 2b. This model has four isolated bands, and each band is enforced to have a nontrivial Zak phase *π* along *k*_*b*_. Hence, there must be topological edge states within the first and the third energy gaps, as shown in Fig. 2d.

(*3*) *Eightfold degenerate nodal point*. Highly degenerate nodal points protected by crystal symmetries have been a hot topic. Without including the twofold degeneracy of spin-1/2, the highest degeneracy protected by wallpaper groups is fourfold^30^. Here, we find that PSA can achieve a degeneracy of eightfold, beyond any ordinary representations.

This is exemplified by the PSA of *P*3*m*1 with ${{\mathsf{L}}}_{a}{{\mathsf{L}}}_{b}{{\mathsf{L}}}_{a}^{-1}{{\mathsf{L}}}_{b}^{-1}=-1$ and M^2^ = − 1 (see Table 1). The canonical model is illustrated in Fig. 2e and f. Since L_*a*_ does not commute with L_*b*_, we choose ${{\mathsf{L}}}_{a}^{2}$ and ${{\mathsf{L}}}_{b}^{2}$ to generate an invariant subgroup of *P*3*m*1, and the BZ is specified by ${{\mathsf{L}}}_{a}^{2}=-{e}^{i{{{{{{{\boldsymbol{k}}}}}}}}\cdot {{{{{{{{\boldsymbol{e}}}}}}}}}_{a}}$ and ${{\mathsf{L}}}_{b}^{2}=-{e}^{i{{{{{{{\boldsymbol{k}}}}}}}}\cdot {{{{{{{{\boldsymbol{e}}}}}}}}}_{b}}$ under the Fourier transform, with ***e***_*a*,*b*_ being the translation vectors of ${{\mathsf{L}}}_{a,b}^{2}$. At high-symmetry point Γ, the little co-group is given by ${{\mathbb{Z}}}_{2}^{2}\rtimes {D}_{3}\times {{{{{{{{\mathcal{Z}}}}}}}}}_{2}^{T}$, where ${{\mathbb{Z}}}_{2}^{2}$ are generated by L_*a*,*b*_. This little co-group is projectively represented with factors inherited from that of *P*3*m*1. We find that it has two 4D irreducible representations and one 8D irreducible representation. The latter gives the eightfold nodal point, which is indeed confirmed via a concrete model as illustrated in Fig. 2g and h.

## Discussion

In conclusion, we have established a unified theory for *T*-invariant crystals. Particularly, we classified all PSAs of wallpaper groups, developed a general construction method, presented canonical models to realize each PSA, and revealed remarkable physical consequences. The theory can be directly extended to 3D space groups. Our work provides a solid foundation for the study of artificial crystals and opens the door to a wealth of new physics beyond the current paradigm based ordinary symmetry representations.

Notably, although our focus here is on spinless systems (which most artificial crystals belong to), the generalization to spinful systems is straightforward. This is because in the presence of *T*-invariance, it is always sufficient to consider ${{\mathbb{Z}}}_{2}$-valued factor systems, as stressed above. Then, in addition to the phases arising from fluxes, one only needs to take care of reflections and rotations of spin-1/2 by 2*π*, which lead to the phase − 1. Hence, all the cohomology invariants in classes (*ii*) and (*iii*) are reversed. Formally, we may just replace each *α* and *β* by (−1)^2*s*^*α* and (−1)^2*s*^*β*, respectively, with *s* = 0 and 1/2 for spinless and spin-1/2 cases.

Finally, we note that our theory of PSAs is based on two fundamental principles of physics: (a) Physical systems are classified by symmetries (Landau’s paradigm); and (b) Symmetries are projectively represented in a physical system (Wigner’s principle). Hence, the PSAs derived here are general and classify all *T*-invariant crystal systems, including not only artificial crystals, but also real materials, strongly correlated spin systems, and beyond.

## Methods

### Projective representations with time-reversal symmetry

In the main text, we emphasized that with *T* symmetry, the phase factors of space group symmetries can be constrained to be valued in ${{\mathbb{Z}}}_{2}$. Here, we present a proof for this proposition.

Let us enlarge the space group *G* by including *T* with *T*^2^ = 1. Then, each group element can be written as *gT*^*a*^ with *g* ∈ *G* and *a* = 0, 1. Suppose that under the projective representation *ρ*, the phase factor *λ* arises through

3 $$\rho ({g}_{1}{T}^{{a}_{1}})\rho ({g}_{2}{T}^{{a}_{2}})=\lambda ({g}_{1}{T}^{{a}_{1}},{g}_{2}{T}^{{a}_{2}})\rho ({g}_{1}{g}_{2}{T}^{{a}_{1}+{a}_{2}}).$$

We shall prove that by appropriately modifying the phase of each operator *ρ*(*gT*^*a*^), we can always transform $\lambda ({g}_{1}{T}^{{a}_{1}},{g}_{2}{T}^{{a}_{2}})$ into the form,

4 $$\tilde{\lambda }({g}_{1}{T}^{{a}_{1}},{g}_{2}{T}^{{a}_{2}})=\nu ({g}_{1},{g}_{2})\omega ({T}^{{a}_{1}},{T}^{{a}_{2}}),$$

where *ν*(*g*_1_, *g*_2_), $$\omega ({T}^{{a}_{1}},{T}^{{a}_{2}})\in {{\mathbb{Z}}}_{2}$$.

We start with observing that for all *g* ∈ *G*,

5 $$\rho (g)\rho (T)=\lambda (g,T)\rho (gT)=\frac{\lambda (g,T)}{\lambda (T,g)}\,\rho (T)\rho (g),$$

which motivates us to modify the phase of each *ρ*(*g*) as

6 $$\tilde{\rho }(g):=\sqrt{\frac{\lambda (T,g)}{\lambda (g,T)}}\,\rho (g),$$

Note that *ρ*(*T*) is an anti-unitary operator, i.e., *ρ*(*T*)*c* = c* *ρ*(*T*) for $c\in {\mathbb{C}}$. Hence,

7 $$\tilde{\rho }(g)\rho (T)=\rho (T)\tilde{\rho }(g).$$

We further modify the operators for the other half of group elements as

8 $$\tilde{\rho }(gT):=\sqrt{\lambda (g,T)\,\lambda \,(T,g)}\rho (gT),$$

for all *g* ∈ *G*. Note that $\tilde{\rho }(T)=\rho (T)$. Then, one observes that

9 $$\tilde{\rho }(g)\tilde{\rho }(T)=\tilde{\rho }(gT).$$

Let $\tilde{\lambda }$ denote the phase factor for $\tilde{\rho }$. Restricting on *G*, $\tilde{\lambda }$ satisfies

10 $$\tilde{\rho }({g}_{1})\tilde{\rho }({g}_{2})=\tilde{\lambda }({g}_{1},{g}_{2})\tilde{\rho }({g}_{1}{g}_{2})$$

for all *g*_1_, *g*_2_ ∈ *G*. The left-hand side commutes with $\tilde{\rho }(T)$, so does the right-hand side. Hence, $\nu :=\tilde{\lambda }{|}_{G\times G}\in {{\mathbb{Z}}}_{2}=\{\pm 1\}$. On the other hand, $\tilde{\lambda }(T,T)$ appears in

11 $$\tilde{\rho }(T)\tilde{\rho }(T)=\tilde{\lambda }(T,T)1.$$

Clearly, $\tilde{\lambda }(T,T)$ commutes with $\tilde{\rho }(T)$, and therefore $\omega (T,T):=\tilde{\lambda }(T,T)\in {{\mathbb{Z}}}_{2}$.

Finally, it is straightforward to check that

12 $$\tilde{\rho }({g}_{1}{T}^{{a}_{1}})\tilde{\rho }({g}_{2}{T}^{{a}_{2}})\,	=\,\tilde{\rho }({g}_{1})\tilde{\rho }({T}^{{a}_{1}})\tilde{\rho }({g}_{2})\tilde{\rho }({T}^{{a}_{2}})\\ 	=\,\tilde{\rho }({g}_{1})\tilde{\rho }({g}_{2})\tilde{\rho }({T}^{{a}_{1}})\tilde{\rho }({T}^{{a}_{2}})\\ 	=\,\nu ({g}_{1},{g}_{2})\tilde{\rho }({g}_{1}{g}_{2})\omega ({T}^{{a}_{1}},{T}^{{a}_{2}})\tilde{\rho }({T}^{{a}_{1}+{a}_{2}})\\ 	=\,\nu ({g}_{1},{g}_{2})\omega ({T}^{{a}_{1}},{T}^{{a}_{2}})\tilde{\rho }({g}_{1}{g}_{2}{T}^{{a}_{1}+{a}_{2}}).$$

This concludes the proof of our proposition. In the proof, we have repeatedly used the relations: $\tilde{\rho }(g)\tilde{\rho }(T)=\tilde{\rho }(gT)$ and $\tilde{\rho }(g)\tilde{\rho }(T)= \tilde{\rho }(T)\tilde{\rho }(g)$.

### Projective symmetry algebras and gauge fluxes

Let us consider a set of lattice sites and hopping amplitudes among them, which give rise to a tight-binding model,

13 $$\hat{H}=\mathop{\sum}\limits_{ij}{H}_{ij}{a}_{i}^{{{{\dagger}}} }{a}_{j}.$$

Here, ${a}_{i}^{{{{\dagger}}} }$ and *a*_*j*_ are the particle creation and annihilation operators at sites *i* and *j*, respectively. *H*_*ij*_ represents the hopping amplitudes *t*_*ij*_ from site *j* to *i* if *i* ≠ *j* and the onsite energy *ϵ*_*i*_ at site *i* if *i* = *j*. *H* is a Hermitian matrix and called the one-particle Hamiltonian of the tight-binding model.

Each hopping amplitude *t*_*ij*_ may have a phase ${e}^{i{\phi }_{ij}}$ (such that ${t}_{ij}=|{t}_{ij}|{e}^{i{\phi }_{ij}}$), which is called the gauge connection of the lattice model. Particularly, here we consider the ${{\mathbb{Z}}}_{2}$ gauge connections with *ϕ*_*ij*_ ∈ {0, *π*}. For each closed loop *C* formed by successive hoppings, one can compute the product *W*_*C*_ of the phases of all the hopping amplitudes involved. *W*_*C*_ is called the Wilson loop operator of the loop *C*, and the gauge flux Φ_*C*_ through the loop *C* is given by ${W}_{C}={e}^{-i{{{\Phi }}}_{C}}$. For the ${{\mathbb{Z}}}_{2}$ gauge field, we have *W*_*C*_ ∈ { ± 1} and Φ_*C*_ = {0, *π*}.

For each site *i*, we may change the phase of ${a}_{i}^{{{{\dagger}}} }$ for each *i* by an arbitrary ${e}^{i{\theta }^{i}}$. Particularly, *θ*^*i*^ is valued in {0, *π*} for the ${{\mathbb{Z}}}_{2}$ gauge field considered here. Accordingly, the hopping amplitudes are transformed as ${t}_{ij}\mapsto {e}^{i{\theta }^{i}}{t}_{ij}{e}^{-i{\theta }^{j}}$, which is called a gauge transformation. An immediate result is that ${W}_{C}={e}^{-i{{{\Phi }}}_{C}}$ is invariant under any gauge transformation. This can be seen from that the ending site of a hopping is the starting site of the next hopping in a loop *C*, and therefore all phase changes involved are cancelled out. To summarize, the gauge fluxes are gauge-invariant quantities, whereas the gauge connections are not.

Only gauge-invariant quantities are physical. In the current case, the gauge flux configuration completely determines the physics of the model. Hence, a spatial transformation *R* that leaves the crystal and the gauge flux configuration invariant is regarded as a symmetry of the system. However, *R* does not necessarily preserve the gauge-connection configuration *A*. After the action of *R*, *A* is generally changed to another one ${A}^{{\prime} }$. Since the two gauge-connection configurations *A* and ${A}^{{\prime} }$ describe the same flux configuration, they are related by a gauge transformation G_*R*_. On the lattice, *R* is represented by a matrix indexed by lattice sites, which we still denote by *R*. The gauge transformation G_*R*_ is a diagonal matrix with ${[{{\mathsf{G}}}_{R}]}_{ii}={e}^{i{\theta }_{R}^{i}}$, i.e., with the phase assigned to the *i*th site. Then, the *physical* symmetry operator in this case should be the combination

14 $${\mathsf{R}}={{\mathsf{G}}}_{R}R.$$

That is, after the spatial transformation *R*, the gauge transformation G_*R*_ is needed to recover the original gauge connection configuration *A*. Notably, it is R = G_*R*_*R* that commutes with the Hamiltonian *H*, i.e.,

15 $$[{\mathsf{R}},\, H]=0.$$

The commutation relation is equivalent to the requirement,

16 $${t}_{ij}={{\mathsf{G}}}_{R}(i){t}_{{R}^{-1}(i){R}^{-1}(j)}{{\mathsf{G}}}_{R}^{*}(j),$$

where ${G}_{R}(i)={e}^{i{\theta }_{R}^{i}}$, namely the phase assigned to site *i*, and *R*(*i*) is the site transformed from *i* by *R*.

Then, we consider the successive action of two spatial symmetries, ${{\mathsf{R}}}_{1}={{\mathsf{G}}}_{{R}_{1}}{R}_{1}$ and ${{\mathsf{R}}}_{2}={{\mathsf{G}}}_{{R}_{2}}{R}_{2}$. There are two natural operators to implement the action, namely, ${{\mathsf{G}}}_{{R}_{1}}{R}_{1}{{\mathsf{G}}}_{{R}_{2}}{R}_{2}$ and ${{\mathsf{G}}}_{{R}_{12}}{R}_{12}$ with *R*_12_ = *R*_1_*R*_2_. Their difference is ${{{\Delta }}}_{{\mathsf{G}}}({R}_{1},{R}_{2})={{\mathsf{G}}}_{{R}_{1}}{R}_{1}{{\mathsf{G}}}_{{R}_{2}}{R}_{1}^{-1}/{{\mathsf{G}}}_{{R}_{1}{R}_{2}}$. Δ_G_(*R*_1_, *R*_2_) is a diagonal matrix with *i*th diagonal entry being ${{\mathsf{G}}}_{{R}_{1}}(i){{\mathsf{G}}}_{{R}_{2}}({R}_{1}^{-1}(i))/{{\mathsf{G}}}_{{R}_{1}{R}_{2}}(i)$, and therefore represents a gauge transformation. It is clear that Δ_G_(*R*_1_, *R*_2_) commutes with all possible symmetry-preserving Hamiltonians. Particularly, let us presume the usual case that *H* is a connected lattice model, i.e., any two sites are connected by hoppings. The presumption sufficiently leads to the fact that Δ_G_(*R*_1_, *R*_2_) is proportional to the identity matrix, namely ${[{{{\Delta }}}_{{\mathsf{G}}}({R}_{1},{R}_{2})]}_{ij}=\nu ({R}_{1},{R}_{2}){\delta }_{ij}$ with $\nu ({R}_{1},{R}_{2})\in {{\mathbb{Z}}}_{2}\subset U(1)$, i.e., the physical symmetry operators satisfy the PSA,

17 $${{\mathsf{R}}}_{1}{{\mathsf{R}}}_{2}=\nu ({R}_{1},{R}_{2}){{\mathsf{R}}}_{12}.$$

If *ν* and ${\nu }^{{\prime} }$ are related by transforming R to ${{\mathsf{R}}}^{{\prime} }=\chi (R){\mathsf{R}}$ with *χ*(*R*) ∈ *U*(1) or ${{\mathbb{Z}}}_{2}$, the two PSAs belong to the same cohomology class. It must be noted that the cohomology class of such a PSA is independent of the choice of gauge connections, and is solely determined by the flux configuration.

### Realization of cohomology invariants

Based on the general discussions in the last section, we now show the flux lattices in Fig. 1c–h can realize the five classes of cohomology invariants, respectively.

(*i*) Let us start with the cohomology invariant $\sigma={{\mathsf{L}}}_{a}{{\mathsf{L}}}_{b}{{\mathsf{L}}}_{a}^{-1}{{\mathsf{L}}}_{b}^{-1}$. Since

18 $${{\mathsf{L}}}_{a}{{\mathsf{L}}}_{b}{{\mathsf{L}}}_{a}^{-1}{{\mathsf{L}}}_{b}^{-1}	=\,{{\mathsf{G}}}_{a}{L}_{a}{{\mathsf{G}}}_{b}{L}_{b}{({{\mathsf{G}}}_{a}{L}_{a})}^{-1}{({{\mathsf{G}}}_{b}{L}_{b})}^{-1}\\ 	=\,{{\mathsf{G}}}_{a}({L}_{a}{{\mathsf{G}}}_{b}{L}_{a}^{-1}){L}_{b}{{\mathsf{G}}}_{a}{L}_{b}^{-1}{({{\mathsf{G}}}_{b}{L}_{b})}^{-1},$$

the algebraic relation is equivalent to

19 $${{\mathsf{G}}}_{a}(i){{\mathsf{G}}}_{b}({L}_{a}^{-1}(i)){{\mathsf{G}}}_{a}^{*}({L}_{b}^{-1}(i)){{\mathsf{G}}}_{b}^{*}(i)=\sigma$$

for any lattice site *i*. For the lattice model in Fig. 1c, we have from (16) the relations

20 $${t}_{23} \,=\, {t}_{14}{{\mathsf{G}}}_{a}(2){{\mathsf{G}}}_{a}^{*}(3),\quad {t}_{43}={t}_{12}{{\mathsf{G}}}_{b}(4){{\mathsf{G}}}_{b}^{*}(3),$$

which implies

21 $${e}^{i{\phi }_{23}}\,=\,{e}^{i{\phi }_{14}}{{\mathsf{G}}}_{a}(2){{\mathsf{G}}}_{a}^{*}(3),\,\,\,{e}^{i{\phi }_{43}}={e}^{i{\phi }_{12}}{{\mathsf{G}}}_{b}(4){{\mathsf{G}}}_{b}^{*}(3).$$

Here, 1, 2, 3, 4 label the four sites in Fig. 1c, which are counted counterclockwise from the lower left corner. The flux through the rectangle satisfies

22 $${e}^{-i{{\Phi }}}	=\,{e}^{i{\phi }_{12}}{e}^{i{\phi }_{23}}{e}^{i{\phi }_{34}}{e}^{i{\phi }_{41}}\\ 	=\,{e}^{i{\phi }_{12}}{e}^{i{\phi }_{14}}{e}^{i{\phi }_{21}}{e}^{i{\phi }_{41}}{{\mathsf{G}}}_{a}(2){{\mathsf{G}}}_{a}^{*}(3){{\mathsf{G}}}_{b}^{*}(4){{\mathsf{G}}}_{b}(3)\\ 	=\,{{\mathsf{G}}}_{a}^{*}(3){{\mathsf{G}}}_{b}^{*}({L}_{a}^{-1}(3)){{\mathsf{G}}}_{a}({L}_{b}^{-1}(3)){{\mathsf{G}}}_{b}(3)={\sigma }^{*}.$$

Thus, in the presence of flux Φ, *L*_*a*_ and *L*_*b*_ satisfy ${{\mathsf{L}}}_{a}{{\mathsf{L}}}_{b}{{\mathsf{L}}}_{a}^{-1}{{\mathsf{L}}}_{b}^{-1}={e}^{i{{\Phi }}}$. This argument can be generalized to other lattices. See Supplementary Figs. 28 and 37.

(ii) For a cohomology invariant $\alpha={{\mathsf{R}}}_{2\pi /n}^{n}$, *n* must be even. Here, we have added the subscript 2*π*/*n* for R to specify the rotation angle. When *n* is even, rotating *n*/2 times is a twofold rotation ${R}_{2\pi /n}^{n/2}={R}_{\pi }$. In general, ${{\mathsf{R}}}_{2\pi /n}^{n/2}=\xi {{\mathsf{R}}}_{\pi }$ with $\xi \in {{\mathbb{Z}}}_{2}$. No matter whether *ξ* = ± 1, the cohomology invariant can always be expressed as

23 $${{\mathsf{R}}}_{\pi }^{2}=\alpha .$$

Substituting R_*π*_ = G_*π*_*R*_*π*_ into the identity above, we see the cohomology invariant can be realized by

24 $${{\mathsf{G}}}_{\pi }(i){{\mathsf{G}}}_{\pi }({R}_{\pi }(i))=\alpha$$

for any site *i*.

Let us label the vertices of the plaquette in Fig. 1d by *i* = 1, 2, 3, ⋯ 2*l* with *n* = 2*l*. Then, *R*_*π*_(*i*) = *i* + *l*. From (16), the hopping amplitudes satisfy

25 $${t}_{i,i+1}\,=\,{t}_{i+l,i+l+1}{{\mathsf{G}}}_{\pi }(i){{\mathsf{G}}}_{\pi }^{*}(i+1).$$

Then, the flux is found to be

26 $${e}^{-i{{\Phi }}}	=\mathop{\prod }\limits_{i=1}^{2l}{e}^{i{\phi }_{i,i+1}}=\mathop{\prod }\limits_{i=1}^{l}{{\mathsf{G}}}_{{r}_{\pi }}(i){{\mathsf{G}}}_{{r}_{\pi }}^{*}(i+1)\\ 	=\,{{\mathsf{G}}}_{{r}_{\pi }}(1){{\mathsf{G}}}_{{r}_{\pi }}^{*}(l+1)$$

From (24), we conclude that *e*^*i*Φ^ = *α*. Note that all phases are restricted in ${{\mathbb{Z}}}_{2}=\{\pm 1\}$.

(*iii*) For M^2^ = *β*, the 2D mirror reflection is interpreted as the twofold rotation through an axis parallel to the 2D plane. Then, it is clear from (*ii*) that *e*^*i*Φ^ = *β* with Φ the flux through each vertical plaquettes in Fig. 1e.

(*iv*) The cohomology invariant $\eta={{\mathsf{M}}}_{x}{{\mathsf{L}}}_{y}{{\mathsf{M}}}_{x}^{-1}{{\mathsf{L}}}_{y}^{-1}$ is translated as

27 $$\eta=	{{\mathsf{M}}}_{x}{{\mathsf{L}}}_{y}{{\mathsf{M}}}_{x}^{-1}{{\mathsf{L}}}_{y}^{-1}={{\mathsf{G}}}_{m}{M}_{x}{{\mathsf{G}}}_{y}{L}_{y}{({{\mathsf{G}}}_{m}{M}_{x})}^{-1}{({{\mathsf{G}}}_{y}{L}_{y})}^{-1}\\=	{{\mathsf{G}}}_{m}({M}_{x}{{\mathsf{G}}}_{y}{M}_{x}^{-1}){M}_{x}{L}_{y}{M}_{x}^{-1}{L}_{y}^{-1}({L}_{y}{{\mathsf{G}}}_{m}{L}_{y}^{-1}){{\mathsf{G}}}_{y}^{-1}.$$

Hence, we have the identity,

28 $${{\mathsf{G}}}_{m}(i){{\mathsf{G}}}_{y}({M}_{x}(i)){{\mathsf{G}}}_{m}^{*}({L}_{y}^{-1}(i)){{\mathsf{G}}}_{y}^{*}(i)=\eta .$$

Label the four white sites in Fig. 1f by 1, 2, 3 and 4, which are counted counterclockwise from the lower left site. From (16), we have the identities,

29 $${t}_{23}={t}_{14}{{\mathsf{G}}}_{m}(2){{\mathsf{G}}}_{m}^{*}(3),\quad {t}_{43}={t}_{12}{{\mathsf{G}}}_{y}(4){{\mathsf{G}}}_{y}^{*}(3),$$

Then, the flux is computed as

30 $${e}^{-i{{\Phi }}}=\,	{e}^{i{\phi }_{12}}{e}^{i{\phi }_{23}}{e}^{i{\phi }_{34}}{e}^{i{\phi }_{41}}\\=\,	{e}^{i{\phi }_{12}}{e}^{i{\phi }_{14}}{e}^{i{\phi }_{21}}{e}^{i{\phi }_{41}}{{\mathsf{G}}}_{m}(2){{\mathsf{G}}}_{m}^{*}(3){{\mathsf{G}}}_{y}^{*}(4){{\mathsf{G}}}_{y}(3)\\=\,	{{\mathsf{G}}}_{m}^{*}(3){{\mathsf{G}}}_{y}^{*}(M(3)){{\mathsf{G}}}_{m}({L}_{y}^{-1}(3)){{\mathsf{G}}}_{y}(3)={\eta }^{*}.$$

(*v*) The algebraic relation $\tau={{\mathsf{L}}}_{x}{{\mathsf{g}}}_{x}{{\mathsf{L}}}_{x}{{\mathsf{g}}}_{x}^{-1}$ leads to

31 $${{\mathsf{L}}}_{x}{{\mathsf{g}}}_{x}{{\mathsf{L}}}_{x}{{\mathsf{g}}}_{x}^{-1} 	={{\mathsf{G}}}_{x}{L}_{x}{{\mathsf{G}}}_{g}{g}_{x}({{\mathsf{G}}}_{x}{L}_{x}){({{\mathsf{G}}}_{g}{g}_{x})}^{-1}\\ 	={{\mathsf{G}}}_{x}({L}_{x}{{\mathsf{G}}}_{g}{L}_{x}^{-1}){L}_{x}{g}_{x}{L}_{x}{g}_{x}^{-1}({({L}_{x}{g}_{x})}^{-1}{{\mathsf{G}}}_{x}{L}_{x}{g}_{x}^{-1}){{\mathsf{G}}}_{g}^{-1},$$

which is equivalent to

32 $${{\mathsf{G}}}_{x}({{{{ r}}}}){{\mathsf{G}}}_{g}({L}_{x}({{{{ r}}}})){{\mathsf{G}}}_{x}({L}_{x}{g}^{-1}({{{{ r}}}})){{\mathsf{G}}}_{g}^{*}({{{{ r}}}})=\tau .$$

For the lattice in Fig. 1h, the hopping amplitudes satisfy

33 $${t}_{23}\,=\,{t}_{14}{{\mathsf{G}}}_{x}(2){{\mathsf{G}}}_{x}^{*}(3),\quad {t}_{43}={t}_{21}{{\mathsf{G}}}_{{g}_{x}}(4){{\mathsf{G}}}_{{g}_{x}}^{*}(3),$$

referring to (16). Here, 1, 2, 3, 4 label the four sites in Fig. 1h, which are counted counterclockwise from the lower left one. Then, the flux through the rectangular plaquette is

34 $${e}^{-i{{\Phi }}}	=\,{e}^{i{\phi }_{12}}{e}^{i{\phi }_{23}}{e}^{i{\phi }_{34}}{e}^{i{\phi }_{41}}\\ 	=\,{e}^{i{\phi }_{12}}{e}^{i{\phi }_{14}}{e}^{i{\phi }_{12}}{e}^{i{\phi }_{41}}{{\mathsf{G}}}_{x}(2){{\mathsf{G}}}_{x}^{*}(3){{\mathsf{G}}}_{{g}_{x}}^{*}(4){{\mathsf{G}}}_{{g}_{x}}(3)\\ 	=\,{e}^{i2{\phi }_{12}}{{\mathsf{G}}}_{x}^{*}(3){{\mathsf{G}}}_{{g}_{x}}^{*}({L}_{x}(3)){{\mathsf{G}}}_{x}({L}_{x}{g}_{x}^{-1}(3)){{\mathsf{G}}}_{{g}_{x}}(3)\\ 	=\,{{\mathsf{G}}}_{x}^{*}(3){{\mathsf{G}}}_{{g}_{x}}^{*}({L}_{x}(3)){{\mathsf{G}}}_{x}^{*}({L}_{x}{g}_{x}^{-1}(3)){{\mathsf{G}}}_{{g}_{x}}(3)={\tau }^{*}.$$

Note again that all phases above are either + 1 or − 1.

### Construction of canonical models

In the main text, we have elucidated how to realize cohomology invariants *σ*, *α*, *β*, *η* and *τ* in Table 1 by lattice flux configurations. Here, we briefly introduce the general procedure for constructing the canonical models for all 17 wallpaper groups, which realize all 458 PSAs. It must be noted that the purpose of these models is to demonstrate the physical realization of all PSAs, so they are made as simple as possible and contain only nearest neighbor hoppings. One can certainly write down more complex models with more complicated lattice structures and hopping processes for a given PSA, just like what one typically does when constructing models based on ordinary representations of space groups.

The illustration for all the canonical models is given in Fig. 3, and the full details for the model construction can be found in the Supplemental Note 3. In this process, we find it useful to categorize the 17 wallpaper groups into five classes.

(*a*) Groups *P*1, *P*3, and *Pg* are quite simple, since each of them has only one cohomology invariant *σ* or *τ* (see Table 1). It is straightforward to design lattice models with the flux patterns as introduced in (*i*) or (*iv*).

(*b*) For groups *P*2, *Pgg*, *P*4, and *P*6, all cohomology invariants are of type *α* in (*ii*), i.e., each *α*_*i*_ = R^*n*^ for some *n*-fold rotation R through a rotation center in the unit cell. Under lattice translations, each rotation center gives a lattice of rotation centers. Accordingly, each *α*_*i*_ is associated with such a lattice, and different *α*_*i*_^’s correspond to different lattices. This has been illustrated with our example *P*2 in the main text. Then, the canonical model is constructed as the dual lattice for the lattice of rotation centers. This means each plaquette in the canonical model hosts a unique rotation center; conversely, each rotation center is the center of a plaquette preserving the rotation symmetry. Then, each *α*_*i*_ = ± 1 is realized by inserting flux 0 or *π* into the corresponding plaquettes.

(*c*) For groups *Cm*, *P*3*m*1, and *P*31*m*, each has two cohomology invariants *σ* and *β*. We first construct a one-layer lattice model realizing *σ* as described in (*i*). Then, we double the one-layer model into a two-layer model, and introduce the nearest-neighbor interlayer hopping amplitudes to realize *β* as given in (*iii*) or in Fig. 1e.

(*d*) Each of groups *Pmm*, *Cmm*, *P*4*m*, *P*4*g*, and *P*6*m* has the two types of cohomology invariants *α* and *β* in (*ii*) and (*iii*), respectively. Here, following (*b*), we first construct a one-layer model to accommodate all *α*-invariants. Then, we double the one-layer model into a two-layer model, and appropriately insert fluxes for interlayer plaquettes to realize all *β*-invariants. Note that according to Fig. 1e, the vertical mirror planes should cross lattice bonds rather than lattice sites, which can always be satisfied.

(*e*) The remaining two groups are *Pm* and *Pmg*, both having *η*- and *β*-invariants. Therefore, we refer to (*iv*) and Fig. 1g for constructing two-layer models for them. Since *Pmg* also has two *α*-invariants, we first construct the one-layer model according to the *α*-invariants following (*b*), and then double it into a two-layer model to accommodate the *β*- and *η*-invariants following Fig. 1g.

### Shift of high-symmetry points

We derive the shift of high-symmetry points in Fig. 2c. From the PSAs for *P*2, it is straightforward to derive that

35 $${{\mathsf{L}}}_{a}{{\mathsf{L}}}_{b}{{\mathsf{L}}}_{a}^{-1}{{\mathsf{L}}}_{b}^{-1}={\alpha }_{1}{\alpha }_{2}{\alpha }_{3}{\alpha }_{4},\quad {{\mathsf{R}}}^{2}={\alpha }_{1},\quad {\mathsf{R}}{{\mathsf{L}}}_{b}{{\mathsf{R}}}^{-1}{{\mathsf{L}}}_{b}={\alpha }_{1}{\alpha }_{3},\quad {\mathsf{R}}{{\mathsf{L}}}_{a}{{\mathsf{R}}}^{-1}{{\mathsf{L}}}_{a}={\alpha }_{1}{\alpha }_{2}.$$

Alternatively, these relations can be derived from the configuration of the canonical model in Fig. 2a. There, the fluxes through the plaquettes colored in red, green, purple, blue correspond to cohomology invarants *α*_1_, *α*_2_, *α*_3_, *α*_4_, respectively. If *α*_*i*_ = 1(*α*_*i*_ = − 1), the corresponding flux is 0 (*π*).

When *α*_1_ = *α*_2_ = *α* and *α*_3_ = *α*_4_ = − *α*, plaquettes in each row have the same flux, and the flux values alternate across the rows (see Fig. 2b). Accordingly, the third equation above gives ${\mathsf{R}}{{\mathsf{L}}}_{b}{{\mathsf{R}}}^{-1}=-{{\mathsf{L}}}_{b}^{-1}$. In momentum space, L_*b*_ is diagonalized as *e*^*i****k***⋅*b*^. Then, ${\mathsf{R}}{e}^{i{{{{{{{\boldsymbol{k}}}}}}}}\cdot b}{{\mathsf{R}}}^{-1}={e}^{-i({{{{{{{\boldsymbol{k}}}}}}}}-{{{{{{{{\boldsymbol{G}}}}}}}}}_{b}/2)\cdot b}$, with ***G***_*b*_ the reciprocal lattice vector for *b*. Hence, we see that ***k*** is transformed to − ***k*** + ***G***_*b*_/2 under R, i.e.,

36 $${\mathsf{R}}:\,{{{{{{{\boldsymbol{k}}}}}}}}\mapsto -{{{{{{{\boldsymbol{k}}}}}}}}+{{{{{{{{\boldsymbol{G}}}}}}}}}_{b}/2.$$

Thus, the R-invariant momenta are shifted to ± ***G***_*b*_/4 and ***G***_*a*_/2 ± ***G***_*b*_/4, as shown in Fig. 2c.

### Enforced topology by projective symmetry algebras

Here, we provide the details for the nontrivial Zak phase enforced by R*T* symmetry discussed in the main text. The R*T* symmetry puts the following constraint for a Hamiltonian:

37 $${U}_{{\mathsf{R}}T}{{{{{{{{\mathcal{H}}}}}}}}}^{*}({{{{{{{\boldsymbol{k}}}}}}}}+{{{{{{{{\boldsymbol{G}}}}}}}}}_{b}/2){U}_{{\mathsf{R}}T}^{{{{\dagger}}} }={{{{{{{\mathcal{H}}}}}}}}({{{{{{{\boldsymbol{k}}}}}}}})$$

where *U*_R*T*_ is a unitary operator determined by R*T*. Suppose that ${{{{{{{\mathcal{H}}}}}}}}({{{{{{{\boldsymbol{k}}}}}}}})$ has a single band $| {\psi }_{{{{{{{{\boldsymbol{k}}}}}}}}}\rangle$ over the ***G***_*b*_ period from ***k*** = 0 to ***G***_*b*_. The action of R*T* on $| {\psi }_{{{{{{{{\boldsymbol{k}}}}}}}}}\rangle$ will give a band eigenstate at ***k*** + ***G***_*b*_/2, generally differing from $| {\psi }_{{{{{{{{\boldsymbol{k}}}}}}}}+{{{{{{{{\boldsymbol{G}}}}}}}}}_{b}/2}\rangle$ by a ***k*** dependent phase, i.e.,

38 $${U}_{{\mathsf{R}}T}{ | {\psi }_{{{{{{{{\boldsymbol{k}}}}}}}}}\rangle }^{*}={e}^{i\phi ({{{{{{{\boldsymbol{k}}}}}}}})} | {\psi }_{{{{{{{{\boldsymbol{k}}}}}}}}+{{{{{{{{\boldsymbol{G}}}}}}}}}_{b}/2}\rangle .$$

Accordingly, R*T* relates the Berry connection ${{{{{{{{\mathcal{A}}}}}}}}}_{b}({{{{{{{\boldsymbol{k}}}}}}}})=\left\langle {\psi }_{{{{{{{{\boldsymbol{k}}}}}}}}}\right|i{\partial }_{{k}_{b}}\left | {\psi }_{{{{{{{{\boldsymbol{k}}}}}}}}}\right\rangle$ at ***k*** and ***k*** + ***G***_*b*_/2 as

39 $${{{{{{{{\mathcal{A}}}}}}}}}_{b}({{{{{{{\boldsymbol{k}}}}}}}})+{{{{{{{{\mathcal{A}}}}}}}}}_{b}({{{{{{{\boldsymbol{k}}}}}}}}+{{{{{{{{\boldsymbol{G}}}}}}}}}_{b}/2)={\partial }_{{k}_{b}}\phi ({{{{{{{\boldsymbol{k}}}}}}}}).$$

Because of this, the Zak phase ${\theta }_{b}=\oint d{k}_{b}\,{{{{{{{{\mathcal{A}}}}}}}}}_{b}({{{{{{{\boldsymbol{k}}}}}}}})$ over any ***G***_*b*_-periodic path can be expressed as

40 $${\theta }_{b}=\phi ({{{{{{{{\boldsymbol{G}}}}}}}}}_{b}/2)-\phi (0).$$

Moreover, the PSA relation (R*T*)^2^ = *α* requires that ${U}_{{\mathsf{R}}T}{U}_{{\mathsf{R}}T}^{*}=\alpha$, which in turn leads to

41 $${e}^{i\phi ({{{{{{{\boldsymbol{k}}}}}}}}+{{{{{{{{\boldsymbol{G}}}}}}}}}_{b}/2)-i\phi ({{{{{{{\boldsymbol{k}}}}}}}})}=\alpha .$$

Thus, we arrive at ${\theta }_{b}=i\ln \alpha \,{{{{{{\mathrm{mod}}}}}}}\,\,2\pi$ as claimed in the main text. For *α* = − 1, this Zak phase is guaranteed to be nontrivial.

### Eightfold degenerate nodal point

For the group *P*3*m*1, when *σ* = *α* = − 1, the little co-algebra at the Γ point has two irreducible 4D representations and one irreducible 8D representation. The 8D irreducible representation can be expressed as

42 $${{\mathsf{L}}}_{a}	=i{\sigma }_{1}\otimes {\sigma }_{0}\otimes {\sigma }_{0},\quad {{\mathsf{L}}}_{b}=i{\sigma }_{3}\otimes {\sigma }_{0}\otimes {\sigma }_{0},\quad {\mathsf{R}}={U}_{R}\otimes {D}_{R}\otimes {\sigma }_{0},\quad \\ {\mathsf{M}}	={U}_{M}\otimes {D}_{M}\otimes {\sigma }_{3},\quad T={\sigma }_{2}\otimes {\sigma }_{2}\otimes {\sigma }_{1}K.$$

Here, *K* denotes the complex conjugation, and

43 $${U}_{R}	=\exp (i{{{{{{{{\boldsymbol{n}}}}}}}}}_{1}\cdot {{{{{{{\boldsymbol{\sigma }}}}}}}}2\pi /3),\quad {D}_{R}=\exp (-i{\sigma }_{2}2\pi /3),\quad \\ {U}_{M}	=\exp (i{{{{{{{{\boldsymbol{n}}}}}}}}}_{2}\cdot {{{{{{{\boldsymbol{\sigma }}}}}}}}\pi /2),\quad {D}_{M}={\sigma }_{3},$$

with $${{{{{{{{\boldsymbol{n}}}}}}}}}_{2}=(1,-1,-1)/\sqrt{3}$$ and $${{{{{{{{\boldsymbol{n}}}}}}}}}_{2}=(0,1,-1)/\sqrt{2}$$.

Following (*c*), we can construct the canonical model for *P*3*m*1. First, we construct the one-layer lattice that realizes the *σ*-invariant for translations as illustrated in Fig. 2d. Then, we double the one-layer model into the bilayer model as illustrated in Fig. 2e. In order to realize all cohomology invariants, we add flux at all regular hexagons and rectangles. The expression for this lattice model is given in the Supplementary Note 4.

## Supplementary information

Supplementary information

## Data Availability

The data generated and analyzed during this study are available from the corresponding author upon request.

## Code availability

All code used to generate the plotted band structures is available from the corresponding author upon request.
