# Supplementary material for: Classification of time-reversal-invariant crystals with gauge structures
Source: Nat Commun. 2023 Feb 10;14:743. doi: 10.1038/s41467-023-36447-7 (PMC9918504; doi:10.1038/s41467-023-36447-7)
Supplement: Supplementary file 1 — Supplementary information [file 41467_2023_36447_MOESM1_ESM.pdf]

Supplementary information for
"Classification of time-reversal-invariant crystals with gauge structures"

Z. Y. Chen,[1] Zheng Zhang,[1] Shengyuan A. Yang,[2] and Y. X. Zhao[1,3,*]

[1] *National Laboratory of Solid State Microstructures and Department of Physics, Nanjing University, Nanjing 210093, China*
[2] *Research Laboratory for Quantum Materials, Singapore University of Technology and Design, Singapore 487372, Singapore*
[3] *Collaborative Innovation Center of Advanced Microstructures, Nanjing University, Nanjing 210093, China*

Contents

Supplementary Note 1. Background 1

Supplementary Note 2. Projective symmetry algebras of wallpaper groups and their cohomology invariants 6

Supplementary Note 3. The construction of the canonical models from the cohomology invariants 28

Supplementary Note 4. Other technical details for results in the main text 42

Supplementary Note 5. Engineering gauge fluxes in artificial crystals 49

Supplementary Note 1. Background

In this section, we introduce the basics of projective symmetry algebras for a given symmetry group G. First, we introduce the multipliers or factor systems of projective representations, which are classified by the second cohomology group $H^2(G, U(1))$. Then, we discuss the consequences of including time-reversal symmetry into the symmetry group. Finally, we establish the correspondence between projective symmetry algebras and cohomology invariants.

a. Introduction to projective representation

A projective representation of a group G with coefficients in an Abelian group $\mathcal{A}$ is a map from G to linear transfomations on a vector space V:

$$\rho : G \to \mathrm{GL}(V), \tag{1}$$

which satisfies a modified multiplication rule

$$\rho(g_1)\rho(g_2) = \nu(g_1, g_2)\rho(g_1g_2), \quad \forall g_1, g_2 \in G. \tag{2}$$

Here, $\nu(g_1, g_2)$ is a function from $G \times G$ to $\mathcal{A}$, which is called a factor system or multiplier. The associativity requires that

$$\nu(g_1, g_2)\nu(g_1g_2, g_3) = \nu(g_2, g_3)\nu(g_1, g_2g_3). \tag{3}$$

A function from $G \times G$ to $\mathcal{A}$ satisfies Eq. (3) is called a 2-cocyle. We denote the set of all possible 2-cocyles as $Z^2(G, \mathcal{A})$, which is an abelian group under the multiplication of functions.

$\rho(g)$ can be redefined by a phase factor $\chi(g) \in \mathcal{A}$: $\rho(g) \to \rho'(g) = \chi(g)\rho(g)$, with which the factor system ν is transformed as

$$\nu(g_1, g_2) \to \nu'(g_1, g_2) = \nu(g_1, g_2)\frac{\chi(g_1)\chi(g_2)}{\chi(g_1g_2)}. \tag{4}$$

---

* zhaoyx@nju.edu.cn

We regard ν and ν' as equivalent factor systems. They differs each other by a trivial 2-cocyle $\frac{\chi(g_1)\chi(g_2)}{\chi(g_1g_2)}$. All trivial 2-cocyles form an abelian group $B^2(G,\mathcal{A}) \subset Z^2(G,\mathcal{A})$. Thus, the non-equivalent classes of factor systems are given by the quotient group

$$H^2(G,\mathcal{A}) = Z^2(G,\mathcal{A})/B^2(G,\mathcal{A}), \tag{5}$$

which is also called a second cohomology group.

A projective representation of G with multiplier $\nu \in \mathcal{A}$ corresponds to a group extension of G by $\mathcal{A}$:

$$1 \xrightarrow{i} \mathcal{A} \to \tilde{G} \xrightarrow{p} G \to 1. \tag{6}$$

Here, i is an injective homomorphism, i.e., $\mathcal{A}$ is a subgroup of $\tilde{G}$, and p is a surjective homomorphism. We now consider a lift from G to $\tilde{G}$, i.e., for each $g \in G$ we assign $\mathsf{g} \in \tilde{G}$ with $p(\mathsf{g}) = g$. Then, $g_1g_2 = g_3$ is lifted to $\mathsf{g}_1\mathsf{g}_2 = \tilde{\nu}(g_1,g_2)\mathsf{g}_3$ with $\tilde{\nu}(g_1,g_2) \in \mathcal{A}$. It can be shown that $\tilde{\nu}$ is a 2-cocycle, and ν and $\tilde{\nu}$ are in the same cohomology class.

b. Projective symmetry algebras with time reversal

Time reversal T generates a twofold cyclic group, which we denote as $\mathcal{Z}_2^T$. In our work, we consider symmetry groups in the form of $G \times \mathcal{Z}_2^T$, where G is a spatial symmetry group.

The equivalence classes of multipliers form an abelian group, termed as the twisted second-cohomology group

$$H^{c,2}(G \times \mathcal{Z}_2^T, U(1)), \tag{7}$$

where the superscript c indicates the complex conjugation of T on $U(1)$. Below, we show that each multiplier λ of $G \times \mathcal{Z}_2^T$ is similar to the following decomposition

$$\lambda(g_1T^{a_1}, g_2T^{a_2}) = \nu(g_1,g_2)\omega(T^{a_1},T^{a_2}), \quad \nu,\omega \in \mathbb{Z}_2 = \{\pm 1\}, \tag{8}$$

where $g_1,g_2 \in G$ and $a_1,a_2 \in \{0,1\}$. This decomposition implies that

$$H^{c,2}(G \times \mathcal{Z}_2^T, U(1)) = H^2(G,\mathbb{Z}_2) \times H^2(\mathcal{Z}_2^T,\mathbb{Z}_2). \tag{9}$$

Before proving this result, let us first give some remarks on its physical meanings.

(i) With time-reversal invariance, the multipliers can be restricted to be valued in $\mathbb{Z}_2$, rather than in $U(1)$. Conversely, it is sufficient to consider the second group cohomology with $\mathbb{Z}_2$ as coefficient. Since the time-reversal operator acts trivially on $\mathbb{Z}_2 \subset U(1)$, the twisted cohomology is "untwisted". This is particularly interesting in quantum physics. For instance, recall that according to Wigner each multiplier of the inhomogeneous Lorentz group (the connected component containing the identity) is similar to one valued in $\mathbb{Z}_2$. Then, all multipliers of the inhomogeneous Lorentz groups can preserve time-reversal symmetry. This seems an accidental nice property, given that time-reversal is not included in the inhomogeneous Lorentz group. Wigner's result can be traced back to the fundamental group of the inhomogeneous Lorentz group, which is $\mathbb{Z}_2$. Analogous analysis can be given for $SO(3)$.

(ii) $H^2(\mathcal{Z}_2^T,\mathbb{Z}_2) \cong \mathbb{Z}_2$ is specified by $\mathcal{T}^2 = (-1)^j$. Here, $j\hbar/2$ is the spin number of the particles under consideration, and $\mathcal{T}$ denotes the anti-unitary operator representing T.

(iii) The classification of projective representations of $G \times \mathcal{Z}_2^T$, namely $H^{c,2}(G \times \mathcal{Z}_2^T, U(1))$, has been reduced to calculating $H^2(G,\mathbb{Z}_2)$ with coefficient $\mathbb{Z}_2$.

(iv) That the spin is integral or half integral specifies a $\mathbb{Z}_2$-multiplier of $G \times \mathcal{Z}_2^T$, which we denote by λ_s with $s = 0, 1/2$ for integral and half integral spins, respectively. Restricted on G, let us denote $\nu_s = \lambda_s|_{G\times G}$.

(v) If G consists of spatial transformations over a lattice, the $\mathbb{Z}_2$ gauge flux configuration on the lattice can endow a $\mathbb{Z}_2$-multiplier ν_f for G, and can preserve time-reversal symmetry at the same time.

(vi) Still consider G consisting of spatial transformations over a lattice. Then, the internal spin degrees of freedom and the external gauge fluxes together lead to the $\mathbb{Z}_2$-multiplier for G:

$$\nu(g_1,g_2) = \nu_s(g_1,g_2)\nu_f(g_1,g_2). \tag{10}$$

(vii) It is possible that some $\mathbb{Z}_2$ gauge flux configurations F can realize $\nu_{s=1/2}$. Then, such as flux configuration can exchange the multiplier classes of integral and half integral spins.

We now proceed to prove every $U(1)$-multiplier σ is similar to the decomposition into two $\mathbb{Z}_2$-multipliers: $\lambda(g_1T^{a_1}, g_2T^{a_2}) = \nu(g_1, g_2)\omega(T^{a_1}, T^{a_2})$. We observe that

$$\rho(g)\rho(T) = \lambda(g,T)\rho(gT) = \frac{\lambda(g,T)}{\lambda(T,g)}\rho(T)\rho(g), \tag{11}$$

which motivates us to perform the transformation,

$$\tilde{\rho}(g) := \sqrt{\frac{\lambda(T,g)}{\lambda(g,T)}}\rho(g), \tag{12}$$

for all $g \in G$. Then, since $\rho(T)c = c^*\rho(T)$ for all $c \in \mathbb{C}$,

$$\tilde{\rho}(g)\rho(T) = \rho(T)\tilde{\rho}(g). \tag{13}$$

We further transform the operators for the other half of group elements as

$$\tilde{\rho}(gT) := \sqrt{\lambda(g,T)\lambda(T,g)}\rho(gT), \tag{14}$$

for all $g \in G$. Note that $\tilde{\rho}(T) = \rho(T)$. Hence,

$$\tilde{\rho}(g)\tilde{\rho}(T) = \tilde{\rho}(gT). \tag{15}$$

We further restrict the transformed multiplier $\tilde{\lambda}$ to G. Considering

$$\tilde{\rho}(g_1)\tilde{\rho}(g_2) = \tilde{\lambda}(g_1, g_2)\tilde{\rho}(g_1g_2) \tag{16}$$

we see the left side commutes with $\tilde{\rho}(T)$, so does the right side. Hence, $\nu = \tilde{\lambda}|_{G\times G} \in \mathbb{Z}_2 = \{\pm 1\}$. Restricting $\tilde{\lambda}$ on $\mathcal{Z}_2^T$, there is only one variable $\tilde{\lambda}(T,T)$, which appears in

$$\tilde{\rho}(T)\tilde{\rho}(T) = \tilde{\lambda}(T,T)1. \tag{17}$$

Clearly, $\tilde{\lambda}(T,T)$ commutes with $\tilde{\rho}(T)$, and therefore $\omega = \tilde{\lambda}|_{\mathcal{Z}_2^T\times\mathcal{Z}_2^T} \in \mathbb{Z}_2$.

Finally, it is straightforward to check

$$\begin{aligned}\tilde{\rho}(g_1T^{a_1})\tilde{\rho}(g_2T^{a_2}) &= \tilde{\rho}(g_1)\tilde{\rho}(T^{a_1})\tilde{\rho}(g_2)\tilde{\rho}(T^{a_2})\\ &= \tilde{\rho}(g_1)\tilde{\rho}(g_2)\tilde{\rho}(T^{a_1})\tilde{\rho}(T^{a_2})\\ &= \nu(g_1,g_2)\tilde{\rho}(g_1g_2)\omega(T^{a_1},T^{a_2})\tilde{\rho}(T^{a_1+a_2})\\ &= \nu(g_1,g_2)\omega(T^{a_1},T^{a_2})\tilde{\rho}(g_1g_2T^{a_1+a_2}).\end{aligned} \tag{18}$$

which verifies the decomposition form. Note that we have repeatedly used the facts: $\tilde{\rho}(g)\tilde{\rho}(T) = \tilde{\rho}(gT)$ and $\tilde{\rho}(g)\tilde{\rho}(T) = \tilde{\rho}(T)\tilde{\rho}(g)$.

c. Projective symmetry algebras and cohomology invariants

Let us now discuss how to present all cohomology classes of multipliers for a symmetry group G in terms of cohomology invariants constructed as algebraic relations of symmetry operators.

Mathematically, a wallpaper group can be presented by generators and relations (the algebra of generators)

$$G = \langle \mathcal{S}|\mathcal{R}\rangle, \tag{19}$$

where $\mathcal{S}$ is the set of generators $\{s_1, s_2, \cdots, s_{n_s}\} \subset G$, and $\mathcal{R}$ is the set of relations among generators $\{r_1, r_2, \cdots, r_{n_r}\}$, which satisfy

$$r_1(\boldsymbol{s}) = 1, r_2(\boldsymbol{s}) = 1, \cdots, r_{n_r}(\boldsymbol{s}) = 1. \tag{20}$$

For any element $g \in G$, we can specify a "word" of generators to form it,

$$w(g) = s_{i_1} s_{i_2} \cdots . \tag{21}$$

Two "words" can be equal under the relations, for example,

$$\begin{aligned} w_i &= s_{i_1} s_{i_2} \cdots s_{i_{m-1}} \underbrace{s_{i_m} \cdots s_{i_{m+k-1}}}_{r_j=1} s_{i_{m+k}} \cdots \\ &= s_{i_1} s_{i_2} \cdots s_{i_{m-1}} s_{i_{m+k}} \cdots = w_f. \end{aligned} \tag{22}$$

The projective representation of a wallpaper group can be described in a more compact way by modifying relations of the presentation:

$$r_1(\boldsymbol{s}) = \alpha_1, r_2(\boldsymbol{s}) = \alpha_2, \cdots, r_{n_r}(\boldsymbol{s}) = \alpha_{n_r}, \tag{23}$$

where $\alpha_1, \alpha_2, \cdots, \alpha_{n_r} \in \mathcal{A}$. We denote the modified presentation as

$$\tilde{G} = \langle \mathcal{S} | \mathcal{R}, \mathcal{F} \rangle, \tag{24}$$

where $\mathcal{F} : \mathcal{R} \to \mathcal{A}$ is a function describing the modification, $\mathcal{F}(r_i) = \alpha_i$. We call the modified presentation $\tilde{G}$ a *projective symmetry algebra*, or shortly, *projective algebra*. It can be considered as a modified group algebra over $\mathcal{A}$.

Now, two"words" can be equal up to a phase under the new relations, for example,

$$\begin{aligned} w_i &= s_{i_1} s_{i_2} \cdots s_{i_{m-1}} \underbrace{s_{i_m} \cdots s_{i_{m+k-1}}}_{r_j=\alpha_j} s_{i_{m+k}} \cdots \\ &= \alpha_j s_{i_1} s_{i_2} \cdots s_{i_{m-1}} s_{i_{m+k}} \cdots = \alpha_j w_f. \end{aligned} \tag{25}$$

Now, we show that all the cohomology classes of factor systems can be described in the form of Eq. (24).

Suppose we have a group G, which has a presentation $G = \langle \mathcal{S} | \mathcal{R} \rangle$. Given a projective representation of G, $\rho : G \to \mathrm{GL}(V)$, the representation of generators $\rho(s), s \in \mathcal{S}$ will satisfy modified relations

$$r_1(\rho(\boldsymbol{s})) = \alpha_1, r_2(\rho(\boldsymbol{s})) = \alpha_2, \cdots, r_{n_r}(\rho(\boldsymbol{s})) = \alpha_{n_r}. \tag{26}$$

where α_i is determined by the factor system. So the representation of generators form a modified presentation $\tilde{G} = \langle \rho(\mathcal{S}) | \mathcal{R}, \mathcal{F} \rangle$, where $\rho(\mathcal{S}) = \{\rho(s) | s \in \mathcal{S}\}$. In general, $\rho(\mathcal{S})$ does not generate the original projective representation, since for each group element $w(g) = s_{i_1} s_{i_2} \cdots$, the representation $\rho(g)$ in general has the form

$$\rho(g) = \chi_{\mathcal{S}}(g) \rho(s_{i_1}) \rho(s_{i_2}) \cdots , \tag{27}$$

where $\chi_{\mathcal{S}}(g)$ is a phase due to the factor system. However, we can redefining $\rho'(g) = \chi_{\mathcal{S}}(g)^{-1} \rho(g)$, which is exactly the projective representation generated by $\rho(\mathcal{S})$. Moreover, its factor system is in the same cohomology class with ρ. Thus, every cohomology classes of factor systems of G can be described by a projective algebra $\tilde{G}$. So, to discuss cohomology classes of factor systems, we can concertrate on projective algebras, which are much more simpler than factor systems.

Similar to the factor system ν, we cannot choose the factor function $\mathcal{F} \in \mathcal{A}^{n_r}$ arbitrarily because the modified relations may not be consistent with the associativity of $\tilde{G}$. If we start with a "word" w_i, associate generators in two different paths P_1, P_2 and result in the same final"word" w_f, the associativity requires

$$w_i = \left(\prod_{j \in P_1} \mathcal{F}(r_j) \right) w_f = \left(\prod_{j \in P_2} \mathcal{F}(r_j) \right) w_f. \tag{28}$$

This is a general requirement of associativity for projective algebra, and we will see that the cocyle equation Eq. (3) is a special form of it in the following. The set of factor functions $\mathcal{F}$ satisfy the associative condition Eq. (28) form a group $\mathrm{AMap}(\mathcal{R}, \mathcal{A}) \subset \mathcal{A}^{n_r}$. Furthermore, we can also redefine each generator $s_i \to s'_i = \chi_i s_i$, and values of factor function will be transformed as

$$\mathcal{F}(r_i(\boldsymbol{s})) \to \mathcal{F}'(r_i(\boldsymbol{s})) = \mathcal{F}(r_i(\boldsymbol{s})) r_i^{-1}(\boldsymbol{\chi}). \tag{29}$$

We call this transformation *coboundary transformation*. The sets of factor functions given by $r_i(\boldsymbol{\chi})$ are considered as trivial factor functions, whose set we denote as $\mathrm{TMap}(\mathcal{R}, \mathcal{A}) = \{\mathcal{F} : \mathcal{R} \to \mathcal{A} | \mathcal{F}(r_i(\boldsymbol{s})) = r_i(\boldsymbol{\chi}), \chi \in \{\mathcal{S} \to \mathcal{A}\}\} \subset$

AMap$(\mathcal{R},\mathcal{A})$. The quotient group of AMap$(\mathcal{R},\mathcal{A})$ by TMap$(\mathcal{R},\mathcal{A})$ will give the same result as the second group cohomology

$$\mathrm{AMap}(\mathcal{R},\mathcal{A})/\mathrm{TMap}(\mathcal{R},\mathcal{A}) = H^2(G,\mathcal{A}). \tag{30}$$

Here is an example. Every group has a trivial presentation $G = \langle \mathcal{S}|\mathcal{R}\rangle$, where $\mathcal{S}$ is the group G itself and $\mathcal{R} = \left\{r_{ij} = g_i g_j g_{ij}^{-1} = 1 | i,j \in G\right\}$ is the set of multiplication relations of G. When we consider the projective algebra over $\mathcal{A}$, The factor function is just the factor system $\mathcal{F}(r_{ij}) = \nu(g_i,g_j) = \alpha_{i,j} \in \mathcal{A}$. For a word of length three $g_1g_2g_3$, we have two paths to associate it into g_{123}

$$\begin{aligned} & g_1g_2g_3 \\ = & \underbrace{g_1g_2}_{\alpha_{1,2}g_{12}} g_3 = \alpha_{1,2} \underbrace{g_{12}g_3}_{\alpha_{12,3}g_{123}} = \alpha_{1,2}\alpha_{12,3}\, g_{123} \\ = & g_1 \underbrace{g_2g_3}_{\alpha_{2,3}g_{23}} = \alpha_{2,3} \underbrace{g_1g_{23}}_{\alpha_{1,23}g_{123}} = \alpha_{2,3}\alpha_{1,23}\, g_{123}. \end{aligned}$$

We see the cocyle equation of factor system Eq. (3) is derived as expectated. In this example, AMap$(\mathcal{R},\mathcal{A}) = Z^2(G,\mathcal{A})$, and trivial maps take the form as $r_{ij}(\boldsymbol{\chi}) = (\chi_i\chi_j)/\chi_{ij}$, thus TMap$(\mathcal{R},\mathcal{A}) = B^2(G,\mathcal{A})$.

For another example, we take a look at the group $G = \mathbb{Z}_2^2$, the presentation is given by

$$\mathbb{Z}_2^2 = \langle e_1, e_2 | e_1^2, e_2^2, e_1e_2e_1^{-1}e_2^{-1}\rangle. \tag{31}$$

When we consider the projective algebra over $U(1)$, the modified relations are

$$e_1^2 = \alpha_1, e_2^2 = \alpha_2, e_1e_2e_1^{-1}e_2^{-1} = \alpha_3, \quad \alpha_1,\alpha_2,\alpha_3 \in U(1). \tag{32}$$

For $w = e_1e_2e_1^{-1}e_2^{-1}e_2e_1e_2e_1^{-1}e_2^{-1}e_2$, we have two paths to associate the generators:

$$\begin{aligned} & w = e_1e_2e_1^{-1} \underbrace{e_2^{-1}e_2}_{1} e_1e_2e_1^{-1} \underbrace{e_2^{-1}e_2}_{1} = \\ & e_1e_2 \underbrace{e_1^{-1}e_1}_{1} e_2e_1^{-1} = e_1 \underbrace{e_2e_2}_{\alpha_2} e_1^{-1} = \alpha_2 \underbrace{e_1e_1^{-1}}_{1} = \alpha_2, \end{aligned} \tag{33}$$

and

$$\begin{aligned} & w = \underbrace{e_1e_2e_1^{-1}e_2^{-1}}_{\alpha_3} e_2 \underbrace{e_1e_2e_1^{-1}e_2^{-1}}_{\alpha_3} e_2 = \\ & \alpha_3^2 \underbrace{e_2e_2}_{\alpha_2} = \alpha_2\alpha_3^2. \end{aligned} \tag{34}$$

Thus, the associativity requires $\alpha_2\alpha_3^2 = \alpha_2$, i.e., α_3 can only takes value in $\{\pm 1\} \subset U(1)$. Since there are no other restriction on α_1 and α_2, AMap$(\mathcal{R},U(1)) = U(1)\otimes U(1)\otimes \mathbb{Z}_2$. By redefining $e_1 \to e_1' = e_1\chi_1, e_2 \to e_2' = e_2\chi_2$, factors $\alpha_1,\alpha_2,\alpha_3$ will be transformed as $\alpha_1 \to \alpha_1' = \alpha_1\chi_1^2$, $\alpha_2 \to \alpha_2' = \alpha_2\chi_2^2$. Thus $U(1)\otimes U(1) \subset$ AMap$(\mathcal{R},U(1))$ can be trivialized. Finally, the corresponding second group cohomology is $H^2(\mathbb{Z}_2^2, U(1)) = \mathbb{Z}_2$.

For a general group presentation $G = \langle \mathcal{S}|\mathcal{R}\rangle$, we do not find general form of self-consistent equations required by Eq. (28). However, for a wallpaper group G, there exist a standard presentation

$$w(g) = L_a^i L_b^j R^k M^l, \forall g \in G, \tag{35}$$

where L_a, L_b are generators of translations, and R, M are generators of rotation and reflection. For the production of two group elements $g_1g_2 = g_3$, we can always use relations to turn $w(g_1)w(g_2)$ into the standard presentation with an additional factor $\nu(g_1,g_2)$,

$$w(g_1)w(g_2) \quad = L_a^{i_1}L_b^{j_1}R^{k_1}M^{l_1}L_a^{i_2}L_b^{j_2}R^{k_2}M^{l_2} = \nu(g_1,g_2)L_a^{i_3}L_b^{j_3}R^{k_3}M^{l_3} = \nu(g_1,g_2)w(g_3). \tag{36}$$

With the factor system $\nu(g_1,g_2)$ obtained from the presentation, all the self-consistent equations can be found from cocyle equations of ν.

Solving cocyle equations is a tedious work, fortunately, the second group cohomology $H^2(G, U(1))$ and $H^2(G, \mathbb{Z}_2)$ are already known for all wallpaper groups. Thus, in this work, we use some self-consistent equations to reduce factors $\alpha_i = \mathcal{F}(r_i)$ and check the consistency with group cohomology.

In general, values of $\alpha_i = \mathcal{F}(r_i)$ is not invariant under coboundary transformation $\mathcal{F}(r_i) \to \mathcal{F}(r_i) r_i(\boldsymbol{\chi}), \forall \chi \in \{\mathcal{S} \to \mathcal{A}\}$. We can recombine original relations into new one $r'_i = r_{i_1} r_{i_2} \cdots$, whose value is $\mathcal{F}(r'_i) := \mathcal{F}(r_{i_1}) \mathcal{F}(r_{i_1}) \cdots$. If the new value is invariant under coboundary transformation $\mathcal{F}(r'_i) = \mathcal{F}(r'_i) r'_i(\boldsymbol{\chi}), \forall \chi \in \{\mathcal{S} \to \mathcal{A}\}$, we call it a *cohomology invariant*. Different classes of projective algebras of a wallpaper group can be labelled by a complete set of cohomology invariants, and thus all the factor systems of the wallpaper group can be obtained by enumerating all the possible values of cohomology invariants in this complete set. In the following, we will use projective algebras and cohomology invariants to describe factor systems of wallpaper groups.

Supplementary Note 2. Projective symmetry algebras of wallpaper groups and their cohomology invariants

Based on the basics of projective symmetry algebras introduced in the previous section, we proceed to construct all the ($U(1)$ and $\mathbb{Z}_2$) projective algebras and the corresponding cohomology invariants for the 17 wallpaper groups.

Let us start with summarizing our notations. Wallpaper groups contain the following primary symmetry operations: translation, rotation, reflection and glide-reflection. In general, L, R, M and g are used to represent them respectively. The modified symmetry operations are written in the bold font like $\mathsf{L}, \mathsf{R}, \mathsf{M}, \mathsf{g}$. We will use diagrams to visualize the wallpaper groups. In these diagrams, the rotation centers, reflection axes and glide-reflection axes are represented by the shapes in Fig. 1.

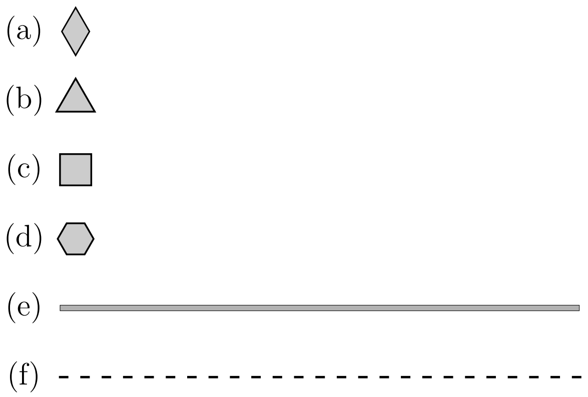

Supplementary Fig.1: Notations for cell structure. (a) A center of rotation of order two (π). (b) A center of rotation of order three ($2\pi/3$). (c) A center of rotation of order four ($\pi/2$). (d) A center of six-fold rotation ($\pi/3$). (d) An axis of reflection. (d) An axis of glide-reflection.

a. C_n and D_n

Before introducing projective algebras of wallpaper groups, we first introduce projective algebras of point group C_n and D_n, which can help the reader understand our method.

i. C_n

The group C_n is generated by rotation operation R with relation $R^n = 1$. The relation acquires a factor α after extension,

$$\mathsf{R}^n = \alpha. \tag{37}$$

For $U(1)$ extension, $\alpha \in U(1)$. However, this factor can be cancelled by redefining the generator $\mathsf{R} \to \mathsf{R}'$:

$$\mathsf{R}' = \alpha^{-1/n} \mathsf{R}, \quad (\mathsf{R}')^n = \alpha(\alpha^{-1/n})^n = 1. \tag{38}$$

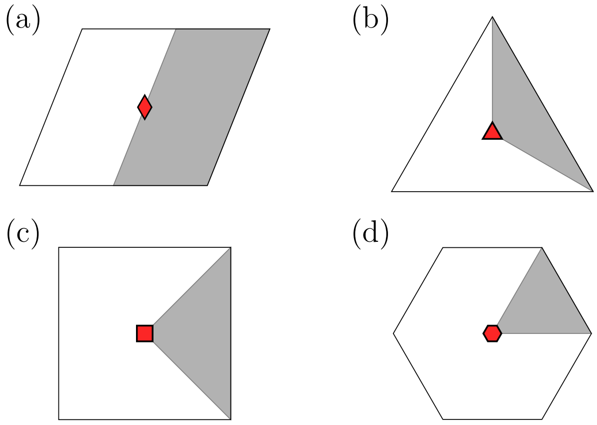

Supplementary Fig.2: Illustration of point groups C_n. (a) A parallelogram under point group C_2, the shaded region is the fundamental domain. (b) A triangle under point group C_3, the shaded region is the fundamental domain. (c) A square under point group C_4, the shaded region is the fundamental domain. (d) A hexagon under point group C_6, the shaded region is the fundamental domain.

So C_n only has the trivial $U(1)$ projective algebra, which is in agreement with the result of the group cohomology

$$H^2(C_n, U(1)) = 1. \tag{39}$$

For $\mathbb{Z}_2$ extension, $\alpha \in \{\pm 1\}$. When n is odd, we can redefine $\mathsf{R} \to \mathsf{R}' = \alpha \mathsf{R}$, thus $(\mathsf{R}')^n = \alpha\alpha^n = \alpha^{n+1} = 1$. So C_n only has the trivial $\mathbb{Z}_2$ projective algebra when n is odd. However, when n is even, factor α cannot be cancelled by redefining the generator R. So there are two nonequivalent projective algebras of C_n when n is even, where $\alpha = -1$ corresponds to the nontrivial projective representation and $\alpha = 1$ corresponds to the trivial one. One can check α is a cohomology invariant. These results are consistent with the results of the group cohomology

$$H^2(C_n, \mathbb{Z}_2) = \begin{cases} 1 & n \text{ odd} \\ \mathbb{Z}_2 & n \text{ even} \end{cases}. \tag{40}$$

ii. D_n

Now we proceed to analyze group D_n. The group D_n is generated by rotation operation R and mirror reflection operation M with relations $R^n = 1, M^2 = 1, MRM^{-1} = R^{-1}$. After extension, these relations become

$$\mathsf{R}^n = \alpha_r, \tag{41a}$$
$$\mathsf{M}^2 = \alpha_m, \tag{41b}$$
$$\mathsf{MRM}^{-1} = \alpha_{rm}\mathsf{R}^{-1}. \tag{41c}$$

To see the restriction on factors by the self-consistency condition, we do conjugate transformation for Eq. (41a), and we have

$$\begin{aligned} \alpha_r &= \mathsf{MR}^n\mathsf{M}^{-1} = (\mathsf{MRM}^{-1})^n \\ = \quad & \alpha_{rm}^n \mathsf{R}^{-n} = \alpha_{rm}^n \alpha_r^{-1}, \end{aligned} \tag{42}$$

where the associativity Eq. (28) is implied in the derivation. α_r and α_{rm} satisfy

$$\alpha_{rm}^n \alpha_r^{-2} = 1. \tag{43}$$

For $U(1)$ extension there are two solution of α_r in terms of α_{rm},

$$\alpha_r = \pm\alpha_{rm}^{n/2} \equiv \alpha\alpha_{rm}^{n/2}. \tag{44}$$

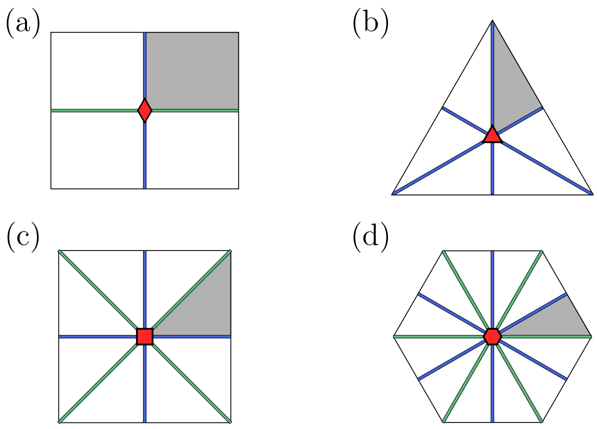

Supplementary Fig.3: Illustration of point groups D_n.(a) A rectangle under point group D_2, the shaded region is the fundamental domain. There are two conjugacy classes of reflection which are colored differently. (b) A triangle under point group D_3, the grey shaded region is the fundamental domain. There is only one conjugacy class of reflection. (c) A square under point group D_4, the shaded region is the fundamental domain. There are two conjugacy classes of reflection which are colored differently. (d) A hexagon under point group D_6, the shaded region is the fundamental domain. There are two conjugacy classes of reflection which are colored differently.

When n is odd, we can redefine $\mathsf{R} \to \mathsf{R}' = \pm\alpha_{rm}^{-1/2}\mathsf{R}$, which cancels factor α_r and α_{rm} simultaneously. Factor α_m can also be cancelled by redefining $\mathsf{M} \to \mathsf{M}' = \alpha_m^{-1/2}\mathsf{M}$. Thus we do not have any cohomology invariant in this case. When n is even, however, we can only reduce the relation to $\mathsf{R}'^n = \pm 1$ by redefining $\mathsf{R} \to \mathsf{R}' = \pm\alpha_{rm}^{-1/2}\mathsf{R}$. So there is one independent cohomology invariant $\alpha = \alpha_r \alpha_{rm}^{-n/2} = \pm 1$ in this case. The cohomology invariant $\alpha \in \{\pm 1\}$ is equal to the commutator of two pependicular reflection when n is even

$$\alpha = \left[\mathsf{M}, \mathsf{R}^{n/2}\mathsf{M}\right]. \tag{45}$$

These results are consistent with that of the group cohomology

$$H^2(D_n, U(1)) = \begin{cases} 1 & n \text{ odd} \\ \mathbb{Z}_2 & n \text{ even} \end{cases}. \tag{46}$$

For $\mathbb{Z}_2$ extension, $\alpha_{rm}, \alpha_m, \alpha_r \in \mathbb{Z}_2$. When n is odd, Eq. (43) has two solutions $\alpha_{rm} = 1, \alpha_r = \pm 1$. However, the nontrivial one $\alpha_{rm} = 1, \alpha_r = -1$ can be cancelled by redefining $\mathsf{R} \to \mathsf{R}' = \alpha_r \mathsf{R}$. The factor $\alpha_m = \pm 1$ cannot be reduced anymore, which is the only cohomology invariant in this case. When n is even, Eq. (43) is satisfied automatically, and we cannot reduce any of the three factors by redefining the generators, so we have three independent cohomology invariants $\alpha_r, \alpha_m, \alpha_{rm}$ in this case. The results are consistent with that of the group cohomology

$$H^2(D_n, \mathbb{Z}_2) = \begin{cases} \mathbb{Z}_2 & n \text{ odd} \\ \mathbb{Z}_2^3 & n \text{ even} \end{cases}. \tag{47}$$

The $\mathbb{Z}_2$ extensions of D_n can also be analyzed by another way based on conjugacy classes. For a group G, the conjugacy classe of group element r is defined as $C_s(r) \equiv \left\{grg^{-1} | g \in G\right\}$. Here is a general conclusion: Suppose r is an element of order n, i.e., $r^n = 1$. After extension, we have $\mathsf{r}^n = \alpha$. If r' is in the conjugate class $C_s(r)$, then it also satisfies $\mathsf{r}'^n = \alpha$ after extension. This conclusion is easy to show. Suppose $r' = grg^{-1}$, then after extension, we have

$$(\mathsf{r}')^n = (\mathsf{g}\mathsf{r}\mathsf{g}^{-1})^n = \mathsf{g}\mathsf{r}^n\mathsf{g}^{-1} = \mathsf{g}\alpha\mathsf{g}^{-1} = \alpha. \tag{48}$$

In wallpaper groups, rotation R and reflection M are both order n elements, so this conclusion can be applied to the conjugacy classes $C_s(R)$ and $C_s(M)$. This may help us to reduce the factors.

For group D_n, to apply the above conclusion, we rewrite Eq. (41) as

$$\mathsf{R}^n = \alpha_r \equiv \alpha_1, \tag{49a}$$
$$\mathsf{M}^2 = \alpha_m \equiv \beta_1, \tag{49b}$$
$$(\mathsf{RM})^2 = \alpha_{cm}\alpha_m \equiv \beta_2. \tag{49c}$$

When n is odd, we can set $n = 2k - 1$ for some integer k. Now, M and RM are two reflections and they belong to the same conjugacy class, which can be seen by

$$R^{1-k}MR^{-(1-k)} = R^{1-k}MR^{k-1}M^{-1}M$$
$$=R^{2-2k}M = R^{1-2k}RM = RM.$$

According to Eq. (48), reflections in the same conjugacy class have the same factor, so $\beta_1 = \beta_2$. On the other hand, as we show before, the factor α_1 of rotation can be cancelled when n is odd. Thus, we only have one independent cohomology invariant to label the projective representations of D_n in this case. When n is even, M and RM belong to different conjugacy classes, so β_1 and β_2 are independent. Futhermore, α_1 cannot be cancelled. Thus we have three independent cohomology invariants in this case.

b. P1

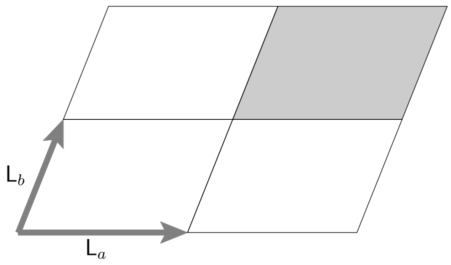

Supplementary Fig.4: Cell structure of $P1$. The shaded region is the fundamental domain.

The group $P1$ has two independent generators of translation L_a, L_b. The two generators commute with each other, $[L_a, L_b] = L_aL_bL_a^{-1}L_b^{-1} = 1$. So the presentation of $P1$ is given by

$$P1 = \langle L_a, L_b | [L_a, L_b] \rangle. \tag{50}$$

The projective algebra can be obtained by adding an additional factor to the relation,

$$\mathsf{L}_a\mathsf{L}_b\mathsf{L}_a^{-1}\mathsf{L}_b^{-1} = \sigma. \tag{51}$$

For $U(1)$ extension, σ takes values in $U(1)$, and for $\mathbb{Z}_2$ extension, σ takes values in $\mathbb{Z}_2$. σ is the cohomology invariant. These results are consistent with that of the group cohomology

$$H^2(P1, U(1)) = U(1), \tag{52}$$
$$H^2(P1, \mathbb{Z}_2) = \mathbb{Z}_2. \tag{53}$$

c. P2

The group $P2$ is obtained by adding two-fold rotation to $P1$. The generator of rotation R reverses the directions of translation L_a, L_b, so the the presentation of $P2$ is given by

$$P2 = \langle L_a, L_b, R | [L_a, L_b], RL_aR^{-1} = L_a^{-1}, RL_bR^{-1} = L_b^{-1}, R^2 \rangle. \tag{54}$$

In group $P2$, there are four different conjugacy classes of rotation $C_s(R)$, $C_s(L_aR)$, $C_s(L_bR)$, $C_s(L_aL_bR)$, as shown in Fig. 5 with four different colors. The relations of presentation can also be expressed in terms of the squares of the four rotation centers:

$$P2 = \langle L_a, L_b, R | R^2, (L_aR)^2, (L_bR)^2, (L_aL_bR)^2 \rangle. \tag{55}$$

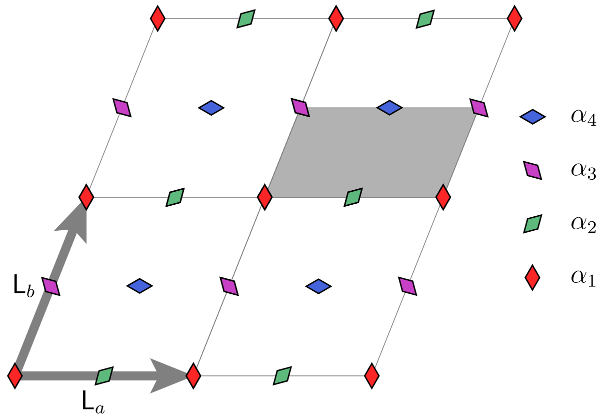

Supplementary Fig.5: Cell structure of $P2$. The shaded region is the fundamental domain. There are four different classes of rotations in group $P2$, their corresponding rotation centers are drawn in different colors. Rotation centers in the same class have the same factor.

When we consider projective algebra, relations of presentation (54) become

$$\mathsf{L}_a\mathsf{L}_b\mathsf{L}_a^{-1}\mathsf{L}_b^{-1} = \sigma, \tag{56a}$$
$$\mathsf{R}\mathsf{L}_a\mathsf{R}^{-1}\mathsf{L}_a = \eta_a, \tag{56b}$$
$$\mathsf{R}\mathsf{L}_b\mathsf{R}^{-1}\mathsf{L}_b = \eta_b, \tag{56c}$$
$$\mathsf{R}^2 = \alpha. \tag{56d}$$

For $\mathbb{Z}_2$ extension, four factors $\sigma, \eta_x, \eta_y, \alpha \in \mathbb{Z}_2$ serve as independent cohomology invariants of projective representations.

For $U(1)$ extension, factors η_a, η_b, α can be cancelled by redefining $\mathsf{L}_a \to \mathsf{L}'_a = \eta_a^{-1/2}\mathsf{L}_a, \mathsf{L}_b \to \mathsf{L}'_b = \eta_b^{-1/2}\mathsf{L}_b, \mathsf{R} \to \mathsf{R}' = \alpha^{-1/2}\mathsf{R}$. Thus we only have one independent cohomology invariant $\sigma \in U(1)$.

These results are consistent with that of the group cohomology

$$H^2(P2, \mathbb{Z}_2) = \mathbb{Z}_2^4, \tag{57a}$$
$$H^2(P2, U(1)) = U(1). \tag{57b}$$

For $\mathbb{Z}_2$ extension, we can also obtain the projective algebra by modifying the relations in Eq. (55) as

$$\mathsf{R}^2 = \alpha \equiv \alpha_1, \tag{58a}$$
$$(\mathsf{L}_a\mathsf{R})^2 = \eta_a\alpha \equiv \alpha_2, \tag{58b}$$
$$(\mathsf{L}_b\mathsf{R})^2 = \eta_b\alpha \equiv \alpha_3, \tag{58c}$$
$$(\mathsf{L}_a\mathsf{L}_b\mathsf{R})^2 = \sigma\eta_a\eta_b\alpha \equiv \alpha_4. \tag{58d}$$

These relations mean that each conjugacy class of rotation has an independent cohomology invariant, as shown in Fig. 5.

d. Pm

For group Pm, two translations L_x, L_y are in perpendicular directions. The reflection M_x reverses L_x to L_x^{-1} but leaves L_y invariant. The presentation is given by

$$\begin{aligned} Pm = \quad & \langle L_x, L_y, M_x | [L_x, L_y], M_x L_x M_x^{-1} = L_x^{-1}, \\ & M_x L_y M_x^{-1} = L_y, M_x^2 \rangle. \end{aligned} \tag{59}$$

There are two conjugacy classes of reflection $C_s(M_x), C_s(L_x M_x)$. They are parallel to each other, with a distance of half of the lattice constant in the x direction, as shown in Fig. 6. The presentation can also be written in terms of

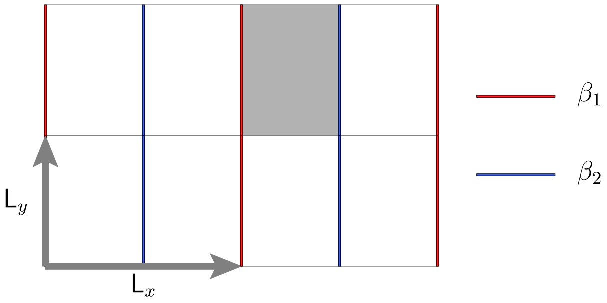

Supplementary Fig.6: Cell structure of Pm. There are two conjugacy classes of reflection in Pm. The gray shaded region is the fundamental domain of the group.

squares of the two reflections M_x, L_xM_x and their commutators with L_y,

$$Pm = \langle L_x, L_y, M_x | [M_x, L_y], [L_xM_x, L_y], (L_xM_x)^2, M_x^2 \rangle. \tag{60}$$

The projective relations of the presentation Eq. (59) are

$$\mathsf{L}_x\mathsf{L}_y\mathsf{L}_x^{-1}\mathsf{L}_y^{-1} = \sigma, \tag{61a}$$
$$\mathsf{M}_x\mathsf{L}_y\mathsf{M}_x^{-1}\mathsf{L}_y^{-1} = \eta_y, \tag{61b}$$
$$\mathsf{M}_x\mathsf{L}_x\mathsf{M}_x^{-1}\mathsf{L}_x = \eta_x, \tag{61c}$$
$$\mathsf{M}_x^2 = \alpha. \tag{61d}$$

The self-consistency condition requires $\sigma, \eta_y \in \{\pm 1\}$, this can be derived by conjugate relations Eq.(61)(a)(d).

$$\begin{aligned}\sigma &= \mathsf{M}_x\sigma\mathsf{M}_x^{-1} = \mathsf{M}_x\mathsf{L}_x\mathsf{L}_y\mathsf{L}_x^{-1}\mathsf{L}_y^{-1}\mathsf{M}_x^{-1} \\ &= \mathsf{L}_x^{-1}\mathsf{L}_y\mathsf{L}_x\mathsf{L}_y^{-1} = (\mathsf{L}_y\mathsf{L}_x^{-1}\mathsf{L}_y^{-1}\mathsf{L}_x)^{-1} = \sigma^{-1},\end{aligned} \tag{62}$$
$$\alpha = \mathsf{L}_y\alpha\mathsf{L}_y^{-1} = \mathsf{L}_y\mathsf{M}_x^2\mathsf{L}_y^{-1} = \eta_y^{-2}\alpha^2. \tag{63}$$

Factors η_x, α are trivial in $U(1)$ projective representations because they can be cancelled by redefining $\mathsf{L}_x \to \mathsf{L}'_x = \eta_x^{-1/2}\mathsf{L}_x, \mathsf{M}_x \to \mathsf{M}'_x = \alpha^{-1/2}\mathsf{M}_x$. Thus, we have two independent cohomology invariants $\sigma, \eta_y \in \{\pm 1\}$.

For $\mathbb{Z}_2$ extension, $\sigma, \eta_x, \eta_y, \alpha \in \{\pm 1\}$ are four nontrivial independent cohomology invariants.

Above results are consistent with that of the group cohomology

$$H^2(Pm, \mathbb{Z}_2) = \mathbb{Z}_2^4, \tag{64a}$$
$$H^2(Pm, U(1)) = \mathbb{Z}_2^2. \tag{64b}$$

For $\mathbb{Z}_2$ extension, we can also obtain the projective algebra by modifying the relations in Eq. (60) as

$$(\mathsf{M}_x)^2 = \alpha \equiv \beta_1, \tag{65a}$$
$$(\mathsf{L}_x\mathsf{M}_x)^2 = \eta_x\alpha \equiv \beta_2, \tag{65b}$$
$$[\mathsf{M}_x, \mathsf{L}_y] = \eta_y \equiv \eta_1, \tag{65c}$$
$$[\mathsf{L}_x\mathsf{M}_x, \mathsf{L}_y] = \sigma\eta_y \equiv \eta_2. \tag{65d}$$

The first two relations means that the two classes of reflections have two independent cohomology invariants, and the the last two relations means that they also have two cohomology invariants which are their commutators with L_y.

e. Pg

The group Pg contains glide-reflection operations. Here we suppose the refletion operation reverses the x direction, so the glide-reflection operator is $g_x = L_{\frac{y}{2}}M_x$, where $L_{\frac{y}{2}}$ is a translation in the y direction with half lattice constant.

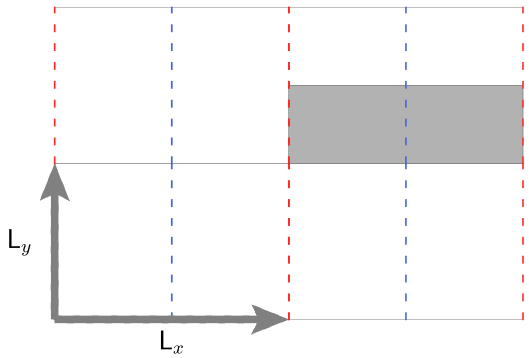

Supplementary Fig.7: Cell structure of Pg. The gray shaded region is the fundamental domain of the group.

Since $g_x^2 = L_y$, we can take L_x and g_x as generators of group Pg. Because the glide-reflection g_x reverses the direction of L_x, the presentation is given by

$$Pg = \langle g_x, L_y | g_x L_x g_x^{-1} = L_x^{-1} \rangle. \tag{66}$$

The projective relations of presentation Eq. (66) is

$$\mathsf{g}_x \mathsf{L}_x \mathsf{g}_x^{-1} = \tau \mathsf{L}_x^{-1}. \tag{67}$$

For $\mathbb{Z}_2$ extension, $\tau \in \{\pm 1\}$ is the only one independent cohomology invariant. For $U(1)$ extension, τ is trivial because we can cancel it by redefining $\mathsf{L}_x \to \mathsf{L}'_x = \tau^{-1/2}\mathsf{L}_x$. These are consistent with the results of the group cohomology

$$H^2(Pg, \mathbb{Z}_2) = \mathbb{Z}_2, \tag{68a}$$
$$H^2(Pg, U(1)) = 1. \tag{68b}$$

f. Cm

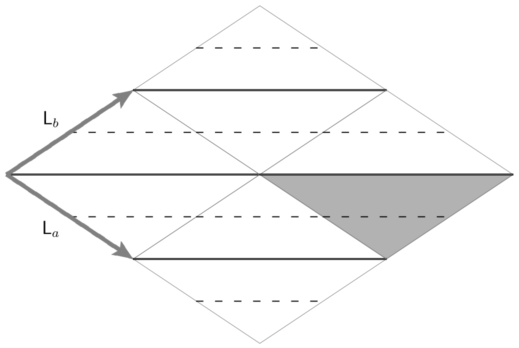

Supplementary Fig.8: Cell structure of Cm. The gray shaded region is the fundamental domain of the group.

For group Cm the reflection M interchanges the two translations L_a, L_b. The presentation is given by

$$Cm = \langle L_a, L_b, M | [L_a, L_b], M L_a M^{-1} = L_b, M^2 \rangle. \tag{69}$$

When we consider projective algebra, relations of presentation become

$$\mathsf{L}_a \mathsf{L}_b \mathsf{L}_a^{-1} \mathsf{L}_b^{-1} = \sigma, \tag{70a}$$
$$\mathsf{M}\mathsf{L}_a \mathsf{M}^{-1} = \mathsf{L}_b, \tag{70b}$$
$$\mathsf{M}^2 = \beta. \tag{70c}$$

Notice that (70b) does not contribute a factor because the factor $\eta = \mathsf{M}\mathsf{L}_a\mathsf{M}^{-1}\mathsf{L}_b^{-1}$ can be trivialized by redefining $\mathsf{L}_b \to \mathsf{L}_b' = \eta^{-1}\mathsf{L}_b$.

For $\mathbb{Z}_2$ extension, we have two independent cohomology invariants $\sigma, \alpha \in \mathbb{Z}_2$.

For $U(1)$ extension, $\sigma \in \mathbb{Z}_2$ because

$$\begin{aligned}\sigma &= \mathsf{M}\sigma\mathsf{M}^{-1} = \mathsf{M}\mathsf{L}_a\mathsf{L}_b\mathsf{L}_a^{-1}\mathsf{L}_b^{-1}\mathsf{M}^{-1}\\ &= \mathsf{L}_b\mathsf{L}_a\mathsf{L}_b^{-1}\mathsf{L}_a^{-1} = (\mathsf{L}_a\mathsf{L}_b\mathsf{L}_a^{-1}\mathsf{L}_b^{-1})^{-1} = \sigma^{-1}. \end{aligned} \tag{71}$$

The factor α is trivial in $U(1)$ extension because we can cancel it by redefining $\mathsf{M} \to \mathsf{M}' = \alpha^{-1/2}\mathsf{M}$. Thus we have only one cohomology invariant $\sigma \in \mathbb{Z}_2$.

These results are consistent with that of the group cohomology

$$H^2(Cm, \mathbb{Z}_2) = \mathbb{Z}_2^2, \tag{72a}$$

$$H^2(Cm, U(1)) = \mathbb{Z}_2. \tag{72b}$$

g. Pmm

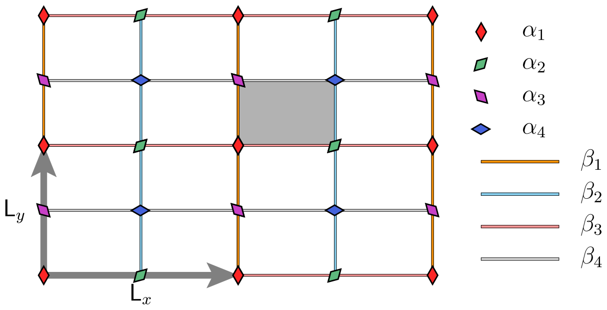

Supplementary Fig.9: Cell structure of Pmm. There are four different conjugacy classes of rotations and reflections respectively. The gray shaded region is the fundamental domain of the group.

The group Pmm contains two perpendicular reflection M_x, M_y. The combination M_xM_y is a two-fold rotation R. The two reflections commute with each other. M_x reverses L_x and preserves L_y, while M_y preserves L_x and reverses L_y. The presentation of Pmm is

$$\begin{aligned} Pmm = \quad & \langle L_x, L_y, M_x, M_y | [L_x, L_y], M_xL_xM_x^{-1} = L_x^{-1},\\ & M_xL_yM_x^{-1} = L_y, M_yL_yM_y^{-1} = L_y^{-1},\\ & M_yL_xM_y^{-1} = L_x, M_x^2, M_y^2, [M_x, M_y]\rangle. \end{aligned} \tag{73}$$

In group Pmm, there are four different conjugacy classes of rotation centers $C_s(R), C_s(L_xR)$, $C_s(L_yR), C_s(L_xL_yR)$, and four different conjugacy classes of reflection axes $C_s(M_y), C_s(L_yM_y)$, $C_s(M_x), C_s(L_xM_x)$, as shown in Fig. 9.

The relations of presentation can also be expressed in terms of the squares of the four rotations and the four reflections:

$$\begin{aligned} Pmm = \quad & \langle L_x, L_y, M_x, M_y | (M_xM_y)^2, (L_xM_xM_y)^2,\\ & (L_yM_xM_y)^2, (L_xL_yM_xM_y)^2, M_y^2,\\ & (L_yM_y)^2, M_x^2, (L_xM_x)^2\rangle. \end{aligned} \tag{74}$$

When we consider projective algebra, relations of presentation become

$$\mathsf{L}_x\mathsf{L}_y\mathsf{L}_x^{-1}\mathsf{L}_y^{-1} = \sigma, \tag{75a}$$
$$\mathsf{M}_x\mathsf{L}_x\mathsf{M}_x^{-1} = \eta_{m_x x}\mathsf{L}_x^{-1}, \tag{75b}$$
$$\mathsf{M}_x\mathsf{L}_y\mathsf{M}_x^{-1}\mathsf{L}_y^{-1} = \eta_{m_x y}, \tag{75c}$$
$$\mathsf{M}_y\mathsf{L}_y\mathsf{M}_y^{-1} = \eta_{m_y y}\mathsf{L}_y^{-1}, \tag{75d}$$
$$\mathsf{M}_y\mathsf{L}_x\mathsf{M}_y^{-1}\mathsf{L}_x^{-1} = \eta_{m_y x}, \tag{75e}$$
$$\mathsf{M}_x^2 = \alpha_x, \tag{75f}$$
$$\mathsf{M}_y^2 = \alpha_y, \tag{75g}$$
$$\mathsf{M}_x\mathsf{M}_y\mathsf{M}_x^{-1}\mathsf{M}_y^{-1} = \alpha_{xy}. \tag{75h}$$

For $\mathbb{Z}_2$ extension, $\sigma, \eta_{m_x x}, \eta_{m_x y}, \eta_{m_y y}, \eta_{m_y x}, \alpha_x, \alpha_y, \alpha_{xy} \in \mathbb{Z}_2$, and we have eight independent cohomology invariants.
For $U(1)$ extension $\sigma, \eta_{m_x y}, \eta_{m_y x}, \alpha_{xy} \in \mathbb{Z}_2$ due to the self-consistency condition in the presence of reflection symmetries, and the proof is similar to Eq. (62) in the case of group Pm. Factors $\eta_{m_x x}, \eta_{m_y y}, \alpha_x, \alpha_y$ are trivial because we can cancel them by redefining $\mathsf{L}_x \to \mathsf{L}'_x = \eta_{m_x x}^{-1/2}\mathsf{L}_x, \mathsf{L}_y \to \mathsf{L}'_y = \eta_{m_y y}^{-1/2}\mathsf{L}_y, \mathsf{M}_x \to \mathsf{M}'_x = \alpha_x^{-1/2}\mathsf{M}_x, \mathsf{M}_y \to \mathsf{M}'_y = \alpha_y^{-1/2}\mathsf{M}_y$. Thus, we only have four independent $\mathbb{Z}_2$ cohomology invariants.
Above results are consistent with that of the group cohomology

$$H^2(Pmm, \mathbb{Z}_2) = \mathbb{Z}_2^8, \tag{76a}$$
$$H^2(Pmm, U(1)) = \mathbb{Z}_2^4. \tag{76b}$$

For $\mathbb{Z}_2$ extension,we can also obtain the projective algebra by modifying the relations in Eq. (74) as

$$\mathsf{R}^2 = (\mathsf{M}_x\mathsf{M}_y)^2 = \alpha_r \equiv \alpha_1, \tag{77a}$$
$$(\mathsf{L}_x\mathsf{R})^2 = \eta_{rx}\alpha_r \equiv \alpha_2, \tag{77b}$$
$$(\mathsf{L}_y\mathsf{R})^2 = \eta_{ry}\alpha_r \equiv \alpha_3, \tag{77c}$$
$$(\mathsf{L}_x\mathsf{L}_y\mathsf{R})^2 = \sigma\eta_{rx}\eta_{ry}\alpha_r \equiv \alpha_4, \tag{77d}$$
$$\mathsf{M}_x^2 = \alpha_x \equiv \beta_1, \tag{77e}$$
$$(\mathsf{L}_x\mathsf{M}_x)^2 = \eta_{m_x x}\alpha_x \equiv \beta_2, \tag{77f}$$
$$\mathsf{M}_y^2 = \alpha_y \equiv \beta_3, \tag{77g}$$
$$(\mathsf{L}_y\mathsf{M}_y)^2 = \eta_{m_y y}\alpha_y \equiv \beta_4. \tag{77h}$$

where

$$\alpha_r \equiv \alpha_x\alpha_y\alpha_{xy}, \tag{78a}$$
$$\eta_{rx} \equiv \eta_{m_x x}\eta_{m_y x}, \tag{78b}$$
$$\eta_{ry} \equiv \eta_{m_x y}\eta_{m_y y}. \tag{78c}$$

Eq. (77) means that each conjugacy class of rotations and reflections has an independent cohomology invariant, as Fig. 9 shows.

h. Pmg

If we replace reflection M_y by glide-reflection g_y in group Pmm, we get group Pmg. Since M_x reverses g_y and preserves L_y. The presentation of Pmg is given by

$$Pmg = \langle g_y, L_y, M_x | g_y L_y g_y^{-1} = L_y^{-1}, M_x L_y M_x^{-1} = L_y, M_x g_y M_x^{-1} = g_y^{-1}, M_x^2 \rangle. \tag{79}$$

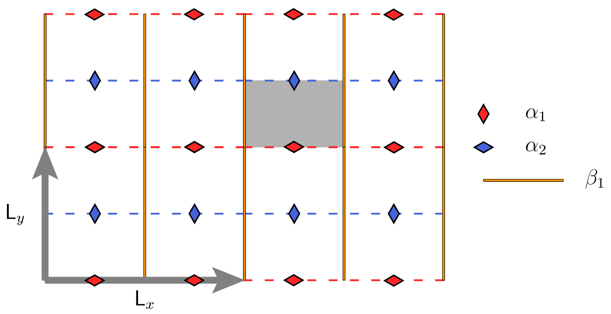

Supplementary Fig.10: Cell structure of Pmg. There are two conjugacy classes of rotations and one conjugacy class of reflections. Centers of rotations all lie on glide-reflection axes. The gray shaded region is the fundamental domain of the group.

There are two conjugacy classes of rotation $C_s(M_x g_y)$, $C_s(L_y M_x g_y)$ and one conjugacy class of reflection $C_s(M_x)$ in Pmg. The presentation can also be given in terms of the commutator $[M_x, L_y]$ and the square of each rotation and reflection,

$$\begin{aligned} Pmg \quad &= \langle g_y, L_y, M_x | [M_x, L_y], (L_y M_x g_y)^2, \\ &(M_x g_y)^2, M_x^2 \rangle. \end{aligned} \tag{80}$$

Projective relations of Eq. (79) are

$$\mathsf{g}_y \mathsf{L}_y \mathsf{g}_y^{-1} = \tau_{g_y y} \mathsf{L}_y^{-1}, \tag{81a}$$
$$\mathsf{M}_x \mathsf{L}_y \mathsf{M}_x^{-1} \mathsf{L}_y^{-1} = \eta_{m_x y}, \tag{81b}$$
$$\mathsf{M}_x \mathsf{g}_y \mathsf{M}_x^{-1} \mathsf{g}_y = \alpha_{m_x g_y}, \tag{81c}$$
$$\mathsf{M}_x^2 = \alpha_{m_x}. \tag{81d}$$

For $\mathbb{Z}_2$ extension, we have four independent cohomology invariants $\tau_{g_y y}$, $\eta_{m_x y}$, $\alpha_{m_x g_y}, \alpha_{m_x} \in \mathbb{Z}_2$.

For $U(1)$ extension, we have only one cohomology invariant $\eta_{m_x y}, \in \mathbb{Z}_2$ (the $\mathbb{Z}_2$ value is due to the self-consistency condition). Factors $\tau_{g_y y}$, $\alpha_{m_x g_y}$, α_{m_x} can be cancelled by redefining $\mathsf{L}_y \to \mathsf{L}'_y = \tau_{g_y y}^{-1/2} \mathsf{L}_y, \mathsf{g}_y \to \mathsf{g}'_y = \alpha_{m_x g_y}^{-1/2} \mathsf{g}_y, \mathsf{M}_x \to \mathsf{M}'_x = \alpha_{m_x}^{-1/2} \mathsf{M}_x$.

Above results are consistent with that of the group cohomology

$$H^2(Pmg, \mathbb{Z}_2) = \mathbb{Z}_2^4, \tag{82a}$$
$$H^2(Pmg, U(1)) = \mathbb{Z}_2. \tag{82b}$$

For $\mathbb{Z}_2$ extension, we can also obtain the projective algebra by modifying the relations in Eq. (80) as

$$(\mathsf{M}_x \mathsf{g}_y)^2 = \alpha_r \equiv \alpha_1, \tag{83a}$$
$$(\mathsf{L}_y \mathsf{M}_x \mathsf{g}_y)^2 = \eta_{ry} \alpha_r \equiv \alpha_2, \tag{83b}$$
$$\mathsf{M}_x^2 = \alpha_{m_x} \equiv \beta, \tag{83c}$$
$$\mathsf{M}_x \mathsf{L}_y \mathsf{M}_x^{-1} \mathsf{L}_y^{-1} = \eta_{m_x y} \equiv \eta. \tag{83d}$$

where

$$\alpha_r \equiv \alpha_{m_x g_y} \alpha_{m_x}, \tag{84a}$$
$$\eta_{ry} \equiv \eta_{m_x y} \tau_{g_y y}. \tag{84b}$$

Eq. (83) means that each conjugacy class of rotations and reflections has an independent cohomology invariants, and the commutator of the reflection and L_y is also a cohomology invariant.

i. Pgg

The group Pgg has two perpendicular glide-reflections g_x, g_y. Since $g_x^2 = L_y, g_y^2 = L_x$, we can take g_x, g_y as generators. If we choose the intersection point of the two glide axes as origin, then $g_x g_y$ and $g_x g_y^{-1}$ act on coordinates

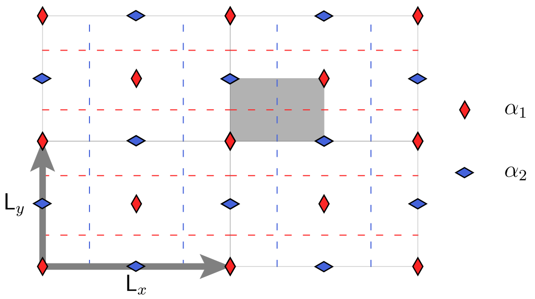

Supplementary Fig.11: Cell structure of Pgg. The two glide reflections are in perpendicular directions. There are two conjugacy classes of rotation. The rotation centers do not lie on glide-reflection axes. The gray shaded region is the fundamental domain.

as $g_xg_y(x,y) = g_y(x+1/2,-y) = (-x-1/2,-y+1/2)$, $g_xg_y^{-1}(x,y) = g_x(x-1/2,-y) = (-x+1/2,-y+1/2)$. So they are R_π rotation operations around $(-1/4,-1/4)$ and $(1/4,1/4)$ respectively, as shown in Fig. 11. The presentation of Pgg can be given by

$$Pgg = \langle g_x, g_y | (g_xg_y)^2, (g_xg_y^{-1})^2\rangle. \tag{85}$$

When we consider projective algebra, relations of the presentation become

$$(\mathsf{g}_x\mathsf{g}_y)^2 = \alpha \equiv \alpha_1, \tag{86a}$$
$$(\mathsf{g}_x\mathsf{g}_y^{-1})^2 = \tau\alpha \equiv \alpha_2. \tag{86b}$$

.

For $\mathbb{Z}_2$ extension, there are two cohomology invariants $\alpha, \tau \in \mathbb{Z}_2$, while for $U(1)$ extension these factors are trivial because we can cancel them by redefining $\mathsf{g}_x \to \mathsf{g}_x' = \alpha^{-1/2}\tau^{-1/4}\mathsf{g}_x, \mathsf{g}_y \to \mathsf{g}_y' = \tau^{1/4}\mathsf{g}_y$. These are in agreement with the results of group cohomology

$$H^2(Pgg, \mathbb{Z}_2) = \mathbb{Z}_2^2, \tag{87a}$$
$$H^2(Pgg, U(1)) = 1. \tag{87b}$$

We can also get cohomology invariants between $\mathsf{g}_y, \mathsf{g}_x$ and translation operator $\mathsf{L}_x = \mathsf{g}_y^2$, $\mathsf{L}_y = \mathsf{g}_x^2$,

$$\begin{aligned} & \mathsf{L}_x\mathsf{g}_x\mathsf{L}_x\mathsf{g}_x^{-1} = \mathsf{g}_y^2\mathsf{g}_x\mathsf{g}_y^2\mathsf{g}_y^{-1} \\ = \; & \mathsf{g}_y(\mathsf{g}_y\mathsf{g}_x\mathsf{g}_y)\mathsf{g}_y\mathsf{g}_x^{-1} = \alpha\mathsf{g}_y\mathsf{g}_x^{-1}\mathsf{g}_y\mathsf{g}_x^{-1} = \tau, \end{aligned} \tag{88a}$$
$$\begin{aligned} & \mathsf{L}_y\mathsf{g}_y\mathsf{L}_y\mathsf{g}_y^{-1} = \mathsf{g}_x^2\mathsf{g}_y\mathsf{g}_x^2\mathsf{g}_y^{-1} \\ = \; & \mathsf{g}_x(\mathsf{g}_x\mathsf{g}_y\mathsf{g}_x)\mathsf{g}_x\mathsf{g}_y^{-1} = \alpha\mathsf{g}_x\mathsf{g}_y^{-1}\mathsf{g}_x\mathsf{g}_y^{-1} = \tau. \end{aligned} \tag{88b}$$

j. Cmm

If we add another reflection perpendicular to the reflection of group Cm, we get group Cmm. The two perpendicular reflections in Cmm commute with each other. One reflection transforms L_a to L_b while the other transforms L_a to L_b^{-1}. Thus, the presentation is given by

$$\begin{aligned} Cmm = \; & \langle L_a, L_b, M_x, M_y | [L_a, L_b], M_xL_aM_x^{-1} = L_b^{-1}, \\ & M_yL_aM_y^{-1} = L_b, [M_x, M_y], M_x^2, M_y^2\rangle. \end{aligned} \tag{89}$$

There are three conjugacy classes of rotations $C_s(M_xM_y)$, $C_s(L_aM_xM_y)$, $C_s(L_aM_xL_aM_y)$ and two conjugacy classes of reflections $C_s(M_x)$, $C_s(M_y)$. We can also present the group by

$$\begin{aligned} Cmm = \; & \langle L_a, M_x, M_y | (M_xM_y)^2, (L_aM_xM_y)^2, \\ & (L_aM_xL_a^{-1}M_y)^2, M_x^2, M_y^2\rangle. \end{aligned} \tag{90}$$

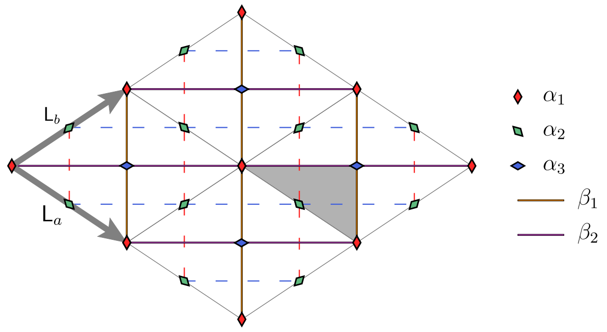

Supplementary Fig.12: Cell structure of Cmm. There are two (conjugacy class of) reflections in perpendicular directions. One class of rotation centers are not on reflection axes, while the other two classes of rotation centers are on reflection axes. The gray shaded region is the fundamental domain of the group.

When we consider projective algebra, relations of the presentation Eq. (89) can be modified as

$$\mathsf{L}_a\mathsf{L}_b\mathsf{L}_a^{-1}\mathsf{L}_b^{-1} = \sigma, \tag{91a}$$
$$\mathsf{M}_y\mathsf{L}_a\mathsf{M}_y^{-1} = \eta_1\mathsf{L}_b, \tag{91b}$$
$$\mathsf{M}_x\mathsf{L}_a\mathsf{M}_x^{-1} = \eta_2\mathsf{L}_b^{-1}, \tag{91c}$$
$$\mathsf{M}_y\mathsf{M}_x\mathsf{M}_y^{-1}\mathsf{M}_x^{-1} = \alpha_{xy}, \tag{91d}$$
$$\mathsf{M}_x^2 = \alpha_x, \tag{91e}$$
$$\mathsf{M}_y^2 = \alpha_y. \tag{91f}$$

For $\mathbb{Z}_2$ extension, factor η_1 or η_2 can be cancelled by redefining generators, but they cannot be cancelled simultaneously. The factor $\eta = \eta_1\eta_2$ is a cohomology invariant. We have five independent cohomology invariants $\sigma, \eta, \alpha_{xy}, \alpha_x, \alpha_y \in \mathbb{Z}_2$.

For $U(1)$ extension, we have two independent cohomology invariants $\sigma, \alpha_{xy} \in \mathbb{Z}_2$ (the $\mathbb{Z}_2$ value is due to the self-consistency condition). Factors $\eta_1, \eta_2, \alpha_x, \alpha_y$ are trivial because we can cancel them by redefining $\mathsf{L}_a \to \mathsf{L}'_a = (\eta_1\eta_2)^{-1/2}\mathsf{L}_a, \mathsf{L}_b \to \mathsf{L}'_b = (\eta_1\eta_2^{-1})^{1/2}\mathsf{L}_b, \mathsf{M}_x \to \mathsf{M}'_x = \alpha_x^{-1/2}\mathsf{M}_x, \mathsf{M}_y \to \mathsf{M}'_y = \alpha_y^{-1/2}\mathsf{M}_y$. These are consistent with the results of the group cohomology

$$H^2(Cmm, \mathbb{Z}_2) = \mathbb{Z}_2^5, \tag{92a}$$
$$H^2(Cmm, U(1)) = \mathbb{Z}_2^2. \tag{92b}$$

For $\mathbb{Z}_2$ extension, we can also obtain the projective algebra by modifying the relations in Eq. (90) as

$$\mathsf{R}^2 = (\mathsf{M}_x\mathsf{M}_y)^2 = \alpha_r \equiv \alpha_1, \tag{93a}$$
$$(\mathsf{L}_a\mathsf{R})^2 = (\mathsf{L}_a\mathsf{M}_x\mathsf{M}_y)^2 = \eta\alpha_r \equiv \alpha_2, \tag{93b}$$
$$(\mathsf{L}_a\mathsf{L}_b\mathsf{R})^2 = (\mathsf{L}_a\mathsf{M}_x\mathsf{L}_a^{-1}\mathsf{M}_y)^2 = \sigma\alpha_r \equiv \alpha_3, \tag{93c}$$
$$\mathsf{M}_x^2 = \alpha_x \equiv \beta_1, \tag{93d}$$
$$\mathsf{M}_y^2 = \alpha_y \equiv \beta_2, \tag{93e}$$

where

$$\alpha_r \equiv \alpha_x\alpha_y\alpha_{xy}. \tag{94a}$$

These relations mean that each conjugacy class of rotations and reflections has an independent cohomology invariant, as shown in Fig. 12.

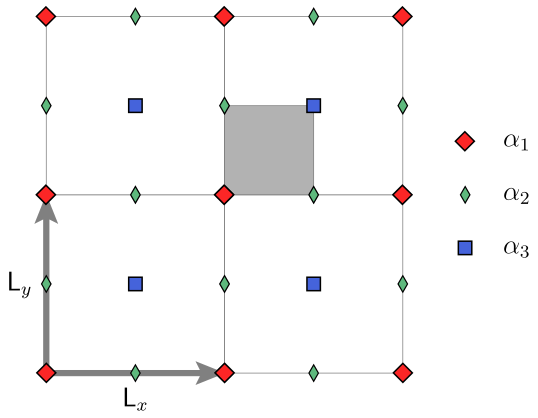

Supplementary Fig.13: Cell structure of $P4$. There are three conjugacy classes of rotation centers. The gray shaded region is the fundamental domain of the group.

k. P4

The group $P4$ contains a four-fold rotation, which rotates translations L_x, L_y as $RL_xR^{-1} = L_y, RL_yR^{-1} = L_x^{-1}$. The presentation is given by

$$\begin{aligned} P4 = \quad & \langle L_x, L_y, R | [L_x, L_y], RL_xR^{-1} = L_y, \\ & RL_yR^{-1} = L_x^{-1}, R^4 \rangle. \end{aligned} \tag{95}$$

There are two conjugacy classes of four-fold rotation $C_s(R), C_s(L_xR)$ and one conjugacy class of two-fold rotation $C_s(L_xR^2)$, as shown in Fig. 13. The presentation can also be given by

$$P4 = \langle L_x, R | (L_xR)^4, (L_xR^2)^2, R^4 \rangle. \tag{96}$$

When we consider projective algebra, the relations of presentation (95) become

$$\mathsf{L}_x\mathsf{L}_y\mathsf{L}_x^{-1}\mathsf{L}_y^{-1} = \sigma, \tag{97a}$$
$$\mathsf{R}\mathsf{L}_x\mathsf{R}^{-1} = \eta_1\mathsf{L}_y, \tag{97b}$$
$$\mathsf{R}\mathsf{L}_y\mathsf{R}^{-1} = \eta_2\mathsf{L}_x^{-1}, \tag{97c}$$
$$\mathsf{R}^4 = \alpha. \tag{97d}$$

For $U(1)$ extension, we have only one independent cohomology invariant $\sigma \in U(1)$. η_1, η_2, α are trivial because we can cancel them by redefining $\mathsf{L}_x \to \mathsf{L}'_x = (\eta_1\eta_2)^{-1/2}\mathsf{L}_x, \mathsf{L}_y \to \mathsf{L}'_y = (\eta_1\eta_2^{-1})^{1/2}\mathsf{L}_y, \mathsf{R} \to \mathsf{R}' = \alpha^{-1/4}\mathsf{R}$.

For $\mathbb{Z}_2$ extension, although η_1 or η_2 can be cancelled by redefinition, $\eta = \eta_1\eta_2$ is a cohomology invariant. We have three independent cohomology invariants $\sigma, \eta, \alpha \in \mathbb{Z}_2$.

These results are consistent with that of the group cohomology

$$H^2(P4, U(1)) = U(1), \tag{98a}$$
$$H^2(P4, \mathbb{Z}_2) = \mathbb{Z}_2^3. \tag{98b}$$

For $\mathbb{Z}_2$ extension, we can also obtain the projective algebra by modifying the relations in Eq. (96) as

$$\mathsf{R}^4 = \alpha \equiv \alpha_1, \tag{99a}$$
$$(\mathsf{L}_x\mathsf{R}^2)^2 = \eta\alpha \equiv \alpha_2, \tag{99b}$$
$$(\mathsf{L}_x\mathsf{R})^4 = \sigma\alpha \equiv \alpha_3. \tag{99c}$$

These relations mean that each conjugacy class of rotation has an independent cohomology invariant, as shown in Fig. 13.

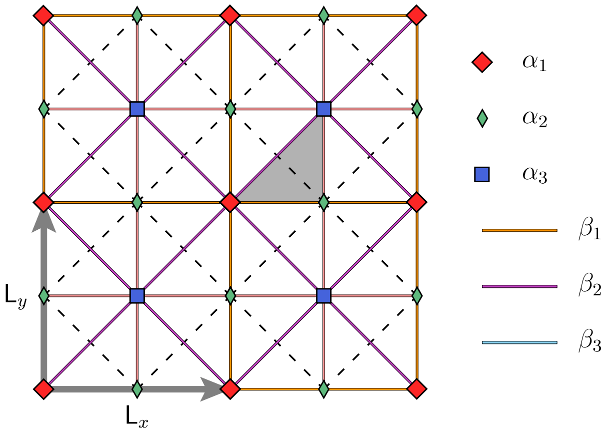

Supplementary Fig.14: Cell structure of $P4m$. There are three conjugacy classes of rotation and three conjugacy classes of reflection. The gray shaded region is the fundamental domain of the group.

l. P4m

If we add reflection symmetry to the group $P4$, we obtain group $P4m$. Here we choose the reflection M whose axis is perpendicular to $\boldsymbol{e}_x$ as a generator. M reverses translation L_x and rotation C. Thus, the presentation is given by

$$\begin{aligned} P4m = \quad & \langle L_x, L_y, R, M | [L_x, L_y], RL_xR^{-1} = L_y, \\ & RL_yR^{-1} = L_x^{-1}, ML_xM^{-1} = L_x^{-1}, \\ & MCM^{-1} = R^{-1}, R^4, M^{-1} \rangle. \end{aligned} \tag{100}$$

There are two conjugacy classes of four-fold rotation $C_s(R), C_S(L_xR)$, one conjugacy class of two-fold rotation $C_S(L_xR^2)$ and three conjugacy classes of reflection $C_S(M), C_S(RM), C_S(L_xM)$, as shown in Fig. 14. The group can also be presented in terms of rotations and reflections as

$$\begin{aligned} P4m = \quad & \langle L_x, R, M | (L_xC)^4, (L_xR^2)^2, R^4, \\ & (L_xM)^2, (RM)^2, M^2 \rangle. \end{aligned} \tag{101}$$

When we consider projective algebra, we can modify the relations of generators as

$$\mathsf{L}_x\mathsf{L}_y\mathsf{L}_x^{-1}\mathsf{L}_y^{-1} = \sigma, \tag{102a}$$
$$\mathsf{R}\mathsf{L}_x\mathsf{R}^{-1} = \eta_1\mathsf{L}_y, \tag{102b}$$
$$\mathsf{R}\mathsf{L}_y\mathsf{R}^{-1} = \eta_2\mathsf{L}_x^{-1}, \tag{102c}$$
$$\mathsf{M}\mathsf{L}_x\mathsf{M}^{-1} = \eta_m\mathsf{L}_x^{-1}, \tag{102d}$$
$$\mathsf{R}^4 = \alpha_r, \tag{102e}$$
$$\mathsf{M}^2 = \alpha_m, \tag{102f}$$
$$\mathsf{M}\mathsf{R}\mathsf{M}^{-1} = \alpha_{rm}\mathsf{R}^{-1}. \tag{102g}$$

Like other examples of group which contains reflection, the self-consistency condition requires $\sigma \in \mathbb{Z}_2$. Furthermore, R, M generate a projective algebra of D_4. As we analyzed before, factors α_{rm}, α_r must satisfy the relation Eq.(43) $\alpha_{rm}^4\alpha_r^{-2} = 1$, and α_r has two solutions $\alpha_r = \pm\alpha_{rm}^2 \equiv \alpha\alpha_{rm}^2$.

To see the requirement of self-consisitency condition on other factors, we conjugate Eq.(102)(b)(c) by M. Using Eq.(102)(d)(g), we have

$$\eta_m\mathsf{R}^{-1}\mathsf{L}_x^{-1}\mathsf{R} = \eta_1\mathsf{M}\mathsf{L}_y\mathsf{M}^{-1},$$
$$\mathsf{R}^{-1}\mathsf{M}\mathsf{L}_y\mathsf{M}^{-1}\mathsf{R} = \eta_2\eta_m^{-1}\mathsf{L}_x.$$

Cancel $\mathsf{M}\mathsf{L}_y\mathsf{M}^{-1}$ in the two equations above, we get

$$\eta_2\eta_m^{-1}\mathsf{L}_x = \eta_m\eta_1^{-1}\mathsf{R}^{-1}(\mathsf{R}^{-1}\mathsf{L}_x^{-1}\mathsf{R})\mathsf{R}.$$

Because $\mathsf{R}^{-1}\mathsf{L}_x^{-1}\mathsf{R} = \eta_2^{-1}\mathsf{L}_y$, $\mathsf{R}^{-1}\mathsf{L}_y\mathsf{R} = \eta_1^{-1}\mathsf{L}_x$, the above equation becomes

$$\eta_2\eta_m^{-1}\mathsf{L}_x = \eta_m\eta_1^{-2}\eta_2^{-1}\mathsf{L}_x.$$

Thus, η_1, η_2, η_m must satisfy

$$(\eta_m^{-1}\eta_1\eta_2)^2 = 1. \tag{103}$$

It has two solutions for η_m,

$$\eta_m = \pm 1\eta_1\eta_2 \equiv \eta(\eta_1\eta_2) \tag{104}$$

For $U(1)$ extension, factors η_1, η_2, α_m, α_{rm} can be trivialized by redefining $\mathsf{L}_x \to \mathsf{L}'_x = (\eta_1\eta_2)^{-1/2}\mathsf{L}_x, \mathsf{L}_y \to \mathsf{L}'_y = (\eta_1\eta_2^{-1})^{1/2}\mathsf{L}_y, \mathsf{R} \to \mathsf{R}' = \alpha_{rm}^{-1/2}\mathsf{R}, \mathsf{M} \to \mathsf{M}' = \alpha_m^{-1/2}\mathsf{M}$. We have three independent cohomology invariants $\sigma, \eta = \eta_1^{-1}\eta_2^{-1}\eta_m, \alpha = \alpha_r\alpha_{rm}^{-2} \in \mathbb{Z}_2$.

For $\mathbb{Z}_2$ extension, we have six independent cohomology invariants $\sigma, \eta_r = \eta_1\eta_2, \alpha_r, \eta_m, \alpha_m, \alpha_{rm} \in \mathbb{Z}_2$.

These results are in agreement with that of the group cohomology

$$H^2(p4m, U(1)) = \mathbb{Z}_2^3, \tag{105a}$$
$$H^2(p4m, \mathbb{Z}_2) = \mathbb{Z}_2^6. \tag{105b}$$

For $\mathbb{Z}_2$ extension, we can also obtain the projective algebra by modifying the relations in Eq. (101) as

$$\mathsf{R}^4 = \alpha_r \equiv \alpha_1, \tag{106a}$$
$$(\mathsf{L}_x\mathsf{R}^2)^2 = \eta_r\alpha_r \equiv \alpha_2, \tag{106b}$$
$$(\mathsf{L}_x\mathsf{R})^4 = \sigma\alpha_r \equiv \alpha_3, \tag{106c}$$
$$\mathsf{M}^2 = \alpha_m \equiv \beta_1, \tag{106d}$$
$$(\mathsf{RM})^2 = \alpha_{rm}\alpha_m \equiv \beta_2, \tag{106e}$$
$$(\mathsf{L}_x\mathsf{M})^2 = \eta_m\alpha_m \equiv \beta_3. \tag{106f}$$

These relations mean that each conjugacy class of rotations and reflections has an independent cohomology invariant, as shown in Fig. 14.

m. P4g

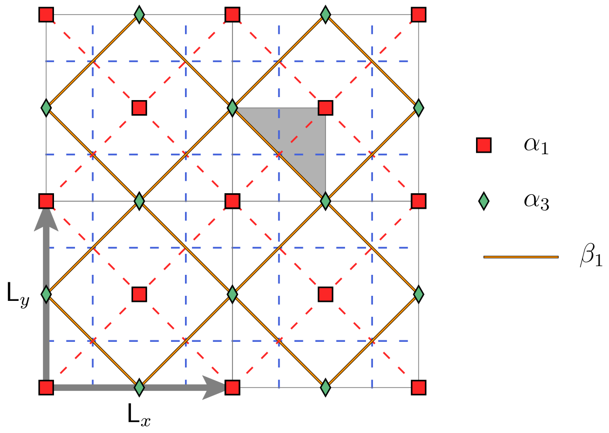

Supplementary Fig.15: Cell structure of $P4g$. There are two conjugacy classes of rotation and one conjugacy class of reflection. The gray shaded region is the fundamental domain of the group.

For group $P4g$, we can take g_y, R as generators. If we choose origin as the rotation center of R, then R, g_y act on coordinates as $R(x,y) = (-y,x)$,$g_y(x,y) = (x+1/2, -y+1/2)$. $L_xR^2 = g_y^2R^2$ is a two-fold rotation and $(g_y^2R^2)^2 = 1$.

Observe that $g_y R(x,y) = (-y+1/2, -x+1/2)$, so Rg_y is a reflection whose reflection axis is in the direction of $\boldsymbol{e}_y - \boldsymbol{e}_x$, thus we have $(g_y R)^2 = 1$.

There is one class of four-fold rotation centers $C_s(C)$, one class of two-fold rotation centers $C_s(R^2 g_y^2)$ and one class of reflections $C_s(g_y C)$, as shown in Fig. 15. The presentation is given by

$$P4g = \langle g_y, R | (g_y R)^2, (g_y^2 R^2)^2, R^4 \rangle. \tag{107}$$

When we consider projective algebra, the relations of presentation Eq. (107) become

$$\mathsf{R}^4 = \alpha_1, \tag{108a}$$
$$(\mathsf{g}_y^2 \mathsf{R}^2)^2 = \alpha_2, \tag{108b}$$
$$(\mathsf{g}_y \mathsf{R})^2 = \beta_1. \tag{108c}$$

To get the restriction of self-consistency condition, we first conjugate $\mathsf{g}_y^2\mathsf{R}^2$ by $\mathsf{g}_y\mathsf{R}$

$$\begin{aligned} & (\mathsf{g}_y\mathsf{R})(\mathsf{g}_y^2\mathsf{R}^2)(\mathsf{g}_y\mathsf{R})^{-1} = \beta_1(\mathsf{g}_y\mathsf{R})^{-1}(\mathsf{g}_y^2\mathsf{R}^2)(\mathsf{g}_y\mathsf{R})^{-1} \\ = \; & \beta_1(\mathsf{R}^{-1}\mathsf{g}_y^{-1})(\mathsf{g}_y\mathsf{g}_y\mathsf{R}\mathsf{R})(\mathsf{R}^{-1}\mathsf{g}_y^{-1}) = \alpha_3\mathsf{R}^{-1}(\mathsf{g}_y\mathsf{R})\mathsf{g}_y^{-1} \\ = \; & \beta_1^2\mathsf{R}^{-1}(\mathsf{g}_y\mathsf{R})^{-1}\mathsf{g}_y^{-1} = \beta_1^2\mathsf{R}^{-2}\mathsf{g}_y^{-2} = \beta_1^2(\mathsf{g}_y^2\mathsf{R}^2)^{-1}. \end{aligned}$$

Then we conjugate Eq.(108)(b) by $\mathsf{g}_y\mathsf{R}$,

$$\alpha_2 = (\mathsf{g}_y\mathsf{R})(\mathsf{g}_y^2\mathsf{R}^2)^2(\mathsf{g}_y\mathsf{R})^{-1} = \beta_1^4(\mathsf{g}_y^2\mathsf{R}^2)^{-2} = \beta_1^4\alpha_2^{-1}. \tag{109}$$

Thus α_2, β_1 satisfy

$$\beta_1^4\alpha_2^{-2} = 1. \tag{110}$$

It has two solutions

$$\alpha_2 = \pm\beta_1 \equiv \alpha\beta_1^2. \tag{111}$$

For $U(1)$ extension, factors α_1, β_1 are trivial because we can cancel them by redefining $\mathsf{R} \to \mathsf{R}' = \alpha_1^{-1/4}\mathsf{R}, \mathsf{g}_y \to \mathsf{g}'_y = \beta_1^{-1/2}\mathsf{g}_y$. There is only one independent cohomology invariant $\alpha = \alpha_2\beta_1^{-2} \in \mathbb{Z}_2$.

For $\mathbb{Z}_2$ extension, we have three independent cohomology invariants $\alpha_1, \alpha_2, \beta_1 \in \mathbb{Z}_2$, corresponding to conjugacy classes of rotations and reflections respectively, as shown in Fig. 14.

These results are in agreement with that of the group cohomology

$$H^2(P4g, U(1)) = \mathbb{Z}_2, \tag{112a}$$
$$H^2(P4g, \mathbb{Z}_2) = \mathbb{Z}_2^3. \tag{112b}$$

n. P3

In the group $P3$, the angle between two translation L_a and L_b is $\pi/3$. The rotation operator R rotates translations as $RL_aR^{-1} = L_a^{-1}L_b, RL_bR^{-1} = L_a^{-1}$. The presentation is given by

$$\begin{aligned} P3 = \; & \langle L_a, L_b, R | [L_a, L_b], RL_aR^{-1} = L_a^{-1}L_b, \\ & RL_bR^{-1} = L_a^{-1}, R^3 \rangle. \end{aligned} \tag{113}$$

When we consider projective algebra, the relations in Eq. (113) are modified as

$$\begin{aligned} & \mathsf{L}_a\mathsf{L}_b\mathsf{L}_a^{-1}\mathsf{L}_b^{-1} = \sigma, \\ & \mathsf{R}\mathsf{L}_a\mathsf{R}^{-1} = \eta_1\mathsf{L}_a^{-1}\mathsf{L}_b, \\ & \mathsf{R}\mathsf{L}_b\mathsf{R}^{-1} = \eta_2\mathsf{L}_a^{-1}, \\ & \mathsf{R}^3 = \alpha. \end{aligned}$$

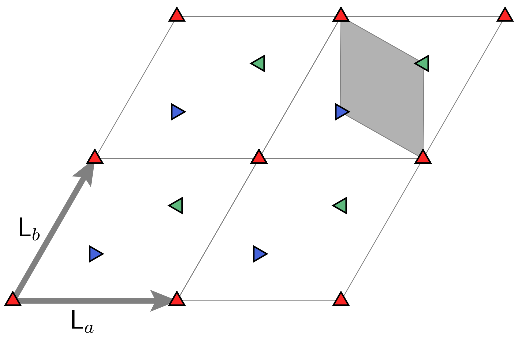

Supplementary Fig.16: Cell structure for $P3$. There are three different conjugacy classes of three-fold rotation centers. The gray shaded region is the fundamental domain of the group.

For $\mathbb{Z}_2$ extension, η_1, η_2 can be trivialized by redefining $\mathsf{L}_a \to \mathsf{L}'_a = (\eta_1\eta_2)\mathsf{L}_a$, $\mathsf{L}_b \to \mathsf{L}'_b = \eta_1\mathsf{L}_b$. For $U(1)$ extension, η_1, η_2 can also be trivialized by redefining $\mathsf{L}_a \to \mathsf{L}'_a = (\eta_1\eta_2)^{-1/3}\mathsf{L}_a$, $\mathsf{L}_b \to \mathsf{L}'_b = (\eta_1^{-1}\eta_2^2)^{-1/3}\mathsf{L}_b$. Because when n is odd, factor system of C_n is always trivial for both $U(1)$ and $\mathbb{Z}_2$ extension, so α can also be trivialized. We only have one independent cohomology invariant σ for the translation subgroups. Finally, the projective algebra is

$$\mathsf{L}_a\mathsf{L}_b\mathsf{L}_a^{-1}\mathsf{L}_b^{-1} = \sigma, \tag{114a}$$
$$\mathsf{R}\mathsf{L}_a\mathsf{R}^{-1} = \mathsf{L}_a^{-1}\mathsf{L}_b, \tag{114b}$$
$$\mathsf{R}\mathsf{L}_b\mathsf{R}^{-1} = \mathsf{L}_a^{-1}, \tag{114c}$$
$$\mathsf{R}^3 = 1, \tag{114d}$$

where $\sigma \in \mathbb{Z}_2$ for $\mathbb{Z}_2$ extension and $\sigma \in U(1)$ for $U(1)$ extension. These results are in agreement with the group cohomology

$$H^2(P3, \mathbb{Z}_2) = \mathbb{Z}_2, \tag{115a}$$
$$H^2(P3, U(1)) = U(1). \tag{115b}$$

o. P3m1

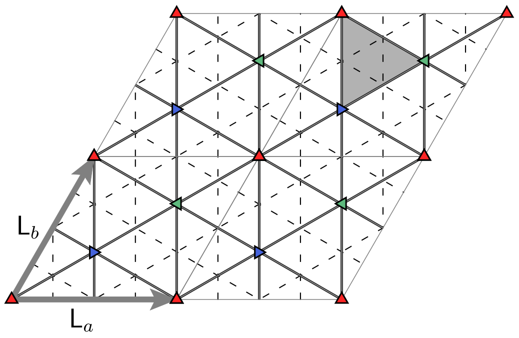

Supplementary Fig.17: Cell structure of $P3m1$. The reflection axes pass through three different rotation centers. The gray shaded region is the fundamental domain of the group.

As shown in Fig. 17, the group $P3m1$ contains reflections whose axes pass through three different rotation centers of $P3$. Here we choose the reflection M whose axis is perpendicular to $\boldsymbol{e}_a$ as one generator. M reverses translation L_a and rotation R. The presentation is given by

$$\begin{aligned} P3m1 = \quad & \langle L_a, L_b, R, M | [L_a, L_b], RL_aR^{-1} = L_a^{-1}L_b, \\ & RL_bR^{-1} = L_a^{-1}, ML_aM^{-1} = L_a^{-1}, \\ & MRM^{-1} = R^{-1}, M^2, R^3\rangle. \end{aligned} \tag{116}$$

When we consider projective algebra, we need to modify the relations in Eq. (116).

For both $\mathbb{Z}_2$ and $U(1)$ extension, we can first trivialize the factor system between rotation and translations as we proved in group $P3$ and trivialize the factor system of point group D_3.

We proceed to look at the requirement of self-consistency condition on the factor system between M and L_a. Suppose

$$\mathsf{M}\mathsf{L}_a\mathsf{M}^{-1} = \eta \mathsf{L}_a^{-1}.$$

Then we take relations between rotation and translations

$$\mathsf{R}\mathsf{L}_a\mathsf{R}^{-1} = \mathsf{L}_a^{-1}\mathsf{L}_b,$$
$$\mathsf{R}\mathsf{L}_b\mathsf{R}^{-1} = \mathsf{L}_a^{-1},$$

conjugate them by M, and use relations $\mathsf{M}\mathsf{L}_a\mathsf{M}^{-1} = \eta\mathsf{L}_a^{-1}$ and $\mathsf{M}\mathsf{R}\mathsf{M}^{-1} = \mathsf{R}^{-1}$, we get

$$\eta\mathsf{R}^{-1}\mathsf{L}_a^{-1}\mathsf{R} = \eta^{-1}\mathsf{L}_a\mathsf{M}\mathsf{L}_b\mathsf{M}^{-1},$$
$$\mathsf{R}^{-1}\mathsf{M}\mathsf{L}_b\mathsf{M}^{-1}\mathsf{R} = \eta^{-1}\mathsf{L}_a.$$

By cancelling $\mathsf{M}\mathsf{L}_b\mathsf{M}^{-1}$ from above equations, we get

$$\eta\mathsf{R}^{-1}\mathsf{L}_a^{-1}\mathsf{R} = \eta^{-2}\mathsf{L}_a(\mathsf{R}\mathsf{L}_a\mathsf{R}^{-1}).$$

With $\mathsf{R}\mathsf{L}_a\mathsf{R}^{-1} = \mathsf{L}_a^{-1}\mathsf{L}_b$ and $\mathsf{R}^{-1}\mathsf{L}_a^{-1}\mathsf{R} = \mathsf{L}_b$, we have

$$\eta\mathsf{L}_b = \eta^{-2}\mathsf{L}_b.$$

So factor η satisfies $\eta^3 = 1$. For $\mathbb{Z}_2$ extension, $\eta = 1$, while for $U(1)$ extension, $\eta = e^{i\theta}, \theta = 0, 2\pi/3, 4\pi/3$. But η can be trivialized by redefining $\mathsf{L}_a \to \mathsf{L}'_a = e^{i\xi}\mathsf{L}_a, \mathsf{L}_b \to \mathsf{L}'_b = e^{-i\xi}\mathsf{L}_a, \xi = 0, 4\pi/3, 2\pi/3$ respectively, and the redefining does not influence the original trivialization of factor system between translations and rotation. Hence, there are no nontrivial factor between M and L_a for both $\mathbb{Z}_2$ and $U(1)$ extension.

Finally, projective relations of presentation are

$$\mathsf{L}_a\mathsf{L}_b\mathsf{L}_a^{-1}\mathsf{L}_b^{-1} = \sigma, \tag{117a}$$
$$\mathsf{R}\mathsf{L}_a\mathsf{R}^{-1} = \mathsf{L}_a^{-1}\mathsf{L}_b, \tag{117b}$$
$$\mathsf{R}\mathsf{L}_b\mathsf{R}^{-1} = \mathsf{L}_a^{-1}, \tag{117c}$$
$$\mathsf{R}^3 = 1, \tag{117d}$$
$$\mathsf{M}\mathsf{R}\mathsf{M}^{-1} = \mathsf{R}^{-1}, \tag{117e}$$
$$\mathsf{M}\mathsf{L}_a\mathsf{M}^{-1} = \mathsf{L}_a^{-1}, \tag{117f}$$
$$\mathsf{M}^2 = \beta. \tag{117g}$$

For $\mathbb{Z}_2$ extension we have two independent cohomology invariants $\sigma, \beta \in \mathbb{Z}_2$, while for $U(1)$ extension we have only one independent cohomology invariantr $\sigma \in \mathbb{Z}_2$.

Above results are in agreement with the group cohomology

$$H^2(P3m1, \mathbb{Z}_2) = \mathbb{Z}_2^2, \tag{118a}$$
$$H^2(P3m1, U(1)) = \mathbb{Z}_2. \tag{118b}$$

p. P31m

As shown in Fig. 18, the group $P31m$ contains reflections whose axes pass through only one class of rotation centers. Here we choose reflection M whose axis is parallel to $\boldsymbol{e}_a$ as one generator. M preserves translation L_a and reverses rotation R. Thus, The presentation is given by

$$\begin{aligned} P31m = \quad & \langle L_a, L_b, R, M | [L_a, L_b], RL_aR^{-1} = L_a^{-1}L_b, \\ & RL_bR^{-1} = L_a^{-1}, ML_aM^{-1} = L_a \\ & MRM = R^{-1}, M^2, R^3\rangle. \end{aligned} \tag{119}$$

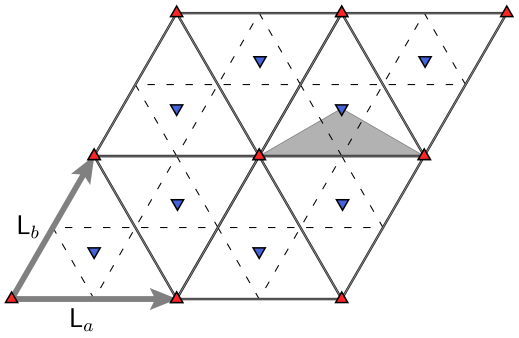

Supplementary Fig.18: Cell structure of $P31m$. Reflection axes pass through only one class of rotation centers. The gray shaded region is the fundamental domain of the group.

When we consider projective algebra, we need to modify the relations in Eq. (119).

For both $\mathbb{Z}_2$ and $U(1)$ extension, we can first trivialize the factor system between rotation and translations as we proved in group $P3$ and trivialize the factor system of point group D_3.

We proceed to look at the requirement of self-consistency condition on the factor system between M and L_a. Suppose

$$\mathsf{M}\mathsf{L}_a\mathsf{M}^{-1} = \eta\mathsf{L}_a.$$

Then we take relations between rotation and translations

$$\mathsf{R}\mathsf{L}_a\mathsf{R}^{-1} = \mathsf{L}_a^{-1}\mathsf{L}_b,$$
$$\mathsf{R}\mathsf{L}_b\mathsf{R}^{-1} = \mathsf{L}_a^{-1},$$

conjugate them by M, and use relations $\mathsf{M}\mathsf{L}_a\mathsf{M}^{-1} = \eta\mathsf{L}_a$ and $\mathsf{M}\mathsf{R}\mathsf{M}^{-1} = \mathsf{R}^{-1}$, we get

$$\eta\mathsf{R}^{-1}\mathsf{L}_a\mathsf{R} = \eta^{-1}\mathsf{L}_a^{-1}\mathsf{M}\mathsf{L}_b\mathsf{M}^{-1},$$
$$\mathsf{R}^{-1}\mathsf{M}\mathsf{L}_b\mathsf{M}^{-1}\mathsf{R} = \eta^{-1}\mathsf{L}_a^{-1}.$$

By cancelling $\mathsf{M}\mathsf{L}_b\mathsf{M}^{-1}$ from above equations, we get

$$\eta\mathsf{R}^{-1}\mathsf{L}_a\mathsf{R} = \eta^{-2}\mathsf{L}_a^{-1}(\mathsf{R}\mathsf{L}_a^{-1}\mathsf{R}^{-1}).$$

With $\mathsf{R}\mathsf{L}_a^{-1}\mathsf{R}^{-1} = \mathsf{L}_b^{-1}\mathsf{L}_a$ and $\mathsf{R}^{-1}\mathsf{L}_a\mathsf{R} = \mathsf{L}_b^{-1}$, we have

$$\eta\mathsf{L}_b^{-1} = \eta^{-2}\mathsf{L}_a^{-1}\mathsf{L}_b^{-1}\mathsf{L}_a = \eta^{-2}\sigma\mathsf{L}_b^{-1}. \tag{120}$$

Thus we have $\eta^3 = \sigma$. Futhermore, reflection symmetry requires $\eta, \sigma \in \mathbb{Z}_2$, so the only possible solution is $\eta = \sigma$ for both $\mathbb{Z}_2$ and $U(1)$ extension.

Finally, the projective relations of presentation are

$$\mathsf{L}_a\mathsf{L}_b\mathsf{L}_a^{-1}\mathsf{L}_b^{-1} = \sigma, \tag{121a}$$
$$\mathsf{R}\mathsf{L}_a\mathsf{R}^{-1} = \mathsf{L}_a^{-1}\mathsf{L}_b, \tag{121b}$$
$$\mathsf{R}\mathsf{L}_b\mathsf{R}^{-1} = \mathsf{L}_a^{-1}, \tag{121c}$$
$$\mathsf{R}^3 = 1, \tag{121d}$$
$$\mathsf{M}\mathsf{R}\mathsf{M}^{-1} = \mathsf{R}^{-1}, \tag{121e}$$
$$\mathsf{M}\mathsf{L}_a\mathsf{M}^{-1} = \sigma\mathsf{L}_a, \tag{121f}$$
$$\mathsf{M}^2 = \beta. \tag{121g}$$

For $\mathbb{Z}_2$ extension we have two independent cohomology invariants σ, β $\sigma \in \mathbb{Z}_2$.

These results are in agreement with that of the group cohomology

$$H^2(P31m, \mathbb{Z}_2) = \mathbb{Z}_2^2, \tag{122a}$$
$$H^2(P31m, U(1)) = \mathbb{Z}_2. \tag{122b}$$

q. P6

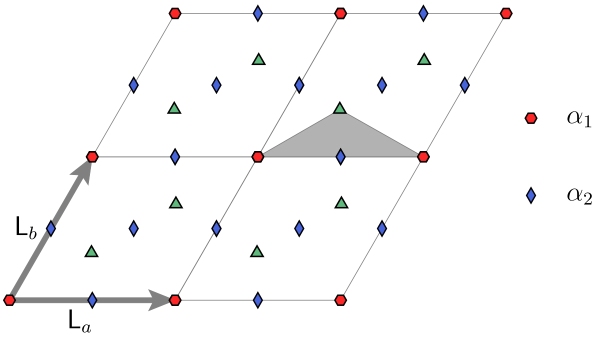

Supplementary Fig.19: Cell structure of $P6$. There is one conjugacy class of two-fold, three-fold and six-fold rotation centers respectively. The gray shaded region is the fundamental domain of the group.

In the group $P6$, the angle between two translation L_a and L_b is $\pi/3$, the rotation operator R rotates translations as $RL_aR^{-1} = L_b, RL_bR^{-1} = L_a^{-1}L_b$. The presentation is given by

$$\begin{aligned} P6 = \ & \langle L_a, L_b, R|[L_a, L_b], RL_aR^{-1} = L_b, \\ & RL_bR^{-1} = L_a^{-1}L_b, R^6\rangle. \end{aligned} \tag{123}$$

Since L_b can be expressed in terms of L_a and R, we can choose L_a and R as independent generators and the presentation can also be given by

$$P6 = \langle L_a, R|(L_aR^2)^3, (L_aR^3)^2, R^6\rangle. \tag{124}$$

When we consider projective algebra, we need to modify the relations in Eq. (123). For the relations between rotation and translations, we have

$$\begin{aligned} \mathsf{R}\mathsf{L}_a\mathsf{R}^{-1} &= \eta_1\mathsf{L}_b, \\ \mathsf{R}\mathsf{L}_b\mathsf{R}^{-1} &= \eta_2\mathsf{L}_a^{-1}\mathsf{L}_b. \end{aligned}$$

The factors η_1, η_2 can be trivialized by $\mathsf{L}_a \to \mathsf{L}'_a = \eta_2^{-1}\mathsf{L}_a$, $\mathsf{L}_b \to \mathsf{L}'_b = (\eta_1^{-1}\eta_2)\mathsf{L}_b$. Thus projective relations of presentation are

$$\mathsf{L}_a\mathsf{L}_b\mathsf{L}_a^{-1}\mathsf{L}_b^{-1} = \sigma, \tag{125a}$$
$$\mathsf{R}\mathsf{L}_a\mathsf{R}^{-1} = \mathsf{L}_b, \tag{125b}$$
$$\mathsf{R}\mathsf{L}_b\mathsf{R}^{-1} = \mathsf{L}_a^{-1}\mathsf{L}_b, \tag{125c}$$
$$\mathsf{R}^6 = \alpha. \tag{125d}$$

For $\mathbb{Z}_2$ extension, we have two independent cohomology invariants $\sigma, \alpha \in \mathbb{Z}_2$, while for $U(1)$ extension we have only one cohomology invariant $\sigma \in U(1)$ since α can be trivialized.

These results are consistent with that of the group cohomology

$$H^2(P6, \mathbb{Z}_2) = U(1), \tag{126a}$$
$$H^2(P6, U(1)) = \mathbb{Z}_2^2. \tag{126b}$$

For $\mathbb{Z}_2$ extension, we can also obtain the projective algebra by modifying the relations in Eq. (124) as

$$(\mathsf{L}_a\mathsf{R}^2)^3 = 1, \tag{127a}$$
$$\mathsf{R}^6 = \alpha \equiv \alpha_1, \tag{127b}$$
$$(\mathsf{L}_a\mathsf{R}^3)^2 = \sigma\alpha_r \equiv \alpha_2. \tag{127c}$$

These relations mean that the conjugacy class of six-fold rotation centers $C_s(R)$ and two-fold rotation centers $C_s(L_aR^3)$ has an independent cohomology invariant respectively, as shown in Fig. 19.

r. P6m

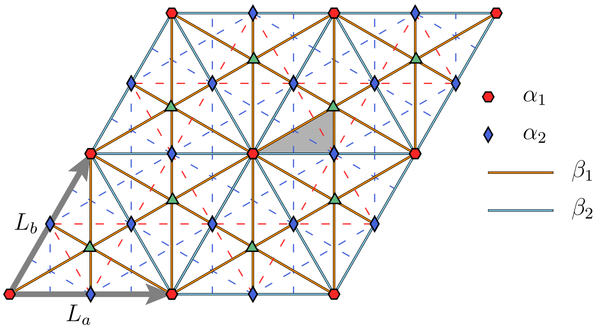

Supplementary Fig.20: Cell structure of $P6m$. There are two conjugacy classes of rotation and two conjugacy classes of reflection. The gray shaded region is the fundamental domain of the group.

The group $P6m$ is obtained by adding reflection symmetry to group $P6$. Here we choose the reflection M whose axis is perpendicular to $\boldsymbol{e}_a$ as a generator. M reverses translation L_a and rotations R. The presentation is given by

$$\begin{aligned} P6m &= \langle L_a, L_b, R, M | [L_a, L_b], RL_aR^{-1} = L_b, \\ & RL_bR^{-1} = L_a^{-1}L_b, ML_aM^{-1} = L_a^{-1}, \\ & MRM^{-1} = R^{-1}, M^2, R^6 \rangle. \end{aligned} \tag{128}$$

The group can also be presented in terms of rotations and reflections as

$$\begin{aligned} P6m &= \langle L_a, R, M | (L_aR^2)^3, (L_aR^3)^2, R^6, M^2, \\ & (RM)^2, (L_aM)^2 \rangle. \end{aligned} \tag{129}$$

When we consider projective algebra, we need to modify the relations in Eq. (128).

For both $\mathbb{Z}_2$ and $U(1)$ extension, we can first trivialize the factor system between rotation and translations as we proved in group $P6$, and label the factor system of subgroup D_6 by cohomology invariants $\alpha_r, \alpha_m, \alpha_{rm}$.

We proceed to look at the requirement of self-consistency condition on the factor system between M and L_a. Suppose

$$\mathsf{M}\mathsf{L}_a\mathsf{M}^{-1} = \eta \mathsf{L}_a^{-1}.$$

Then we take relations between rotation and translations

$$\begin{aligned} \mathsf{R}\mathsf{L}_a\mathsf{R}^{-1} &= \mathsf{L}_b, \\ \mathsf{R}\mathsf{L}_b\mathsf{R}^{-1} &= \mathsf{L}_a^{-1}\mathsf{L}_b, \end{aligned}$$

conjugate them by M, and use relations $\mathsf{M}\mathsf{L}_a\mathsf{M}^{-1} = \eta\mathsf{L}_a^{-1}$ and $\mathsf{M}\mathsf{R}\mathsf{M}^{-1} = \mathsf{R}^{-1}$, we get

$$\begin{aligned} \eta\mathsf{R}^{-1}\mathsf{L}_a^{-1}\mathsf{R} &= \mathsf{M}_x\mathsf{L}_b\mathsf{M}_x^{-1}, \\ \mathsf{R}^{-1}\mathsf{M}_x\mathsf{L}_b\mathsf{M}_x^{-1}\mathsf{R} &= \eta^{-1}\mathsf{L}_a\mathsf{M}_x\mathsf{L}_b\mathsf{M}_x^{-1}. \end{aligned}$$

By cancelling $\mathsf{M}\mathsf{L}_b\mathsf{M}^{-1}$ from above equations, we get

$$\mathsf{R}^{-1}(\mathsf{R}^{-1}\mathsf{L}_a^{-1}\mathsf{R})\mathsf{R} = \eta^{-1}\mathsf{L}_a(\mathsf{R}^{-1}\mathsf{L}_a^{-1}\mathsf{R}).$$

Conjugate above equation with R^2, we have

$$\mathsf{L}_a^{-1} = \eta^{-1}(\mathsf{R}^2\mathsf{L}_a\mathsf{R}^{-2})(\mathsf{R}\mathsf{L}_a^{-1}\mathsf{R}^{-1}).$$

Use $\mathsf{R}^2\mathsf{L}_a\mathsf{R}^{-2} = \mathsf{L}_a^{-1}\mathsf{L}_b$ and $\mathsf{R}\mathsf{L}_a^{-1}\mathsf{R}^{-1} = \mathsf{L}_b^{-1}\mathsf{L}_b$, we obtain

$$\mathsf{L}_a^{-1} = \eta^{-1}\mathsf{L}_a^{-1}.$$

So $\eta = 1$, i,e., η is trivial.

Thus, the projective relations of presentation of $P6m$ are

$$\mathsf{L}_a\mathsf{L}_b\mathsf{L}_a^{-1}\mathsf{L}_b^{-1} = \sigma, \quad (130a)$$
$$\mathsf{R}\mathsf{L}_b\mathsf{R}^{-1} = \mathsf{L}_a^{-1}\mathsf{L}_b, \quad (130b)$$
$$\mathsf{R}\mathsf{L}_a\mathsf{R}^{-1} = \mathsf{L}_b, \quad (130c)$$
$$\mathsf{M}\mathsf{L}_a\mathsf{M}^{-1} = \mathsf{L}_a^{-1}, \quad (130d)$$
$$\mathsf{R}^6 = \alpha_r, \quad (130e)$$
$$\mathsf{M}\mathsf{R}\mathsf{M}^{-1} = \alpha_{rm}\mathsf{R}^{-1}, \quad (130f)$$
$$\mathsf{M}^2 = \alpha_m. \quad (130g)$$

For $\mathbb{Z}_2$ extension, we have four independent cohomology invariants $\sigma, \alpha_r, \alpha_{rm}, \alpha_m \in \mathbb{Z}_2$, while for $U(1)$ extension we have two independent cohomology invariants $\sigma, \alpha = \alpha_r\alpha_{rm}^{-3} \in \mathbb{Z}_2$, since α_{rm}, α_m are trivial as we analyzed in group D_n.

These results are in agreement with that of the group cohomology

$$H^2(P6m, \mathbb{Z}_2) = \mathbb{Z}_2^4, \quad (131a)$$
$$H^2(P6m, U(1)) = \mathbb{Z}_2^2. \quad (131b)$$

For $\mathbb{Z}_2$ extension,we can also obtain the projective algebra by modifying the relations in Eq. (129) as

$$(\mathsf{L}_a\mathsf{R}^2)^3 = 1, \quad (132a)$$
$$\mathsf{R}^6 = \alpha_r \equiv \alpha_1, \quad (132b)$$
$$(\mathsf{L}_a\mathsf{R}^3)^2 = \sigma\alpha_r \equiv \alpha_2, \quad (132c)$$
$$\mathsf{M}^2 = \alpha_m \equiv \beta_1, \quad (132d)$$
$$(\mathsf{L}_a\mathsf{M})^2 = \alpha_m = \beta_1, \quad (132e)$$
$$(\mathsf{R}\mathsf{M})^2 = \alpha_{rm}\alpha_m \equiv \beta_2. \quad (132f)$$

The meaning of these relations are illustrated in Fig. 20.

s. Table of relations and cohomology invariants

G	$H^2(G,\mathbb{Z}_2)$	Generators	Cohomology invariants	N_G
$P1$	$\mathbb{Z}_2$	$\mathsf{L}_a, \mathsf{L}_b$	$\sigma = \mathsf{L}_x\mathsf{L}_y\mathsf{L}_x^{-1}\mathsf{L}_y^{-1}$.	2
$P2$	$\mathbb{Z}_2^4$	$\mathsf{L}_a, \mathsf{L}_b, \mathsf{R}$	$\alpha_1 = \mathsf{R}^2, \quad \alpha_2 = (\mathsf{L}_a\mathsf{R})^2, \quad \alpha_3 = (\mathsf{L}_b\mathsf{R})^2, \quad \alpha_4 = (\mathsf{L}_a\mathsf{L}_b\mathsf{R})^2$.	5
Pm	$\mathbb{Z}_2^4$	$\mathsf{L}_x, \mathsf{L}_y, \mathsf{M}_x$	$\beta_1 = \mathsf{M}_x^2, \quad \beta_2 = (\mathsf{L}_x\mathsf{M}_x)^2, \quad \eta_1 = \mathsf{M}_x\mathsf{L}_y\mathsf{M}_x^{-1}\mathsf{L}_y^{-1}, \quad \eta_2 = (\mathsf{L}_x\mathsf{M}_x)\mathsf{L}_y(\mathsf{L}_x\mathsf{M}_x)^{-1}\mathsf{L}_y^{-1}$.	10
Pg	$\mathbb{Z}_2$	$\mathsf{L}_x, \mathsf{g}_x$	$\tau = \mathsf{g}_x\mathsf{L}_x\mathsf{g}_x^{-1}\mathsf{L}_x$.	2
Cm	$\mathbb{Z}_2^2$	$\mathsf{L}_a, \mathsf{L}_b, \mathsf{M}$	$\sigma = \mathsf{L}_a\mathsf{L}_b\mathsf{L}_a^{-1}\mathsf{L}_b^{-1}, \quad \beta = \mathsf{M}^2, \quad 1 = \mathsf{M}\mathsf{L}_a\mathsf{M}^{-1}\mathsf{L}_b^{-1}$.	4
Pmm	$\mathbb{Z}_2^8$	$\mathsf{L}_x, \mathsf{L}_y, \mathsf{M}_x, \mathsf{M}_y$	$\alpha_1 = (\mathsf{M}_x\mathsf{M}_y)^2, \quad \alpha_2 = (\mathsf{L}_x\mathsf{M}_x\mathsf{M}_y)^2, \quad \alpha_3 = (\mathsf{L}_y\mathsf{M}_x\mathsf{M}_y)^2, \quad \alpha_4 = (\mathsf{L}_x\mathsf{L}_y\mathsf{M}_x\mathsf{M}_y)^2,$<br>$\beta_1 = \mathsf{M}_x^2, \quad \beta_2 = (\mathsf{L}_x\mathsf{M}_x)^2, \quad \beta_3 = \mathsf{M}_y^2, \quad \beta_4 = (\mathsf{L}_y\mathsf{M}_y)^2$.	51
Pmg	$\mathbb{Z}_2^4$	$\mathsf{g}_y, \mathsf{L}_y, \mathsf{M}_x$	$\alpha_1 = (\mathsf{M}_x\mathsf{g}_y)^2, \quad \alpha_2 = (\mathsf{L}_y\mathsf{M}_x\mathsf{g}_y)^2, \quad \beta_1 = \mathsf{M}_x^2, \quad \eta = \mathsf{M}_x\mathsf{L}_y\mathsf{M}_x^{-1}\mathsf{L}_y^{-1}$.	12
Pgg	$\mathbb{Z}_2^2$	$\mathsf{g}_x, \mathsf{g}_y$	$\alpha_1 = (\mathsf{g}_x\mathsf{g}_y)^2, \quad \alpha_2 = (\mathsf{g}_x\mathsf{g}_y^{-1})^2$.	3
Cmm	$\mathbb{Z}_2^5$	$\mathsf{L}_a, \mathsf{M}_x, \mathsf{M}_y$	$\alpha_1 = (\mathsf{M}_x\mathsf{M}_y)^2, \quad \alpha_2 = (\mathsf{L}_a\mathsf{M}_x\mathsf{M}_y)^2, \quad \alpha_3 = (\mathsf{L}_a\mathsf{M}_x\mathsf{L}_a^{-1}\mathsf{M}_y)^2, \quad \beta_1 = \mathsf{M}_x^2, \quad \beta_2 = \mathsf{M}_y^2$.	18
$P4$	$\mathbb{Z}_2^3$	L_x, R	$\alpha_1 = \mathsf{R}^4, \quad \alpha_2 = (\mathsf{L}_x\mathsf{R}^2)^2, \quad \alpha_3 = (\mathsf{L}_x\mathsf{R})^4$.	6
$P4m$	$\mathbb{Z}_2^6$	$\mathsf{L}_x, \mathsf{R}, \mathsf{M}$	$\alpha_1 = \mathsf{R}^4, \quad \alpha_2 = (\mathsf{L}_x\mathsf{R}^2)^2, \quad \alpha_3 = (\mathsf{L}_x\mathsf{R})^4, \quad \beta_1 = \mathsf{M}^2, \quad \beta_2 = (\mathsf{R}\mathsf{M})^2, \quad \beta_3 = (\mathsf{L}_x\mathsf{M})^2$.	40
$P4g$	$\mathbb{Z}_2^3$	g_y, R	$\alpha_1 = \mathsf{R}^4, \quad \alpha_2 = (\mathsf{g}_y^2\mathsf{R}^2)^2, \quad \beta_1 = (\mathsf{g}_y\mathsf{R})^2$.	6
$P3$	$\mathbb{Z}_2$	$\mathsf{L}_a, \mathsf{L}_b, \mathsf{R}$	$\sigma = \mathsf{L}_a\mathsf{L}_b\mathsf{L}_a^{-1}\mathsf{L}_b^{-1}, \quad 1 = \mathsf{R}\mathsf{L}_a\mathsf{R}^{-1}\mathsf{L}_b^{-1}\mathsf{L}_a, \quad 1 = \mathsf{R}\mathsf{L}_b\mathsf{R}^{-1}\mathsf{L}_a, \quad 1 = \mathsf{R}^3$.	2
$P3m1$	$\mathbb{Z}_2^2$	$\mathsf{L}_a, \mathsf{L}_b, \mathsf{R}, \mathsf{M}$	$\sigma = \mathsf{L}_a\mathsf{L}_b\mathsf{L}_a^{-1}\mathsf{L}_b^{-1}, \quad \beta = \mathsf{M}^2 = (\mathsf{R}\mathsf{M})^2 = (\mathsf{L}_a\mathsf{M})^2, \quad 1 = \mathsf{R}\mathsf{L}_a\mathsf{R}^{-1}\mathsf{L}_b^{-1}\mathsf{L}_a,$<br>$1 = \mathsf{R}\mathsf{L}_b\mathsf{R}^{-1}\mathsf{L}_a, \quad 1 = \mathsf{R}^3$.	4
$P31m$	$\mathbb{Z}_2^2$	$\mathsf{L}_a, \mathsf{L}_b, \mathsf{R}, \mathsf{M}$	$\sigma = \mathsf{L}_a\mathsf{L}_b\mathsf{L}_a^{-1}\mathsf{L}_b^{-1} = \mathsf{L}_a\mathsf{M}\mathsf{L}_a^{-1}\mathsf{M}^{-1}, \quad \beta = \mathsf{M}^2 = (\mathsf{R}\mathsf{M})^2, \quad 1 = \mathsf{R}\mathsf{L}_a\mathsf{R}^{-1}\mathsf{L}_b^{-1}\mathsf{L}_a,$<br>$1 = \mathsf{R}\mathsf{L}_b\mathsf{R}^{-1}\mathsf{L}_a, \quad 1 = \mathsf{R}^3$.	4
$P6$	$\mathbb{Z}_2^2$	L_a, R	$\alpha_1 = \mathsf{R}^6, \quad \alpha_2 = (\mathsf{L}_a\mathsf{R}^3)^2, \quad 1 = (\mathsf{L}_a\mathsf{R}^2)^3$.	4
$P6m$	$\mathbb{Z}_2^4$	$\mathsf{L}_a, \mathsf{R}, \mathsf{M}$	$\alpha_1 = \mathsf{R}^6, \quad \alpha_2 = (\mathsf{L}_a\mathsf{R}^3)^2, \quad \beta_1 = \mathsf{M}^2 = (\mathsf{L}_a\mathsf{M})^2, \quad \beta_2 = (\mathsf{R}\mathsf{M})^2, \quad 1 = (\mathsf{L}_a\mathsf{R}^2)^3$.	16

Supplementary Table.I: $\mathbb{Z}_2$ cohomology invariants for all wallpaper groups.

Supplementary Note 3. The construction of the canonical models from the cohomology invariants

A complete set of the cohomology invariants for all 17 wallpaper groups have been constructed in the previous section. In this section, we further present general procedures to translate these cohomology invariants into lattice models with appropriate flux configurations. Following the general procedure for model construction, we can in the end arrive at 17 canonical lattice models, i.e., for each wallpaper group we construct a canonical model that can realize all possible values of cohomology invariants and therefore all cohomology classes of multipliers.

a. Flux interpretation of cohomology invariants

In this subsection, we provide technical details for how each type of cohomology invariant can be interpreted as certain flux configurations on the appropriate lattice structure.

i. Crystal Symmetries with Gauge Fields

First of all, we present a formalism of crystal symmetries on a lattice with gauge fields.

In gauge field theory, gauge flux configurations are gauge invariant. A given gauge flux configuration can be described by many equivalent gauge connection configurations, which differ from each other by gauge transformations. In lattice systems, a gauge transformation G simply multiplies a phase on each lattice site, and therefore can be regarded as a diagonal matrix indexed by the lattice sites.

Now, suppose we have a lattice with the gauge field. A spatial symmetry transformation R that preserves the lattice and flux configuration in general changes the connection configuration. The transformed connection configuration must be related to the original one by a gauge transformation G_R, since they correspond to the same flux configuration.

Thus, the symmetry operator should be modified as

$$\mathsf{R} = \mathsf{G}_R R, \tag{133}$$

which is a combination of the pure spatial operation R and the gauge transformation G_R.

We now consider how the physical operator R acts on a tight-binding model, $H = \sum_{ij} t_{ij} a_i^\dagger a_j$. The action of R on H is given by

$$\begin{aligned} H' &= \sum_{ij} t_{ij} \mathsf{G}_R(R(i)) \mathsf{G}_R^*(R(j)) a^\dagger_{R(i)} a_{R(j)} \\ &= \sum_{ij} \mathsf{G}_R(i)\mathsf{G}_R^*(j) t_{R^{-1}(i)R^{-1}(j)} a_i^\dagger a_j, \end{aligned} \tag{134}$$

where $G_R(i)$ is the phase of gauge tansformation on site i, and $R(i)$ is the site transformed from i by R. Thus, the invariance under R means

$$t_{ij} = \mathsf{G}_R(i)\mathsf{G}_R^*(j) t_{R^{-1}(i)R^{-1}(j)}. \tag{135}$$

ii. The cohomology invariant of the translation subgroups

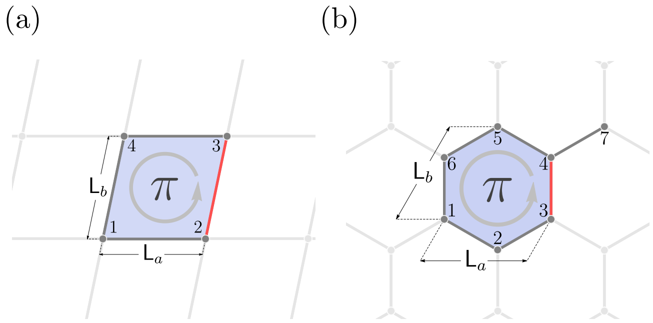

Supplementary Fig.21: The Flux determines the cohomology invariant of translation subgroup. (a) A rectangle plaquette spanned by L_x and L_y in two directions. (b) A hexagon plaquette spanned by L_a and L_b in two directions.

The factor system of the translation subgroup is determined by the cohomology invariant $\sigma = \mathsf{L}_a\mathsf{L}_b\mathsf{L}_a^{-1}\mathsf{L}_b^{-1}$, from which we can derive the relation between gauge transformations:

$$\begin{aligned} & \mathsf{L}_a\mathsf{L}_b\mathsf{L}_a^{-1}\mathsf{L}_b^{-1} = \mathsf{G}_a L_a \mathsf{G}_b L_b (\mathsf{G}_a L_a)^{-1}(\mathsf{G}_b L_b)^{-1} \\ = & \mathsf{G}_a(\boldsymbol{r})(L_a \mathsf{G}_b(\boldsymbol{r}) L_a^{-1}) L_a L_b L_a^{-1} L_b^{-1} (L_b \mathsf{G}_a^*(\boldsymbol{r}) L_b^{-1}) \mathsf{G}_b^*(\boldsymbol{r}) \\ = & \mathsf{G}_a(\boldsymbol{r})\mathsf{G}_b(L_a^{-1}(\boldsymbol{r}))\mathsf{G}_a^*(L_b^{-1}(\boldsymbol{r}))\mathsf{G}_b^*(\boldsymbol{r}) = \sigma, \end{aligned} \tag{136}$$

where the conjugacy relation between a spatial operator and gauge transformation $L_a G(r) L_a^{-1} = G(L_a^{-1}(r))$ can be derived from its action on a spatial state $|r_0\rangle$,

$$\begin{aligned} & (L_a G(r) L_a^{-1})|r_0\rangle = L_a G(r)|L_a^{-1} r_0\rangle \\ = \quad & L_a G(L_a^{-1} r_0)|L_a^{-1} r_0\rangle = G(L_a^{-1} r_0)|r_0\rangle. \end{aligned} \tag{137}$$

Consider a rectangle plaquette spanned by L_x, L_y, as shown in Fig. 21(a). In the presence of gauge field, the modified translation symmetry requires the hoppings satisfy

$$t_{23} = t_{14}\mathsf{G}_a(2)\mathsf{G}_a^*(3), \quad t_{43} = t_{12}\mathsf{G}_b(4)\mathsf{G}_b^*(3).$$

Thus the phases of hoppings $e^{\phi_{ij}} = t_{ij}/|t_{ij}|$ satisfy

$$e^{i\phi_{23}} = e^{i\phi_{14}}\mathsf{G}_a(2)\mathsf{G}_a^*(3), \quad e^{i\phi_{43}} = e^{i\phi_{12}}\mathsf{G}_b(4)\mathsf{G}_b^*(3),$$

The flux surrounding the rectangle satisfies

$$\begin{aligned} e^{-i\Phi} &= e^{i\phi_{1\to2\to3\to4\to1}} = e^{i\phi_{12}}e^{i\phi_{23}}e^{i\phi_{34}}e^{i\phi_{41}} \\ &= e^{i\phi_{12}}e^{i\phi_{14}}e^{i\phi_{21}}e^{i\phi_{41}}\mathsf{G}_a(2)\mathsf{G}_a^*(3)\mathsf{G}_b^*(4)\mathsf{G}_b(3) \\ &= \mathsf{G}_a^*(3)\mathsf{G}_b^*(L_a^{-1}(3))\mathsf{G}_a(L_b^{-1}(3))\mathsf{G}_b(3) = \sigma^*. \end{aligned} \tag{138}$$

where the minus sign in exponent $e^{-i\Phi}$ comes from the convention of Peierls substitution under gauge fields $t_{ij} \to t_{ij}e^{-i\int_i^j \boldsymbol{A}d\boldsymbol{l}}$.

So when the flux in the plaquette form by L_a and L_b is Φ, the relation between L_a and L_b is modified to $\mathsf{L}_a\mathsf{L}_b\mathsf{L}_a^{-1}\mathsf{L}_b^{-1} = e^{i\Phi}$. This can be understood by the the Ahanorov-Bohm effect, that circling a region with flux Φ causes an additional phase $e^{i\Phi}$. For $\mathbb{Z}_2$ case, the nontivial $\sigma = -1$ corresponds to $\Phi = \pi$, as shown in Fig. 21.(a), where we choose a gauge that gray bonds have positive hopping amplitude and the red bonds have negative hopping amplitude.

The same argument also works for hexagon lattice as in Fig. 21.(b). In the presence of gauge field, the phases of hoppings satisfy

$$\begin{aligned} e^{i\phi_{3\to4\to7}} &= e^{i\phi_{1\to6\to5}}\mathsf{G}_x(3)\mathsf{G}_x^*(7), \\ e^{i\phi_{5\to4\to7}} &= e^{i\phi_{1\to2\to3}}\mathsf{G}_b(5)\mathsf{G}_b^*(7). \end{aligned}$$

Thus, the flux surrounding the hexagon satisfies

$$\begin{aligned} e^{-i\Phi} &= e^{i\phi_{1\to2\to3\to4\to5\to6\to1}} = \\ &= e^{i\phi_{1\to2\to3}}e^{i\phi_{3\to4\to7}}e^{i\phi_{7\to4\to5}}e^{i\phi_{5\to6\to1}} \\ &= \mathsf{G}_a(3)\mathsf{G}_a^*(7)\mathsf{G}_b^*(5)\mathsf{G}_b(7) \\ &= \mathsf{G}_a^*(7)\mathsf{G}_b^*(L_a^{-1}(7))\mathsf{G}_a(L_b^{-1}(7))\mathsf{G}_b(7) = \sigma^*. \end{aligned} \tag{139}$$

We obtain $e^{i\Phi} = \sigma$ again. This argument can be generalized to arbitrary lattice, where Φ is the flux in the foundamental domain of the translation subgroup. For some examples, see Fig. 28(a), Fig. 37(a), and Fig. 38(a).

iii. Cohomology invariants of point groups

For the factor system of point groups, we consider cohomology invariants $\alpha_r = \mathsf{R}^n$ and $\alpha_m = M^2$. As we have shown in Sec. Supplementary Note 2 a, α_r and α_m are trivial for $U(1)$ extension, so here we only discuss $\mathbb{Z}_2$ extension.

To have a nontrivial cohomology invariant α_r, n must be even. When n is even, rotating $n/2$ times is a two-fold rotation $R^{n/2} = R_\pi$. After extension, this relation in general gains an additional factor ξ, i.e., $\mathsf{R}_{2\pi/n}^{n/2} = \xi\mathsf{R}_\pi$, but it has no influence on the cohomology invariant of R_π^2:

$$\mathsf{R}^n = (\mathsf{R}^{n/2})^2 = (\xi)^2(\mathsf{R}_\pi)^2 = \mathsf{R}_\pi^2 = \alpha_r. \tag{140}$$

The equation satisfied by gauge transformation of R_π can be obtained by

$$\begin{aligned} \mathsf{R}_\pi^2 &= \mathsf{G}_{r_\pi}R_\pi\mathsf{G}_{r_\pi}R_\pi = \mathsf{G}_{r_\pi}R_\pi\mathsf{G}_{r_\pi}R_\pi^{-1}R_\pi^2 \\ &= \mathsf{G}_{r_\pi}(\boldsymbol{r})\mathsf{G}_{r_\pi}(R_\pi(\boldsymbol{r})) = \alpha_r. \end{aligned} \tag{141}$$

Consider a circuit invariant under C_n (n is even), and the circuit contains no fixed point and fixed link under C_n. We label orbits in the circuit with $i = 1, 2, 3, \cdots 2l$, then $R_\pi(i) = i + l$. The hopping amplitudes satisfy

$$t_{i,i+1} = t_{i+l,i+l+1}\mathsf{G}_{r_\pi}(i)\mathsf{G}_{r_\pi}^*(i+1).$$

Thus, the flux surrounding the circuit satisfies

$$\begin{aligned} e^{-i\Phi} &= \prod_{i=1,\mathrm{mod}\ 2l}^{2l} e^{i\phi_{i,i+1}} \\ &= \prod_{i=1,\mathrm{mod}\ 2l}^{l} \mathsf{G}_{r_\pi}(i)\mathsf{G}_{r_\pi}^*(i+1) \\ &= \mathsf{G}_{r_\pi}(1)\mathsf{G}_{r_\pi}^*(l+1) = \alpha_r. \end{aligned}$$

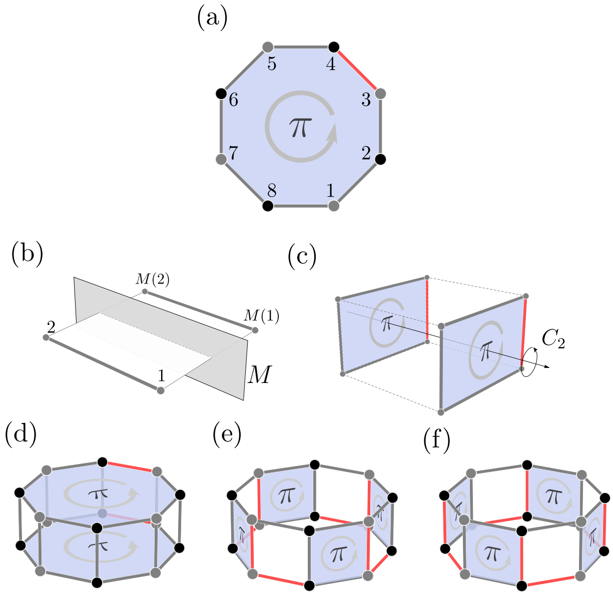

Supplementary Fig.22: Relation of fluxes and cohomology invariants of point group C_n and D_n . (a) The flux determines projective C_4 symmetry. When there is π-flux in the loop, $\mathsf{R}^4 = -1$. (b) Forbidden hoppings for $\mathsf{M}^2 = -1$. (c) Replace reflection with C_2 rotation around a horizental axis. (d)-(f) Flux determines projective D_4 symmetry. (d) Flux corresponding to the cohomology invariant of rotation. (e)(f) Flux corresponding to cohomology invariants of the two conjugacy classes of reflections (C_2 rotations around horizen axes).

We have the relation $e^{i\Phi} = \alpha_r$.

Now we consider $\mathsf{M}^2 = \alpha_m$, the equation satisfied by the gauge transformation of M can be obtained by

$$\begin{aligned} &\mathsf{M}^2 = \mathsf{G}_m M \mathsf{G}_m M = \mathsf{G}_m M \mathsf{G}_m M^{-1} M^2 \\ = \;& \mathsf{G}_m(\boldsymbol{r})\mathsf{G}_m(M(\boldsymbol{r})) = \alpha_m. \end{aligned} \tag{142}$$

However, things become more subtle now. Consider a hopping from one point i to its reflection image $M(i)$, then reflection symmetry requires the hopping satisfies

$$t_{M(i),i} = t_{i,M(i)}\mathsf{G}(M(i))\mathsf{G}(i)^* = \alpha_m t_{i,M(i)}. \tag{143}$$

For the nontrivial cohomology invariant $\alpha_m = -1$, we have $t_{i,M(i)} = -t_{M(i),i}$. If we require hoppings to be real, this condition cannot be satisfied, so this kind of hoppings (as in Fig. 22.(b)) are forbidden for $\mathsf{M}^2 = -1$ case. With this reason, the case $\mathsf{M}^2 = -1$ fails to be realized by nearest real hopping models.

In two dimensions, we can replace the reflection with a C_2 rotation around a horizental axis, as Fig. 22.(c) shows. Then the rotaion $\mathsf{R}_\pi^2 = -1$ can be realized by nearest hopping models.

For example, to realize all the possible projective algebras of D_4, we need to realize the cohomology invariants of rotations and the two conjugacy classes of reflections according to Eq. (49). We can take a bilayer version of model C_4 as illustrated in Fig. 22.(d)-(f).

iv. Cohomology invariants between translation and reflection

Now we proceed to look at the factor system between translation and reflection. The relation $\mathsf{M}_x\mathsf{L}_y\mathsf{M}_x^{-1} = \eta\mathsf{L}_y$ implies the gauge transforms satisfy

$$\begin{aligned} &\mathsf{M}_x\mathsf{L}_y\mathsf{M}_x^{-1}\mathsf{L}_y^{-1} = \mathsf{G}_m M_x \mathsf{G}_y L_y (\mathsf{G}_m M_x)^{-1}(\mathsf{G}_y L_y)^{-1} \\ = \;& \mathsf{G}_m (M_x \mathsf{G}_y M_x^{-1}) M_x L_y M_x^{-1} L_y^{-1} (L_y \mathsf{G}_m L_y^{-1})\mathsf{G}_y^{-1} \\ = \;& \mathsf{G}_m(\boldsymbol{r})\mathsf{G}_y(M_x(\boldsymbol{r}))\mathsf{G}_m^*(L_y^{-1}(\boldsymbol{r}))\mathsf{G}_y^*(\boldsymbol{r}) = \eta. \end{aligned} \tag{144}$$

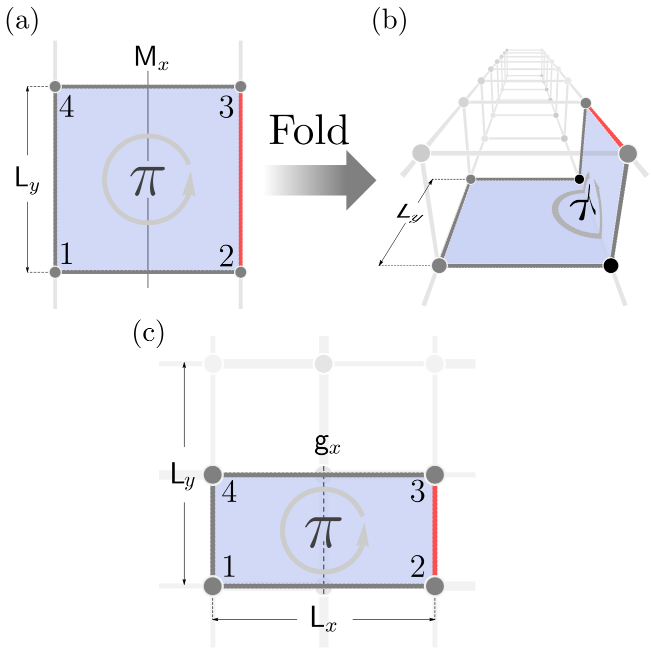

Supplementary Fig.23: The relation of fluxes and the cohomology invariants between translation and (glide-)reflection. (a) A rectangle plaquette spanned by M_x and L_y in two directions. (b) The rectangle is folded when we replace the reflection with a rotaion around a horizental axis. (c) A rectangle plaquette spanned by L_x and g_x.

Consider a rectangle plaquette spanned by M_x and L_y as illustrated in Fig. 23(a). M_x and L_y symmetry requires hoppings satisfy

$$t_{23} = t_{14}\mathsf{G}_m(2)\mathsf{G}_m^*(3), \quad t_{43} = t_{12}\mathsf{G}_y(4)\mathsf{G}_y^*(3).$$

Thus, the flux surrounding the rectangle satisfies

$$\begin{aligned} e^{-i\Phi} &= e^{i\phi_{1\to2\to3\to4\to1}} = e^{i\phi_{12}}e^{i\phi_{23}}e^{i\phi_{34}}e^{i\phi_{41}} \\ &= e^{i\phi_{12}}e^{i\phi_{14}}e^{i\phi_{21}}e^{i\phi_{41}}\mathsf{G}_m(2)\mathsf{G}_m^*(3)\mathsf{G}_y^*(4)\mathsf{G}_y(3) \\ &= \mathsf{G}_m^*(3)\mathsf{G}_y^*(M(3))\mathsf{G}_m(L_y^{-1}(3))\mathsf{G}_y(3) = \eta^*. \end{aligned} \tag{145}$$

The plaquette in Fig. 23(a) can also take a varied form. In particular, when we replace the reflection effectively by a rotation around a horizental axis, the plaquette will be folded into a three-dimensional plaquette such as that in Fig. 23(b). This also occurs in Fig. 26(b) and Fig. 30(c) in lattice models for example.

v. Cohomology invariants between translation and glide-reflection

Finally, we look at the cohomology invariant between glide-reflection and translation. The relation $\mathsf{g}_x\mathsf{L}_x\mathsf{g}_x^{-1} = \eta\mathsf{L}_x$ implies

$$\begin{aligned} &\mathsf{L}_x\mathsf{g}_x\mathsf{L}_x\mathsf{g}_x^{-1} = \mathsf{G}_xL_x\mathsf{G}_yg_x(\mathsf{G}_xL_x)(\mathsf{G}_yg_x)^{-1} \\ =\ &\mathsf{G}_x(L_x\mathsf{G}_yL_x^{-1})L_xg_xL_xg_x^{-1}((L_xg_x)^{-1}\mathsf{G}_xL_xg_x^{-1})\mathsf{G}_y^{-1} \\ =\ &\mathsf{G}_x(\boldsymbol{r})\mathsf{G}_{g_x}(L_x(\boldsymbol{r}))\mathsf{G}_x(L_xg_x^{-1}(\boldsymbol{r}))\mathsf{G}_{g_x}^*(\boldsymbol{r}) = \tau. \end{aligned} \tag{146}$$

Consider a rectangle plaquette spanned by L_x and g_x as illustrated in Fig. 23(b). The hoppings satisfy

$$t_{23} = t_{14}\mathsf{G}_x(2)\mathsf{G}_x^*(3), \quad t_{43} = t_{21}\mathsf{G}_{g_x}(4)\mathsf{G}_{g_x}^*(3).$$

Thus, the flux surrounding the rectangle satisfies

$$\begin{aligned} e^{-i\Phi} &= e^{i\phi_{1\to2\to3\to4\to1}} \\ &= e^{i\phi_{12}}e^{i\phi_{23}}e^{i\phi_{34}}e^{i\phi_{41}} \\ &= e^{i\phi_{12}}e^{i\phi_{14}}e^{i\phi_{12}}e^{i\phi_{41}}\mathsf{G}_x(2)\mathsf{G}^*_x(3)\mathsf{G}^*_{g_x}(4)\mathsf{G}_{g_x}(3) \\ &= e^{i2\phi_{12}}\mathsf{G}^*_x(3)\mathsf{G}^*_{g_x}(L_x(3))\mathsf{G}_x(L_x g_x^{-1}(3))\mathsf{G}_{g_x}(3) \\ &= \mathsf{G}^*_x(3)\mathsf{G}^*_{g_x}(L_x(3))\mathsf{G}^*_x(L_x g_x^{-1}(3))\mathsf{G}_{g_x}(3) = \tau^*. \end{aligned} \tag{147}$$

where we use the constrain of $\mathbb{Z}_2$ gauge fields, $e^{i\phi_{12}}$, $\mathsf{G}_{g_x}(\boldsymbol{r}) \in \{\pm 1\}$.

In the following, we will construct lattice models to realize all the $\mathbb{Z}_2$ projective algebras for the 17 wallpaper groups.

b. P1

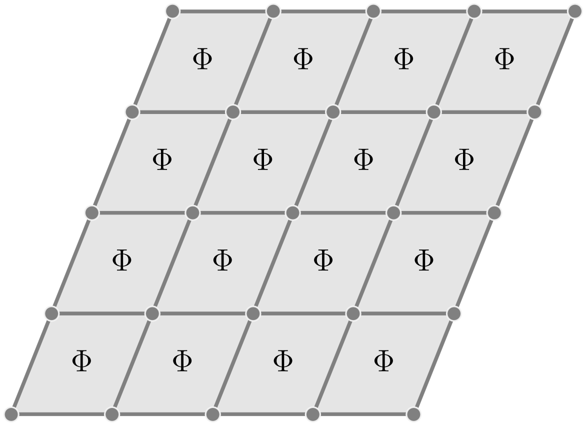

Supplementary Fig.24: Lattice with $\mathbb{Z}_2$-projective P1 symmetry. When each plaquette has $\Phi = \pi$ flux, the two translation operators become anti-commute. When $\Phi = 0$, this lattice has ordinary P1 symmetry.

The factor systems of $P1$ are labelled by the cohomology invariant $\sigma = \mathsf{L}_a\mathsf{L}_b\mathsf{L}_a^{-1}\mathsf{L}_b^{-1}$. According to what we analyzed in last section, we only need to add π-flux to each unit translation plaquette to realize the nontrivial cohomology invariant $\sigma = -1$, as shown in Fig. 24.

c. P2

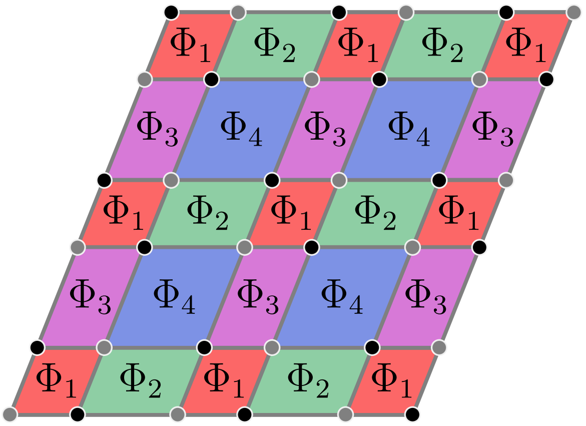

Supplementary Fig.25: An example of flux lattice with $\mathbb{Z}_2$-projective $P2$ symmetry. The fluxes corresponding to cohomology invariants $\alpha_1, \alpha_2, \alpha_3, \alpha_4$ are $\Phi_1, \Phi_2, \Phi_3, \Phi_4$.

According to Eq. (58), the $\mathbb{Z}_2^4$ classes of projective algebra of $P2$ are labelled by cohomology invariants $\alpha_1, \alpha_2, \alpha_3, \alpha_4$ of the four different rotation centers. So we construct a lattice that each class of rotation centers is surrounded by an independent plaquette, as Fig. 25 shows. Comparing with the distribution of cohomology invariants in Fig. 5, we attach flux $\Phi_1, \Phi_2, \Phi_3, \Phi_4$ to these plaquettes so that

$$e^{i\Phi_i} = \alpha_i, \quad i = 1, 2, 3, 4. \tag{148}$$

d. Pm

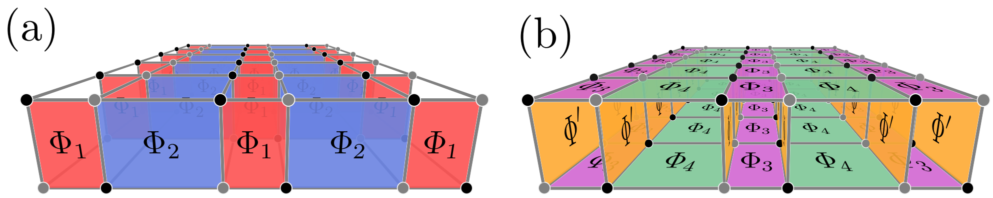

Supplementary Fig.26: An example of flux lattice with $\mathbb{Z}_2$-projective Pm symmetry. (a) The fluxes Φ_1, Φ_2 correspond to cohomology invariants β_1, β_2 of the two classes of reflections. (b) The fluxes $\Phi_3 + \Phi', \Phi_4 + \Phi'$ correspond to cohomology invariants η_1, η_2 between reflection and translations.

Since the group Pm contains reflections, to realize all the possible $\mathbb{Z}_2$- projective algebras by nearest hopping models, we need a bilayer lattice as that in Fig. 26. The reflection M_x is replaced by a rotation R_π. We will keep the notation of M_x, since the two groups are isomorphic.

According to Eq. (65), the cohomology invariants determinate $\mathbb{Z}_2^4$ classes of projective algebras of Pm. There are two kinds of conboundary invariants.

The first kind contains the cohomology invariants α_1, α_2 of reflections (Eq. (65a), Eq. (65b)), which can be realized by adding fluxes Φ_1, Φ_2 as Fig. 26(a) shows.

The second kind contains the cohomology invariants η_1, η_2, which are commutators between reflections and translations (Eq. (65c), Eq. (65d)). As discussed in Sec. Supplementary Note 3 a iv, they can be realized by adding flux $\Phi_3 + \Phi', \Phi_4 + \Phi'$ as Fig. 26(b) shows.

In summary, relations between flux distribution and cohomology invariants are given by

$$e^{i\Phi_1} = \alpha_1, \tag{149a}$$
$$e^{i\Phi_2} = \alpha_2, \tag{149b}$$
$$e^{i(\Phi_3+\Phi')} = \eta_1, \tag{149c}$$
$$e^{i(\Phi_4+\Phi')} = \eta_2. \tag{149d}$$

e. Pg

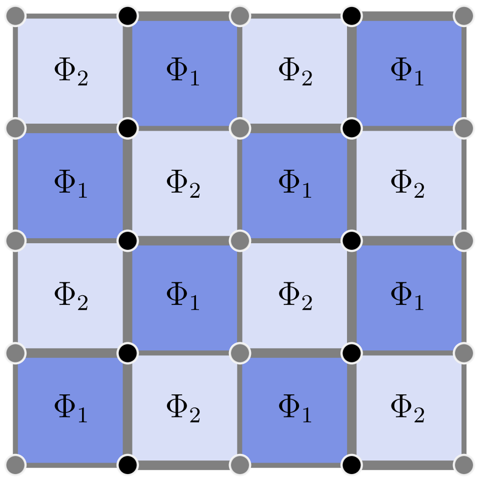

Supplementary Fig.27: An example of flux lattice with $\mathbb{Z}_2$-projective Pg symmetry.

The group Pg has only one cohomology invariant η to label factor systems between translation and glide-reflection. As discussed in Sec. Supplementary Note 3 a iv, this cohomology invariant can be realized by adding flux $\Phi = \Phi_1 + \Phi_2$ as Fig. 27.(2) shows. The relation between flux and coboundary invairant is

$$e^{i(\Phi_1+\Phi_2)} = \tau. \tag{150}$$

f. Cm

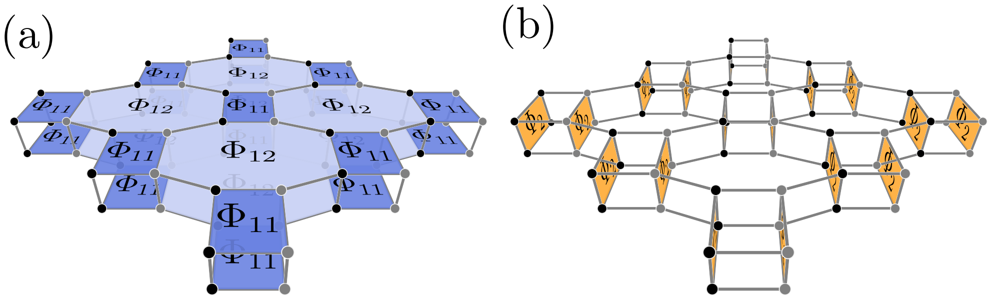

Supplementary Fig.28: An example of flux lattice with $\mathbb{Z}_2$-projective Cm symmetry. (a) The flux $\Phi_1 = \Phi_{11} + \Phi_{12}$ corresponds to the cohomology invariant of translation. (b) The flux Φ_2 correspond to the cohomology invariant of the reflection (two-fold rotation).

The group Cm also contains reflections, so we need a bilayer lattice and replace the reflection by a two-fold rotation. According to Eq. (70), two independent cohomology invariants of Cm are σ and α. The cohomology invariant σ is determined by the flux $\Phi_1 = \Phi_{11} + \Phi_{12}$ in the unit translation plaquette as Fig. 28.(a) shows. And the cohomology invariant α is determined by the flux Φ_2 in the plaquette around horizontal rotation axis as Fig. 28.(b) shows.

Relations between flux distribution and cohomology invariants are

$$e^{i(\Phi_{11}+\Phi_{12})} = e^{i\Phi_1} = \sigma, \tag{151a}$$
$$e^{i\Phi_2} = \beta. \tag{151b}$$

g. Pmm

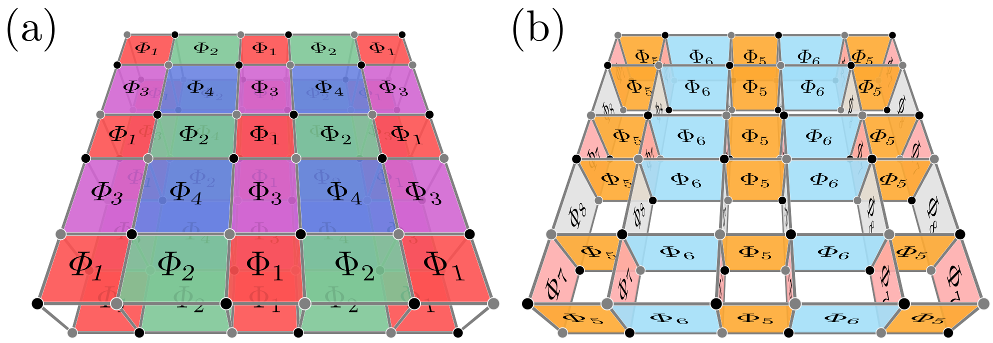

Supplementary Fig.29: An example of flux lattice with $\mathbb{Z}_2$-projective Pmm symmetry. (a) The flux $\Phi_i, i = 1,2,3,4$ correspond to cohomology invariants $\alpha_i, i = 1,2,3,4$ of rotations. (b) The flux $\Phi_i, i = 5,6,7,8$ correspond to cohomology invariants $\beta_i, i = 1,2,3,4$ of reflections (two-fold rotations).

According to Eq. (77) and Fig. 9, the $\mathbb{Z}_2^8$ classes of projective algebras of Pmm are labelled by cohomology invariants $\alpha_i, \beta_i, i = 1,2,3,4$. In order to realize all the possible projective algebras we construct a lattice in which each rotation center and reflection axis is surrounded by an independent plaquette, and we attach fluxes $\Phi_i, i = 1,2,3,4,5,6,7,8$ to it as shown in Fig. 29.

In summary, relations between flux distribution and cohomology invariants are

$$e^{i\Phi_i} = \alpha_i, \quad i = 1,2,3,4, \tag{152}$$
$$e^{i\Phi_{i+4}} = \beta_i, \quad i = 1,2,3,4. \tag{153}$$

h. Pmg

The group Pmg also contains reflections, so we need a bilayer lattice. According to Fig. 10 and Eq. (83), the $\mathbb{Z}_2^4$ classes of projective algebra of Pmg are labelled by cohomology invariants $\alpha_i, i = 1,2,\beta$ and η. Cohomology invariants α_1, α_2 are invariants of rotations, which can be realized by fluxes Φ_1, Φ_2 as illustrated in Fig. 30.(a). β is the cohomology invariant of reflection, which can realized by flux Φ_3 in the reflection invariant plaquette as Fig. 30.(b) shows. η is the cohomology invariant between translation and reflection, which can realized by fluxes $\Phi_4 = \Phi_{41} + \Phi_{42} + \Phi_{43} + \Phi_{44}$ as Fig. 30.(c) shows.

In summary, relations between flux distribution and cohomology invariants are

$$e^{i\Phi_1} = \alpha_1, \tag{154a}$$
$$e^{i\Phi_2} = \alpha_2, \tag{154b}$$
$$e^{i\Phi_3} = \beta, \tag{154c}$$
$$e^{i(\Phi_{41}+\Phi_{42}+\Phi_{43}+\Phi_{44})} = \eta. \tag{154d}$$

i. Pgg

According to Fig. 11 and Eq. (86), factor systems of Pgg can be labelled by cohomology invariants α_1, α_2 of rotation centers. Thus we attach fluxes Φ_1, Φ_2 to the plaquettes around these two rotation centers, as Fig. 31 shows.

In summary, relations between flux distribution and cohomology invariants are

$$e^{i\Phi_i} = \alpha_i, \quad i = 1,2. \tag{155a}$$

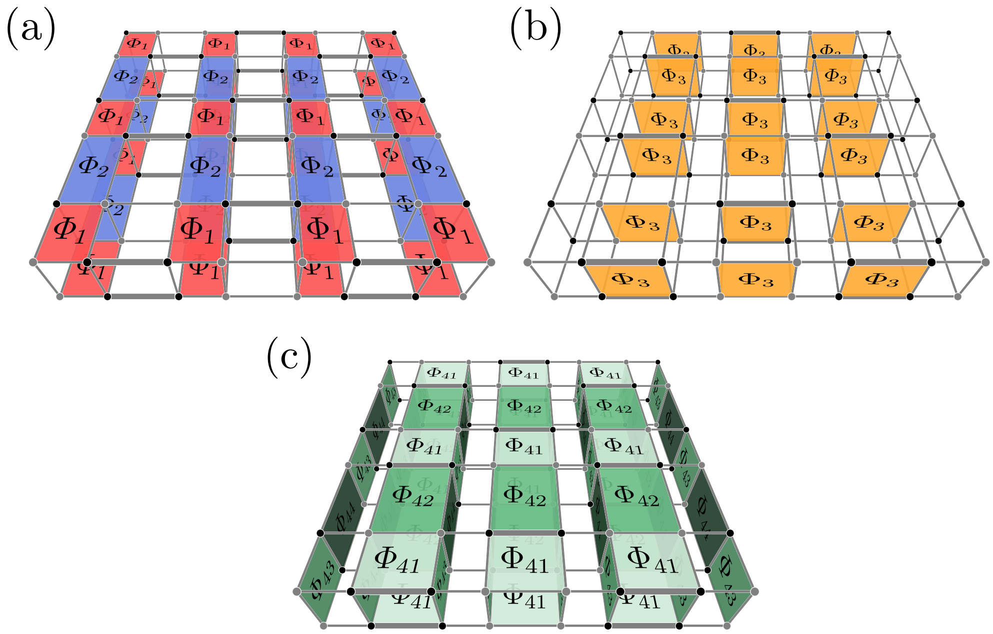

Supplementary Fig.30: An example of flux lattice with $\mathbb{Z}_2$-projective Pmg symmetry. (a) The fluxes Φ_1, Φ_2 correspond to cohomology invariants α_1, α_2 of rotations. (b) The flux Φ_3 corresponds to the cohomology invariant of reflection β. (c) The flux $\Phi_4 = \Phi_{41} + \Phi_{42} + \Phi_{43} + \Phi_{44}$ corresponds to cohomology invariant η between reflection and translation.

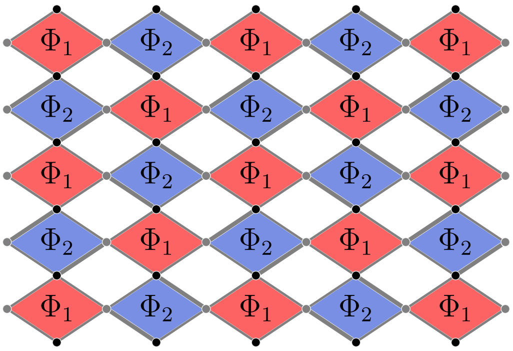

Supplementary Fig.31: An example of flux lattice with $\mathbb{Z}_2$-projective Pgg symmetry. The flux Φ_1, Φ_2 correspond to cohomology invariants α_1, α_2 of rotations.

Flux distributions are shown in Fig. 31.

j. Cmm

The group Cmm contains reflections, so we need a bilayer lattice.

According to Fig. 12 and Eq. (93), factor systems of Cmm can be labelled by cohomology invariants $\alpha_i, i = 1, 2, 3$ and $\beta_i, i = 1, 2$. To realize these cohomology invariants, we attach fluxes Φ_1, Φ_2, Φ_3 to the plaquettes around these three rotation centers as Fig. 32.(a) shows, and attach fluxes Φ_4, Φ_5 to the plaquettes around these two reflection axes as Fig. 32.(b) shows.

In summary, relations between flux distribution and cohomology invariants are

$$e^{i\Phi_i} = \alpha_i, i = 1, 2, 3, \tag{156}$$

$$e^{i\Phi_{i+3}} = \beta_i, i = 1, 2. \tag{157}$$

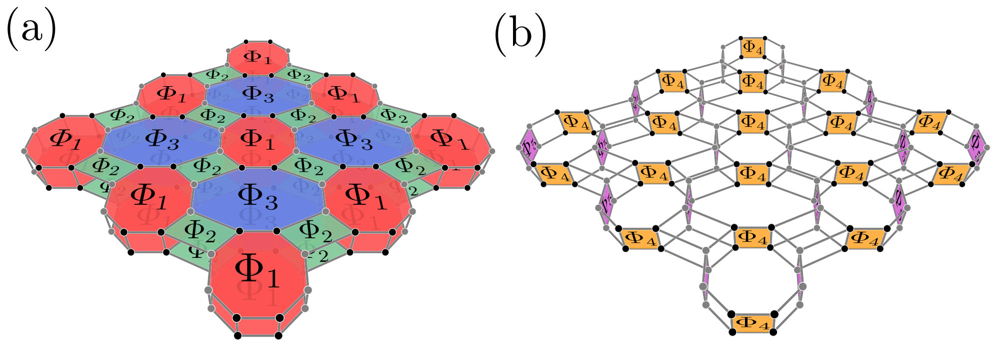

Supplementary Fig.32: An example of flux lattice with $\mathbb{Z}_2$-projective Cmm symmetry. (a) The fluxes $\Phi_i, i = 1, 2, 3$ correspond to cohomology invariants $\alpha_i, i = 1, 2, 3$ of rotations. (b) The fluxes $\Phi_i, i = 4, 5$ correspond to cohomology invariants $\alpha_i, i = 4, 5$ of reflections.

k. P4

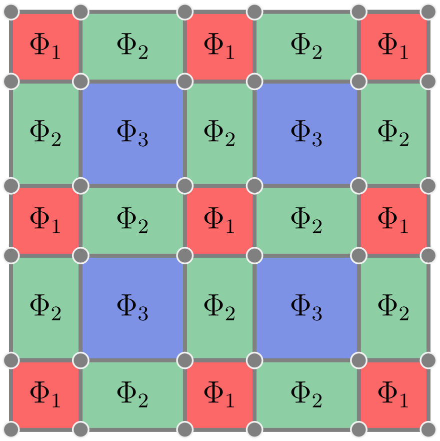

Supplementary Fig.33: An example of flux lattice with $\mathbb{Z}_2$-projective $P4$ symmetry.

According to Fig. 13 and Eq. (99), factor systems of $P4$ can be labelled by cohomology invariants $\alpha_i, i = 1, 2, 3$ of rotations. Thus we need lattice in which each rotation center is surrounded by an independent plaquette, as Fig. 33 shows.

To realize these invariants, we attach fluxes Φ_1, Φ_2, Φ_3 to the plaquettes around the three rotation centers respectively, as Fig. 33 shows.

In summary, relations between flux distribution and cohomology invariants are

$$e^{i\Phi_i} = \alpha_i, i = 1, 2, 3. \tag{158}$$

l. P4m

The group $P4m$ contains reflections, so we need a bilayer lattice.

According to Fig. 14 and Eq. (106), factor systems of $P4m$ can be labelled by cohomology invariants $\alpha_i, i = 1, 2, 3$ and $\beta_i, i = 1, 2, 3$. To realize these cohomology invariants, we attach fluxes Φ_1, Φ_2, Φ_3 to the plaquettes around the three rotation centers as Fig. 34.(a) shows, and attach fluxes Φ_4, Φ_5, Φ_6 to the plaquettes around the three reflection axes as Fig. 34.(b) shows.

In summary, relations between flux distribution and cohomology invariants are

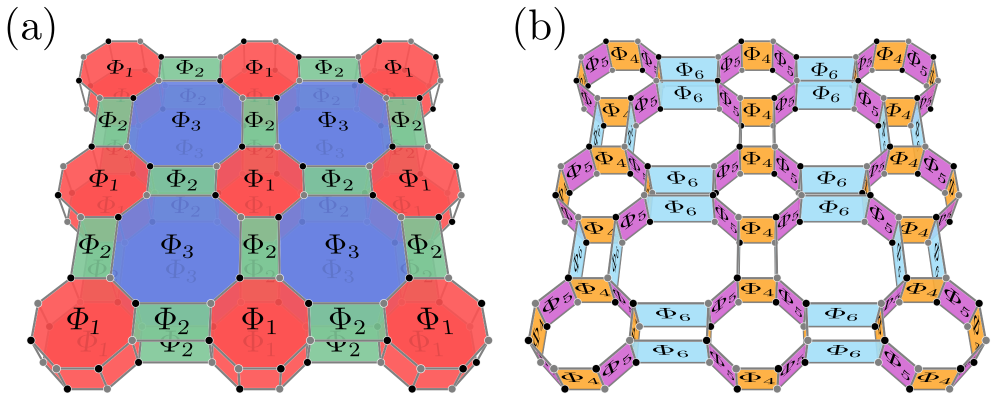

Supplementary Fig.34: An example of flux lattice with $\mathbb{Z}_2$-projective $P4m$ symmetry. (a) The fluxes $\Phi_i, i = 1, 2, 3$ correspond to cohomology invariants $\alpha_i, i = 1, 2, 3$ of rotations. (b) The fluxes $\Phi_i, i = 4, 5, 6$ correspond to cohomology invariants $\beta_i, i = 1, 2, 3$ of reflections.

$$e^{i\Phi_i} = \alpha_i, \quad i = 1, 2, 3, \tag{159}$$
$$e^{i\Phi_{i+3}} = \beta_i, \quad i = 1, 2, 3. \tag{160}$$

m. P4g

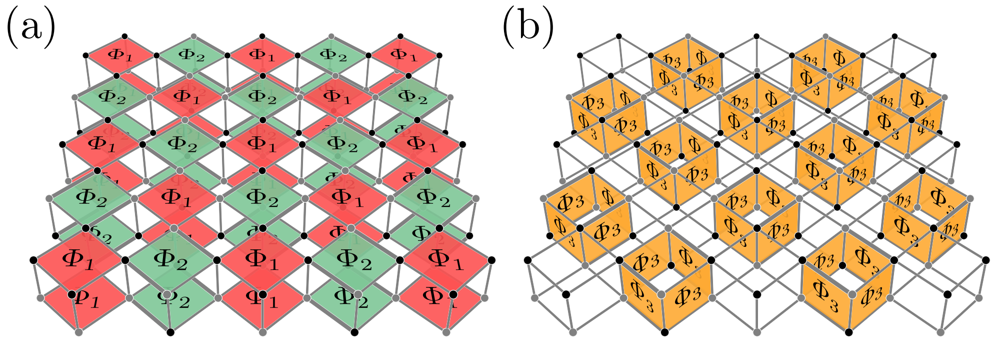

Supplementary Fig.35: An example of flux lattice with $\mathbb{Z}_2$-projective $P4g$ symmetry. (a) The fluxes $\Phi_i, i = 1, 2$ correspond to cohomology invariants $\alpha_i, i = 1, 2$ of rotations. (b) The flux Φ_3 corresponds to cohomology invariant β of reflection.

The group $P4g$ contains reflections, so we need a bilayer lattice.

According to Fig. 15 and Eq. (108), factor systems of $P4m$ can be labelled by cohomology invariants $\alpha_i, i = 1, 2$ and β. To realize these cohomology invariants, we attach fluxes Φ_1, Φ_2 to the plaquettes around these two rotation centers as Fig. 35.(a) shows, and attach fluxes Φ_3 to the plaquettes around the reflection axis as Fig. 35.(b) shows.

In summary, relations between flux distribution and cohomology invariants are

$$e^{i\Phi_i} = \alpha_i, \quad i = 1, 2, \tag{161}$$
$$e^{i\Phi_3} = \beta. \tag{162}$$

n. P3

The projective algebra of $P3$ is only depend on the cohomology invariant σ of translation. Thus, if we attach flux Φ to each plaquette as Fig. 36 shows, we can realize the projective relation Eq. (114). The relation of the cohomology invariant and flux is

$$e^{i\Phi} = \sigma. \tag{163}$$

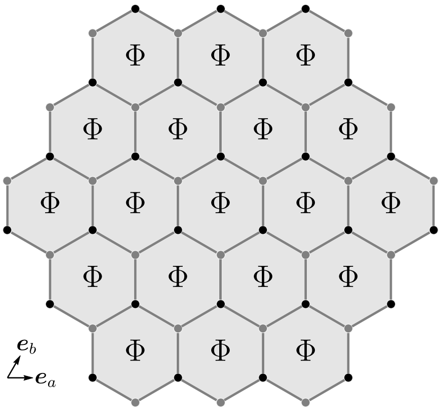

Supplementary Fig.36: An example of flux lattice with $\mathbb{Z}_2$-projective $P3$ symmetry.

o. P3m1

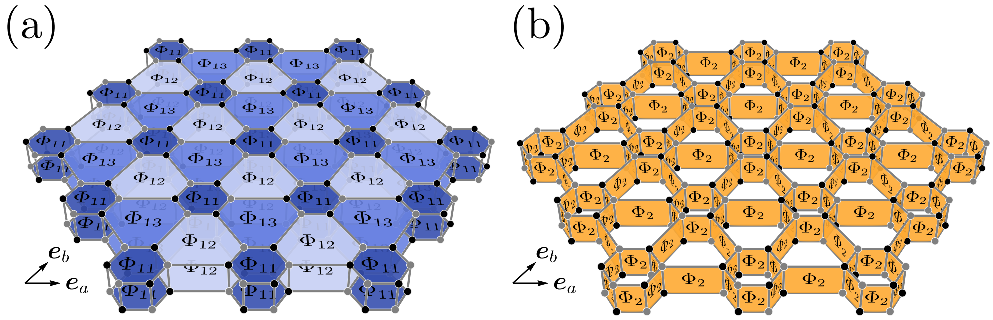

Supplementary Fig.37: An example of flux lattice with $\mathbb{Z}_2$-projective $P3m1$ symmetry. (a) The flux $\Phi_1 = \Phi_{11} + \Phi_{12} + \Phi_{13}$ in the unit translation area corresponds to the cohomology invariant σ of translation. (b) The flux Φ_2 corresponds to cohomology invariant β of reflection.

The group $P3m1$ contains reflections, so we need a bilayer lattice.

According to Eq. (117), factor systems of $P3m1$ are labelled by cohomology invariant σ and α. We attach flux Φ_1 to each unit translation plaquette as Fig. 37.(c) shows, and attach flux Φ_2 to each plaquette around the reflection axis as Fig. 37.(d) shows.

In summary, relations between flux distribution and cohomology invariants are

$$e^{i\Phi_1} = e^{i(\Phi_{11}+\Phi_{12}+\Phi_{13})} = \sigma, \tag{164a}$$
$$e^{i\Phi_2} = \beta. \tag{164b}$$

p. P31m

The group $P31m$ contains reflections, so we need a bilayer lattice.

According to Eq. (121) and Fig. 18, factor systems of $P31m$ are labelled by cohomology invariant σ and α. We attach flux Φ_1 to each unit translation plaquette as Fig. 38.(c) shows, and attach flux Φ_2 to each plaquette around the reflection axis as Fig. 37.(d) shows.

In summary, relations between flux distribution and cohomology invariants are

$$e^{i\Phi_1} = \sigma, \tag{165a}$$
$$e^{i\Phi_2} = \beta. \tag{165b}$$

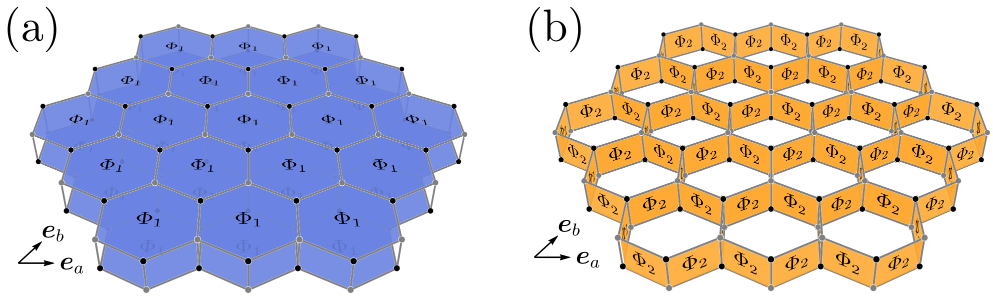

Supplementary Fig.38: An example of flux lattice with $\mathbb{Z}_2$-projective $P31m$ symmetry. (a) The flux Φ_1 in the unit translation area corresponds to coboundary the invariant σ of translation. (b) The flux Φ_2 corresponds to cohomology invariant β of reflection.

q. P6

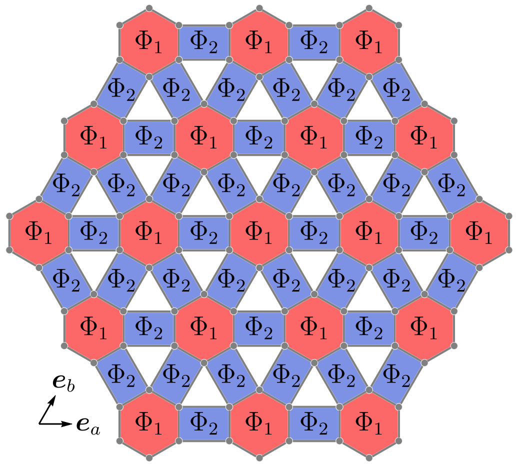

Supplementary Fig.39: An example of flux lattice with $\mathbb{Z}_2$-projective $P6$ symmetry. The fluxes Φ_1, Φ_2 correspond to cohomology invariants α_1, α_2 of rotations.

According to Eq. (127) and Fig. 19, factor systems of $P6$ can be labelled by two cohomology invariants α_1, α_2 of even-fold rotations. Thus we need a lattice in which each rotation center is surrounded by an independent plaquette. To realize the cohomology invariants, we attach fluxes Φ_1, Φ_2 to the plaquettes around these rotation centers, as Fig. 39 shows.

In summary, relations between flux distribution and cohomology invariants are

$$e^{i\Phi_i} = \alpha_i, \quad i = 1, 2. \tag{166}$$

r. P6m

The group $P6m$ contains reflections, so we need a bilayer lattice.

According to Eq. (132) and Fig. 20, factor systems of $P6m$ can be labelled by two cohomology invariants α_1, α_2 of even-fold rotations and two cohomology invariants β_1, β_2 of reflections. To realize these factors, we attach fluxes Φ_1, Φ_2 to the plaquettes around these rotation centers as Fig. 40.(a) shows, and attach fluxes Φ_3, Φ_4 to the plaquettes around these reflection axes as Fig. 40.(b) shows.

In summary, relations between flux distribution and cohomology invariants are

$$e^{i\Phi_i} = \alpha_i, \quad i = 1, 2, e^{i\Phi_{i+2}} = \beta_i, \quad i = 1, 2. \tag{167}$$

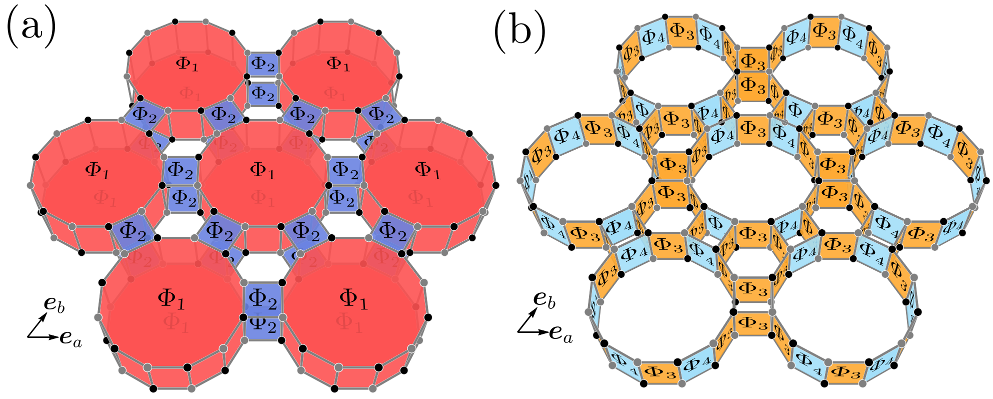

Supplementary Fig.40: An example of flux lattice with $\mathbb{Z}_2$-projective $P6m$ symmetry. (a) The fluxes $\Phi_i, i = 1, 2$ correspond to cohomology invariants $\alpha_i, i = 1, 2$ of rotations. (b) The fluxes $\Phi_i, i = 3, 4$ correspond to cohomology invariants $\beta_i, i = 1, 2$ of reflections.

Supplementary Note 4. Other technical details for results in the main text

a. Energy bands of flux lattices with all classes of $\mathbb{Z}_2$-projective P2 symmetries

In this section, we will analyze a model with projective $P2$ symmetry to show the consequences of the flux. For simplicity, we choose $\mathsf{L}_a, \mathsf{L}_b$ to be perpenticular to each other, so that $\mathsf{L}_a = \mathsf{L}_x, \mathsf{L}_b = \mathsf{L}_y$. The model is shown in Fig. 41. The parameters take values $t_1^x = 1, t_2^x = 2, t_1^y = 1.5, t_2^y = 2, m = 1$. We keep the notation that R is the rotation operator corresponding to cohomology invariant α_1.

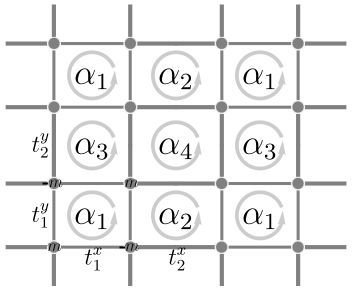

Supplementary Fig.41: Flux lattice with projective $P2$ symmetry. There are undeterminated cohomology invariants $\alpha_1, \alpha_2, \alpha_3, \alpha_4$. $t_1^x, t_2^x, t_1^y, t_2^y$ are hopping amptitudes and m is the onsite energy.

In the following, we will disucss the symmetry properties of energy bands for all classes of $\mathbb{Z}_2$-projective algebras.

(i) When cohomology invariants $(\alpha_1, \alpha_2, \alpha_3, \alpha_4) = (1, 1, 1, 1)$, the group $P2$ is linearly represented in momentum space. The time-reversal T and rotation R share the same high symmetry points Γ, X, Y, M. The energy bands are shown in Fig. 42.

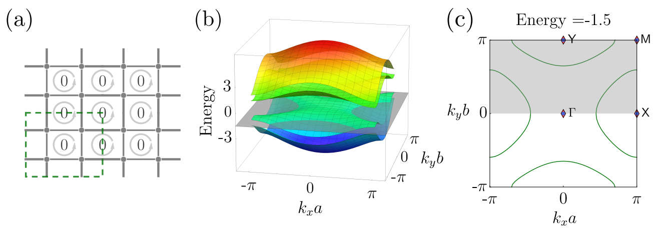

Supplementary Fig.42: Flux distribution and energy bands corresponds to $(\alpha_1, \alpha_2, \alpha_3, \alpha_4) = (1, 1, 1, 1)$.(a) Flux distribution. (b)(c) Energy bands and a constant-energy section.

(ii) When cohomology invariants $(\alpha_1, \alpha_2, \alpha_3, \alpha_4) = (-1, -1, -1, -1)$, the relations between R and L_x, L_y is maintained, so the distribution of high symmetry points in momentum space is the same as that in the case $(\alpha_1, \alpha_2, \alpha_3, \alpha_4) = (1, 1, 1, 1)$. However, at the general point , the little cogroup generator $\mathsf{R}T$ satisfies $(\mathsf{R}T)^2 = -1$. Thus the energy bands is two-fold degenerate, see Fig. 43.

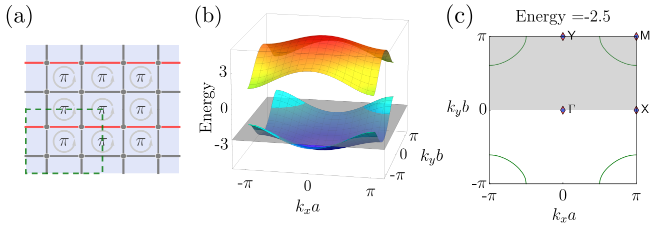

Supplementary Fig.43: Flux distribution and energy bands correspond to $(\alpha_1, \alpha_2, \alpha_3, \alpha_4) = (-1, -1, -1, -1)$.(a) Flux distributions. (b)(c) Energy bands and a constant-energy section.

(iii) When two of the cohomology invariants are negative, there are six possibilities: $(\alpha_1,\alpha_2, \alpha_3, \alpha_4) = (1, -1, 1, -1)$, $(-1, 1, -1, 1)$, $(1, 1, -1, -1)$, $(-1, -1, 1, 1)$, $(1, -1, -1, 1)$, $(-1, 1, 1, -1)$. They have the same algebra. The high symmetry points of rotation R are translated by 1/4 recipocal lattice vector compared with the high symmetry points of T. The energy bands are shown in Fig. 44.

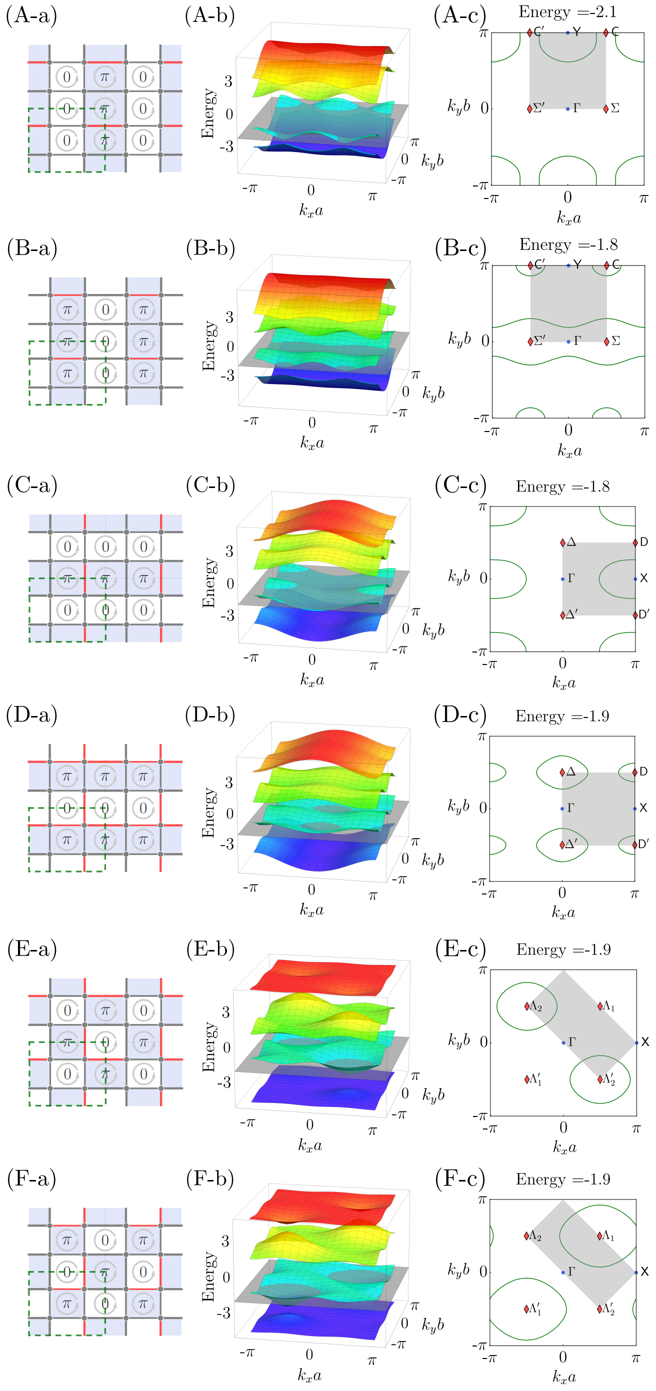

Supplementary Fig.44: Flux distributions and energy bands when two of the cohomology invariants are negative. Flux distribution corresponds to $(\alpha_1, \alpha_2, \alpha_3, \alpha_4)$ = (A-a)$(1, 1, -1, -1)$, (B-a)$(-1, -1, 1, 1)$, (C-a)$(1, 1, -1, -1)$, (D-a)$(-1, -1, 1, 1)$, (E-a)$(1, -1, -1, 1)$, (F-a)$(-1, 1, 1, -1)$. (b)(c) Energy bands and constant-energy sections.

(iv) When one of the cohomology invariants are negative, there are four possibilities: $(\alpha_1, \alpha_2, \alpha_3, \alpha_4) = (1, -1, 1, -1)$, $(-1, 1, -1, 1)$, $(1, 1, -1, -1)$, $(-1, -1, 1, 1)$, which have the same algebra. Since the original unit cell countains total π-flux, we have to enlarge the unit cell to maintain the translation symmetry of the gauge connection, as Fig. 45 shows. There are additional high symmetry points of rotation $\mathsf{L}_x\mathsf{R}$ and time-reversal L_xT, which are translated by $\boldsymbol{G}_y/4$ from the high symmetry point of R and T. The energy bands are two-fold degenerate at D, D'.

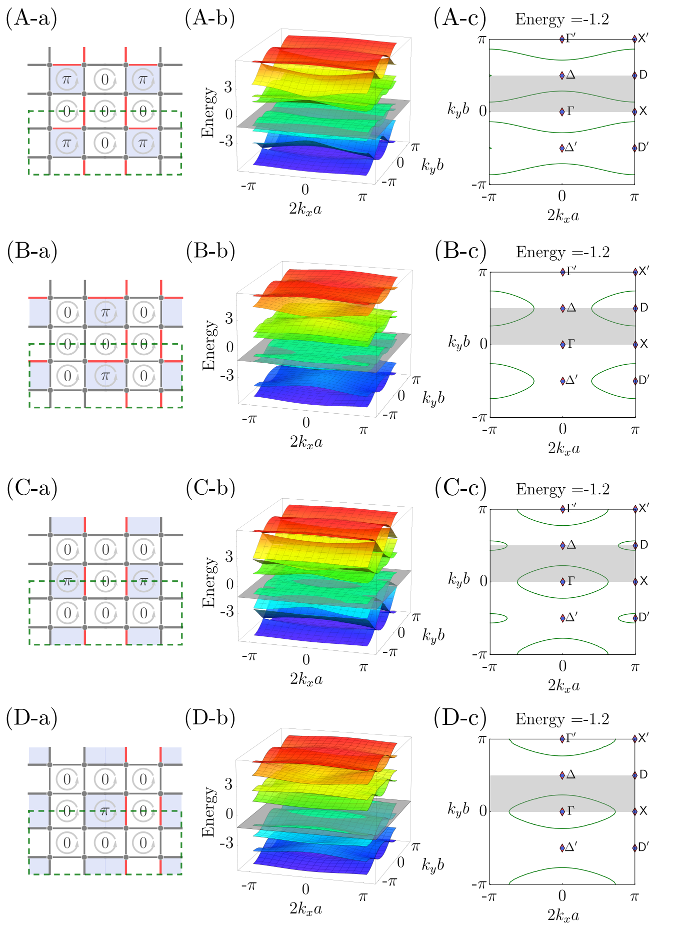

Supplementary Fig.45: Flux distributions and energy bands when one of the cohomology invariant is negative. Flux distribution corresponds to $(\alpha_1, \alpha_2, \alpha_3, \alpha_4) =$ (A-a)$(-1, 1, 1, 1)$, (B-a)$(1, -1, 1, 1)$, (C-a)$(1, 1, -1, 1)$, (D-a)$(1, 1, 1, -1)$. (b)(c) Energy bands and constant-energy sections.

(v) When three of the cohomology invariants are negative, there are four possibilities: $(\alpha_1, \alpha_2, \alpha_3, \alpha_4) = (1, -1, -1, -1)$, $(-1, 1, -1, 1)$, $(-1, -1, 1, -1)$, $(-1, -1, -1, 1)$, which has the same algebra. Since the original unit cell countains total π-flux, we have to enlarge the unit cell to maintain the translation symmetry of the gauge connection, as Fig. 46 shows. There are additional high symmetry points of rotation $\mathsf{L}_x\mathsf{R}$ and time-reversal $\mathsf{L}_x T$, which are translated by $\boldsymbol{G}_y/4$ from the high symmetry points of R and T. The energy bands are two-fold degenerate at every point.

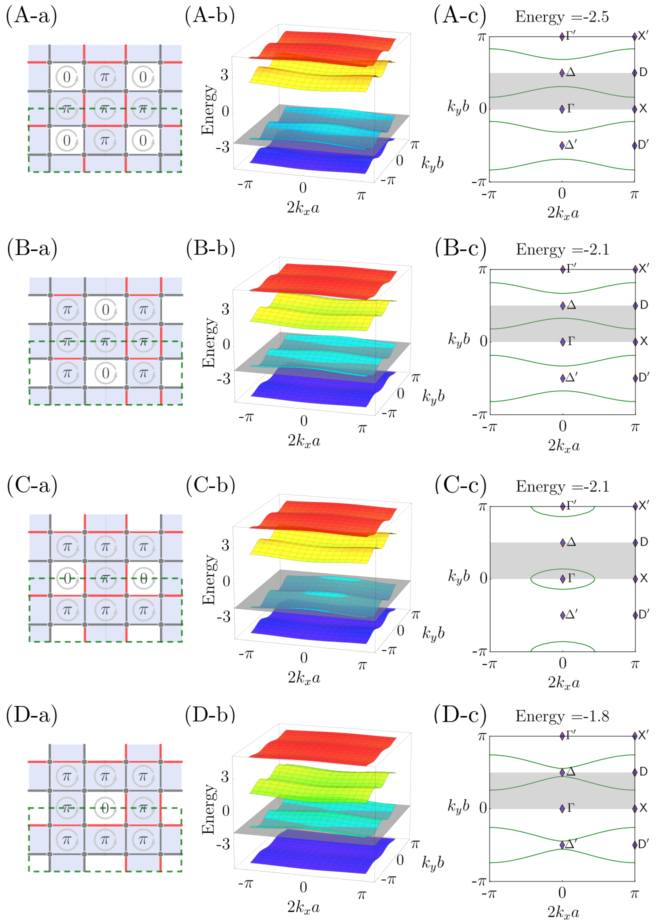

Supplementary Fig.46: Flux distributions and energy bands when three of the cohomology invariants are negative. Flux distribution corresponds to $(\alpha_1, \alpha_2, \alpha_3, \alpha_4) =$ (A-a)$(1, -1, -1, -1)$, (B-a)$(-1, 1, -1, -1)$, (C-a)$(-1, -1, 1, -1)$, (D-a)$(-1, -1, -1, 1)$. (b)(c) Energy bands and constant-energy sections.

b. Projective symmetry enforced Berry phase

Projective symmetry could enforce Berry phase along some directions to be quantized value. Consider projective $P2$ symmetrt with coboundary invariants $(\alpha_1, \alpha_2, \alpha_3, \alpha_4) = (\alpha_1, \alpha_1, -\alpha_1, -\alpha_1)$, i.e., $(\sigma, \eta_a, \eta_b, \alpha) = (1, 1, -1, \alpha_1)$. As analyzed in the last paragraph, the high-symmetry points redistribute as in Fig. 47.(a).

In this case, anti-unitary operator $\hat{\mathsf{R}}\hat{T}$ will shift momentum $\boldsymbol{k}$ to $\boldsymbol{k} + \boldsymbol{G}_y/2$ because of the nontrivial η_b. $\hat{\mathsf{R}}\hat{T}$ acts on the eigenstate of valence bands $|\psi^i_{\boldsymbol{k}}\rangle$ as

$$\hat{\mathsf{R}}\hat{T}\,|\psi^a_{\boldsymbol{k}}\rangle = \sum_b U^{ba}(\boldsymbol{k})\,|\psi^b_{\boldsymbol{k}+\frac{1}{2}\boldsymbol{G}_y}\rangle\,. \tag{168}$$

Where U is a unitary matrix of $N_{\text{occ}} \times N_{\text{occ}}$. Using this relation twice we get

$$(\hat{\mathsf{R}}\hat{T})^2\,|\psi^a_{\boldsymbol{k}}\rangle = \sum_{bc} U^{cb}(\boldsymbol{k} + \boldsymbol{G}_y/2)(U^*)^{ba}(\boldsymbol{k})\,|\psi^c_{\boldsymbol{k}}\rangle = \alpha_1\,|\psi^a_{\boldsymbol{k}}\rangle\,. \tag{169}$$

Thus,

$$U^*(\boldsymbol{k} + \boldsymbol{G}_y/2)U(\boldsymbol{k}) = \alpha I_n \tag{170}$$

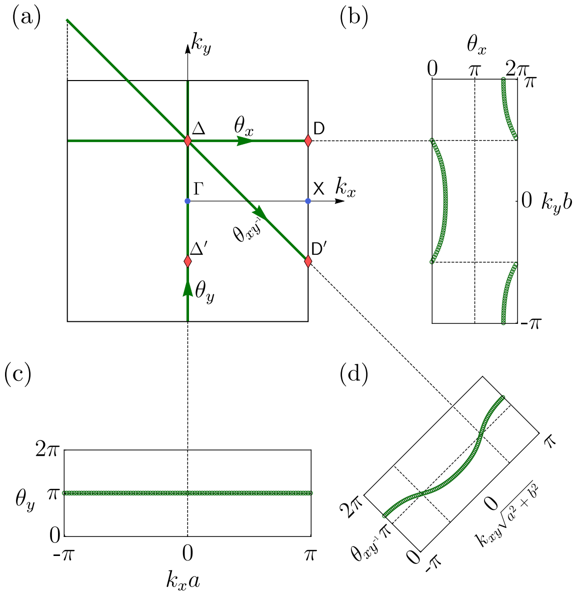

Supplementary Fig.47: Projective symmetry enforced Berry phase. (a) The Brillouin zone and high symmetry points. (b) Berry phase along $\boldsymbol{G}_x$ direction, it is quantized at $k_y b = \pm\pi/2$. (c) Berry phase along $\boldsymbol{G}_y$ direction, it is quantized at all k_x and the value is enfored to be $-i\log(\alpha_1)$. (d) Berry phase along $\boldsymbol{G}_x - \boldsymbol{G}_y$ direction, it is quantized at $k_{xy}\sqrt{a^2+b^2} = \pm\pi/2$, where k_{xy} is the projection of $\boldsymbol{k}$ in the $\boldsymbol{a}+\boldsymbol{b}$ direction.

The symmetry requires the berry connection to satisfy

$$\begin{aligned} & A_y^{ab}(\boldsymbol{k}) = i\,\langle\psi_{\boldsymbol{k}}^a|\,\frac{\partial}{\partial k_y}\,|\psi_{\boldsymbol{k}}^b\rangle = i\,\langle\hat{\mathsf{R}}\hat{T}\frac{\partial}{\partial k_y}\psi_{\boldsymbol{k}}^b|\,\hat{\mathsf{R}}\hat{T}\psi_{\boldsymbol{k}}^a\rangle = -i\,\langle\hat{\mathsf{R}}\hat{T}\psi_{\boldsymbol{k}}^a|\,\frac{\partial}{\partial k_y}\,|\hat{\mathsf{R}}\hat{T}\psi_{\boldsymbol{k}}^b\rangle \\ = & -i\sum_{cd}\langle U^{ca}(\boldsymbol{k})\psi_{\boldsymbol{k}+\boldsymbol{G}_y/2}^c|\,\frac{\partial}{\partial k_y}\,|U^{db}(\boldsymbol{k})\psi_{\boldsymbol{k}+\boldsymbol{G}_y/2}^d\rangle = -\sum_{cd}(U^\dagger)^{ac}(\boldsymbol{k})A_y^{cd}(\boldsymbol{k}+\boldsymbol{G}_y/2)U^{db}(\boldsymbol{k}) + \sum_c(U^\dagger)^{ac}(\boldsymbol{k})\frac{\partial}{\partial k_y}U^{cb}(\boldsymbol{k}) \end{aligned} \tag{171}$$

Take trace of both sides we can have

$$\mathrm{tr}\,(A_y(\boldsymbol{k}) + A_y(\boldsymbol{k}+\boldsymbol{G}_y/2)) = \mathrm{tr}\left(U^\dagger(\boldsymbol{k})\frac{\partial}{\partial k_y}U(\boldsymbol{k})\right) \tag{172}$$

Thus, the Berry phase along $\boldsymbol{G}_y$ direction as Fig.47(a) is

$$\begin{aligned} \theta_y &= \int_0^{2\pi} dk_y \mathrm{tr} A_y(\boldsymbol{k}) = \int_0^{\pi} dk_y \mathrm{tr}\,(A_y(\boldsymbol{k}) + A_y(\boldsymbol{k}+\boldsymbol{G}_y/2)) \\ &= \int_0^{\pi} dk_y \mathrm{tr}\left(U^\dagger(\boldsymbol{k})\frac{\partial}{\partial k_y}U(\boldsymbol{k})\right) = \ln\det U(\boldsymbol{k})|_{k_y=0}^{k_y=\pi} \\ &= -iN_{\mathrm{occ}}\ln\alpha \mod 2\pi. \end{aligned} \tag{173}$$

We see that θ_y is quantized to π or 0 according to $\alpha_1 = -1$ or 1 when we consider odd number of valence bands. The Berry phase along other directions will not be restricted, but will be quantized to 0 or π when they pass $\hat{\mathsf{R}}$ invariant points. One example for Berry phases is shown in Fig. 47.(b)(c)(d).

We see that although the projective algebra with $(1,1,-1,-1)$ and that with $(-1,-1,1,1)$ are isomorphic, their Berry phases θ_y are different, because Berry phase is an unit-cell dependent quantity and the unit-cell convention will break the isomorphism of the algebras. However, for the two cases, the projective symmetry groups are isomorphic, so they have the same classification of band topology. This provides us an example that isomorphic projective algebras have the same topological classification of bands but with different physical meanings.

c. High degeneracy of $P3m1$ at Γ point when $\sigma = -1$

In this section, we give the irreducible representation matrix of $P3m1$ at Γ point when it take cohomology invariant $\sigma = -1$. Since the Γ point is the highest symmetry point, the little group is the group $P3m1$ itself.

Without considering the time-reversal symmetry, there are three irreducible representations at Γ, which are given by $\Gamma_{i=1,2,3}$ in Table. II.

Irrep	Rep by generators			
	$\hat{\mathsf{L}}_a$	$\hat{\mathsf{L}}_b$	$\hat{\mathsf{R}}$	$\hat{\mathsf{M}}$
Γ_1	$i\sigma_1$	$i\sigma_3$	U_R	U_M
Γ_2	$i\sigma_1$	$i\sigma_3$	U_R	$-U_M$
Γ_3	$i\sigma_1 \otimes \sigma_0$	$i\sigma_3 \otimes \sigma_0$	$U_R \otimes D_R^3$	$U_M \otimes D_M^3$

Supplementary Table.II: Representation of $\mathbb{Z}_2^2 \rtimes D_3$ without time-reversal symmetry.

In Table. II,

$$U_R = \frac{1}{2}\begin{pmatrix} -1-i & -1+i \\ 1+i & -1+i \end{pmatrix}, \tag{174a}$$

$$U_M = \frac{i}{\sqrt{2}}\begin{pmatrix} -1 & -i \\ i & 1 \end{pmatrix}, \tag{174b}$$

$$D_R^3 = \begin{pmatrix} \cos\frac{2\pi}{3} & -\sin\frac{2\pi}{3} \\ \sin\frac{2\pi}{3} & \cos\frac{2\pi}{3} \end{pmatrix}, \tag{174c}$$

$$D_M^3 = \begin{pmatrix} 1 & 0 \\ 0 & -1 \end{pmatrix}. \tag{174d}$$

When we consider time reversal symmetry, if $\beta = 1$, irreducible representations Γ_1,Γ_2 stick together to form Γ_1^T, while the representation Γ_3 is time-reversal invariant. If $\beta = -1$, three irreducible representations $\Gamma_{i=1,2,3}$ need to be doubled to $\Gamma_{i=1,2,3}^T$ as in Table. III. The irreducible representation Γ_3^T is 8 dimensional.

Irrep	Rep by generators					α
	$\hat{\mathsf{L}}_a$	$\hat{\mathsf{L}}_b$	$\hat{\mathsf{R}}$	$\hat{\mathsf{M}}$	$\hat{T}$	
Γ_1^T	$i\sigma_1 \otimes \sigma_0$	$i\sigma_3 \otimes \sigma_0$	$U_R \otimes \sigma_0$	$U_M \otimes \sigma_3$	$\sigma_2 \otimes \sigma_2 K$	1
Γ_3^T	$i\sigma_1 \otimes \sigma_0$	$i\sigma_3 \otimes \sigma_0$	$U_R \otimes D_R^3$	$U_M \otimes D_M^3$	$\sigma_2 \otimes \sigma_2 K$	
Γ_1^T	$i\sigma_1 \otimes \sigma_0$	$i\sigma_3 \otimes \sigma_0$	$U_R \otimes \sigma_0$	$U_M \otimes \sigma_0$	$\sigma_2 \otimes \sigma_2 K$	-1
Γ_2^T	$i\sigma_1 \otimes \sigma_0$	$i\sigma_3 \otimes \sigma_0$	$U_R \otimes \sigma_0$	$-U_M \otimes \sigma_0$	$\sigma_2 \otimes \sigma_2 K$	
Γ_3^T	$i\sigma_1 \otimes \sigma_0 \otimes \sigma_0$	$i\sigma_3 \otimes \sigma_0 \otimes \sigma_0$	$U_R \otimes D_R^3 \otimes \sigma_0$	$U_M \otimes D_M^3 \otimes \sigma_3$	$\sigma_2 \otimes \sigma_2 \otimes \sigma_1 K$	

Supplementary Table.III: Representation of $\mathbb{Z}_2^2 \rtimes D_3$ with time-reversal symmetry.

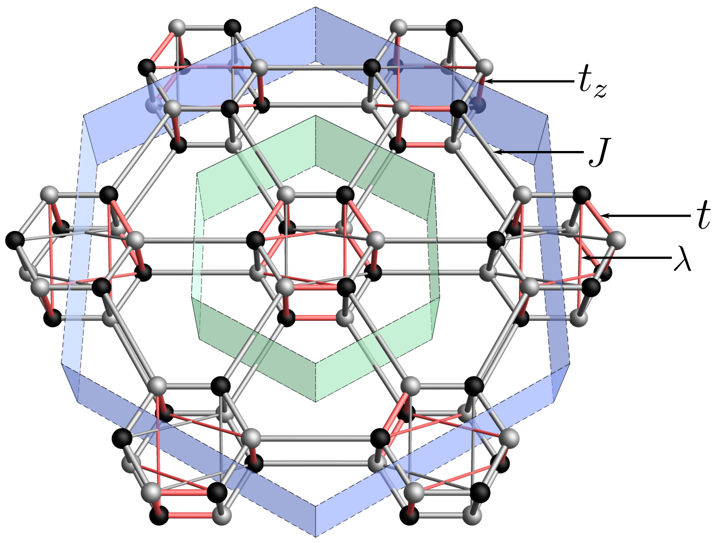

Supplementary Fig.48: Gauge convention of the lattice model of $p3m1$ correspond to cohomology invariants $\sigma = -1, \alpha = -1$. The gray(red) bonds denotes positive(negative) hoppings. When we add gauge to the model, the original unit cell(region within green boundary) is enlarged to the region within the blue boundary.

The 8 dimensional irreducible representation can be found at the lowest 8 bands of the model in Fig.38 when we take the parameters as $|t| = 2, |J| = 10, |t_z| = 5$, we also introduce a inter-layer hopping $|\lambda| = 5$ to lift the degeneracy at the general point(otherwise the bands will be four-folds degenerated at the general point).

Supplementary Note 5. Engineering gauge fluxes in artificial crystals

In this section, we first present a general mechanism, namely the so-called "dark-bright" mechanism, for generating $\mathbb{Z}_2$ gauge fields on artificial crystals, and then briefly survey on emergent $\mathbb{Z}_2$ gauge fields in various crystal systems.

a. The Dark-Bright Mechanism for Engineering $\mathbb{Z}_2$ gauge field

We would like to emphasize an important fact, i.e., $\mathbb{Z}_2$ gauge fields preserve the time-reversal symmetry, which are essentially different from other $U(1)$ gauge fields. Thus, $\mathbb{Z}_2$ gauge fields can be realized without introducing magnetism or magnetic fields. As such, $\mathbb{Z}_2$ gauge fields can be realized in low energies in a large class of lattice structures preserving time reversal symmetry. Here, we introduce the so-called dark-bright mechanism to achieve $\mathbb{Z}_2$ gauge fields.

Consider two sites with the hopping and onsite energies as $t > 0$ and ϵ in Fig. 49. The Hamiltonian of this system is written as

$$H = \begin{pmatrix} \epsilon & t \\ t & \epsilon \end{pmatrix}. \tag{175}$$

The eigen state and eigen energy can be obtained as

$$\begin{aligned} E_+ &= \epsilon + t, \qquad & |+\rangle = |a\rangle + |b\rangle\,, \\ E_- &= \epsilon - t, \qquad & |-\rangle = |a\rangle - |b\rangle\,, \end{aligned} \tag{176}$$

where $|a\rangle\,, |b\rangle$ are the local wave functions, or Wannier wave functions.

For $t > 0$, the ground state is the anti-bonding state and the excitation is the bonding state. If the sign of t is reversed as $-t$, the configuration is exchanged. By inserting an ancillary site between them with onsite energy Δ as

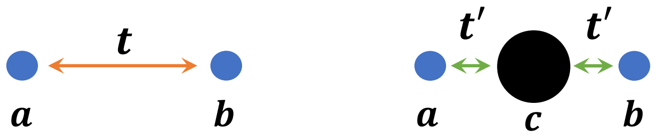

Supplementary Fig.49: Left one denotes the hopping between two sites. The onsite energy is ϵ. For the right one, an ancillary site with onsite energy as Δ and $\Delta \gg \epsilon$ is inserted between the two original sites. The hopping energy between original and inserted sites is t'.

shown in Fig. 49, the Hamiltonian is written as

$$H' = \begin{pmatrix} \epsilon & 0 & t' \\ 0 & \epsilon & t' \\ t' & t' & \Delta \end{pmatrix}. \tag{177}$$

In the limit of $\Delta \gg \epsilon, t'$, we have the eigen values and vectors as

$$\begin{aligned} E &= \epsilon, & |-\rangle &= (|a\rangle - |b\rangle)/\sqrt{2}, \\ E &\approx \epsilon - \frac{2t'^2}{\Delta - \epsilon}, & |+\rangle &\approx \left(|a\rangle + |b\rangle - \frac{2t'}{\Delta - \epsilon}|c\rangle\right)/\sqrt{2}, \\ E &\approx \Delta + \frac{2t'^2}{\Delta - \epsilon}, & |e\rangle &\approx \frac{t'}{\Delta - \epsilon}|a\rangle + \frac{t'}{\Delta - \epsilon}|b\rangle + |c\rangle. \end{aligned} \tag{178}$$

Since $\Delta \gg \epsilon, t'$, we can take $|e\rangle$ as high-energy excitation state, which is irrelevant to the energy scale of interest. The state $|-\rangle$, which is called "dark state", is decoupled with the inserted site. The state $|+\rangle$ is called "bright state". Due to $\Delta \gg \epsilon, t'$, the occupation on the inserted site can be ignored. Then, in the subspace of dark and bright states as $\{|-\rangle, |+\rangle\}$, we have the Hamiltonian as

$$H'' = \epsilon|-\rangle\langle-| + \left(\epsilon - \frac{2t'^2}{\Delta - \epsilon}\right)|+\rangle\langle+| \tag{179}$$

By taking the approximation $|+\rangle \approx (|a\rangle + |b\rangle)/\sqrt{2}$ since $|\frac{2t'}{\Delta-\epsilon}| \ll 1$, we have the effective Hamiltonian in the subspace of $\{|a\rangle, |b\rangle\}$ as

$$H_{\text{eff}} = \begin{pmatrix} \epsilon - \frac{t'^2}{\Delta-\epsilon} & -\frac{t'^2}{\Delta-\epsilon} \\ -\frac{t'^2}{\Delta-\epsilon} & \epsilon - \frac{t'^2}{\Delta-\epsilon} \end{pmatrix}, \tag{180}$$

which mimics the π hopping phase with the hopping amplitude as $\frac{t'^2}{\Delta-\epsilon}$. If we set $t'^2 = t(\Delta - \epsilon)$, we have the effective hopping coefficient between the sites a and b as $-t$. Note that the loss of the fidelity comes from the occupation on the inserted site. The higher Δ is, the better fidelity the system has.

b. A brief survey on emergent $\mathbb{Z}_2$ gauge fields in various crystal systems

In this section, we give a brief review about $\mathbb{Z}_2$ gauge fields in artificial systems, including cold atoms in optical lattices, photonic/acoustic crystals, periodic mechanical systems, electric circuit arrays, and condensed matter systems.

- In photonic crystals, the gauge field can be generated by modulation of the resonant frequencies, e.g., by adjusting the gap between site ring and link-ring wave guides.
- In acoustic crystals, $\mathbb{Z}_2$ hopping phases can be readily realized by coupling the resonators with wave guides on different sides.
- For cold atoms in optical lattices, we introduce two methods: rotating the optical lattice and laser-assisted tunneling. i) Rotating optical lattice can introduce weak and uniform effective magnetic field and the side effect of Coriolis force should be compensated. ii) For the laser-assisted tunneling, the atomic hopping with desired gauge potentials can be engineered by coupling internal levels of atoms with laser beams. Different kinds of gauge fields can be induced, even the nonabelian ones.

- For periodic mechanical systems, effective $\mathbb{Z}_2$ gauge field can be generated by tuning the stiffness coefficients of the spring connections.
- For electric circuit arrays, $\mathbb{Z}_2$ gauge fields can be realized by suitably choosing the capacitances and inductances.
- For strongly correlated systems, there are emergent gauge fields in the low-energy effective theories. The $\mathbb{Z}_2$ gauge field, which defines the $\mathbb{Z}_2$ spin liquid, can naturally emerge in quantum spin liquid. In the mean-field theory of quantum spin liquid, close to the ground states the spinors are coupled to gauge field, particularly a $\mathbb{Z}_2$ gauge field as demonstrated in several works. Actually, perhaps it was the first time that physicists noticed the importance of the projective representations of space groups with a given gauge configuration. Another example is the Kitaev-type exactly solvable model, where non-dynamical $\mathbb{Z}_2$ gauge fields are coupled with Majorana fermions.
